# A promising resilience parameter for breeding: the use of weight and feed trajectories in growing pigs

**DOI:** 10.1186/s40104-023-00901-9

**Published:** 2023-08-01

**Authors:** Wim Gorssen, Carmen Winters, Roel Meyermans, Léa Chapard, Katrijn Hooyberghs, Steven Janssens, Abe Huisman, Katrijn Peeters, Han Mulder, Nadine Buys

**Affiliations:** 1 Center for Animal Breeding and Genetics, Department of Biosystems, KU Leuven, Kasteelpark Arenberg 30 – Box 2472, 3001 Leuven, Belgium; 2 Laboratory for Biological Psychology, KU Leuven, Tiensestraat 102 - Box 3714, 3000 Leuven, Belgium; 3 Hendrix Genetics, P.O. Box 114, 5830 AC Boxmeer, The Netherlands; 4 Research Animal Breeding and Genomics, Wageningen University, P.O. Box 338, 6700 AH Wageningen, the Netherlands

**Keywords:** Deviations, Genetics, Gompertz growth curves, Heritability, Pigs, Predictive ability, Resilience, Trajectory analysis

## Abstract

**Background:**

Increasing resilience is a priority in modern pig breeding. Recent research shows that general resilience can be quantified via variability in longitudinal data. The collection of such longitudinal data on weight, feed intake and feeding behaviour in pigs has been facilitated by the development of technologies such as automated feeding stations.

The goal of this study was to investigate resilience traits, which were estimated as deviations from longitudinal weight, feed intake and feeding behaviour data during the finishing phase. A dataset with 324,207 records between the age of 95 and 155 days on 5,939 Piétrain pigs with known pedigree and genomic information was used. We provided guidelines for a rigid quality control of longitudinal body weight data, as we found that outliers can significantly affect results. Gompertz growth curve analysis, linear modelling and trajectory analyses were used for quantifying resilience traits.

**Results:**

To our knowledge, this is the first study comparing resilience traits from longitudinal body weight, feed intake and feeding behaviour data in pigs. We demonstrated that the resilience traits are lowly to moderately heritable for deviations in body weight (*h*^2^ = 2.9%–20.2%), in feed intake (9.4%–23.3%) and in feeding behaviour (16.2%–28.3%). Additionally, these traits have good predictive abilities in cross-validation analyses. Deviations in individual body weight and feed intake trajectories are highly correlated (*r*_g_ = 0.78) with low to moderate favourable genetic correlations with feed conversion ratio (*r*_g_ = 0.39–0.49). Lastly, we showed that some resilience traits, such as the natural logarithm of variances of observed versus predicted body weights (lnvar_weight_), are more robust to lower observation frequencies and are repeatable over three different time periods of the finishing phase.

**Conclusions:**

Our results will help future studies investigating resilience traits and resilience-related traits. Moreover, our study provides first results on standardization of quality control and efficient data sampling from automated feeding station data. Our findings will be valuable for breeding organizations as they offer evidence that pigs’ general resilience can be selected on with good accuracy. Moreover, this methodology might be extended to other species to quantify resilience based on longitudinal data.

**Supplementary Information:**

The online version contains supplementary material available at 10.1186/s40104-023-00901-9.

## Background

Resilience in livestock usually refers to the ability of animals to be minimally affected by (environmental) stressors and/or to cope with these stressors and quickly return to their optimal production level [1–5]. As such, resilience is becoming an important breeding goal in pig breeding [6]. Increasing resilience is particularly interesting as it can simultaneously tackle animal welfare concerns, reduce labor and treatment costs [1, 5–8]. Moreover, the need for robust, easy-to-handle animals rises with an increased number of animals per farmer. This is evidenced in the European Union, where the average size of pig farms keeps growing [9]. Although the need for more resilient pigs is evident, it has been difficult and/or costly to phenotype informative traits for pigs’ (general) resilience [7]. On one hand, most routinely phenotyped resilience indicators are scored as binary (e.g., ‘dead’ versus ‘alive’) or ordinal (e.g., ‘no’, ‘mild’ or ‘severe’ disease) traits. These traits often have low frequencies, with low variability and low heritabilities [10, 11]. On the other hand, immunological traits, such as viral load or antibody levels, show moderate to high heritabilities and a good association with animal health, but are costly to phenotype and in practice challenging to obtain [12, 13].

Recently however, several studies showed that increasing within-family and within-individual uniformity can improve animals’ general resilience. Blasco et al. [14] and Formoso-Rafferty et al. [15] independently executed two successful selection experiments on respectively litter size uniformity in rabbits and birth weight uniformity in mice. For lines with increased (within-family) uniformity, both studies found a correlated selection response with a higher survival in these uniform lines and a favorable association with disease susceptibility traits. Scheffer et al. [5] and Berghof et al. [1] proposed to derive resilience traits from longitudinal phenotypes by quantifying the variability in longitudinal data. Here, the hypothesis is that animals with a higher within-individual uniformity over time will have a higher resilience as they will show less deviations from their optimal production level in the presence of (environmental) disturbances [1, 5]. Recent studies have reported that these within-individual deviations of longitudinal data are lowly to moderately heritable (Table 1). Moreover, these studies generally found favourable genetic correlations between within-individual uniformity and resilience-related traits, such as mortality and disease incidence. Hence, less deviations in longitudinal data (higher within-individual uniformity), was linked with higher survival and lower disease incidence (Table 1). However, the number of studies investigating this relationship is currently limited.

**Table 1:** Overview of genetic studies on within-individual trait deviations based on longitudinal data

Reference	Species	Deviations in trait	*h*^2^	Favorable genetic correlations (*r*_g_) with resilience-related traits
[16, 17]	Pig	Feed intake	8%–26%	Mortality (*r*_g_ = 0.37–0.75); Number of therapeutic treatments (*r*_g_ = 0.56–0.85)
[18]	Pig	Feed intake	31%	-
[18]	Pig	Time spent at feeder	36%	-
[18]	Pig	Number of visits to feeder	40%	-
[19]	Pig	Feed intake	7%–11%	-
[19]	Pig	Time spent at feeder	16%–20%	-
[20]	Pig	Body weight	3%–4%	-
[21]	Pig	Body weight	31%	-
[22]	Cattle	Milk yield	6%–10%	Udder health (*r*_g_ = −0.36); ketosis (*r*_g_ = −0.52); longeveity (*r*_g_ = −0.30); persistency (*r*_g_ = −0.29)
[23]	Cattle	Milk yield	1%–24%	Udder health (*r*_g_ = −0.22 to −0.32); ketosis (*r*_g_ = −0.27 to −0.33); body condition score (*r*_g_ = −0.29 to −0.40)
[24]	Chicken	Body Weight	9%–11%	Favorable association between estimated breeding values and lesion scores
[25]	Chicken	Egg production	10%–12%	-

Thanks to technological developments, longitudinal data can be collected on a large scale in practice [5]. For instance, the use of automatic feeding stations (AFS) enables individual recording of pigs’ feed intake, feeding behaviour (duration and time of visits) and body weight. Despite the elevated cost of AFS, most pig breeding organizations have invested in this technology [1]. In addition, advances in wearables and computer vision systems may create longitudinal data in pigs for a variety of traits [4, 5] including body temperature, respiration rate [26] and activity levels [27]. The integration of genomics and other ‘omics’ techniques could further aid the development of efficient selection programs for increased resilience [7].

In this study we will investigate the genetic background of resilience proxies based on longitudinal body weight, feed intake and feeding behaviour data in a Piétrain pig population. This study is the first to examine the value of body weight deviations based on trajectory analysis as a novel proxy for resilience. Moreover, it is unique in its comparison and analysis of deviations in weight, feed intake and feeding behaviour over time, as previous studies have only focused on deviations in weight, feed intake or feeding behaviour. Lastly, we investigate the influence of observation frequency on the stability of resilience traits and the influence of observation period on the repeatability of resilience traits. Therefore, the repeatability of resilience traits over different stages of the finishing period (observation period) was studied as well as the impact of less data points per individual (observation frequency). Genetic parameters, such as heritability, genetic coefficient of variation and genetic correlations are estimated for the resilience traits. In an effort to better understand the value of genomics in selection for resilience, we assessed the predictive abilities using pedigree relationships or single-step genomic evaluation.

## Methods

### Animals and data collection

The study was carried out on Piétrain pigs from Hendrix Genetics (Hypor Maxter). The nucleus pig test barn (France) consisted of 14 compartments with 10 pens per compartment and on average 15 pigs per pen (1.0 m^2^ per pig). Water was provided ad libitum in each pen from one nipple drinker and feed was provided with an automatic feeding system (AFS): Nedap pig performance testing feeding station (Nedap N.V.; Groenlo, the Netherlands). Individual recordings of weight (accuracy of 0.5 kg), feed intake (accuracy of 1 g), visit duration (accuracy of 1 s) and number of visits were obtained with the AFS per day. The daily records were calculated as summary statistics based on a pigs’ daily feeding station visits. Before data quality control (QC), the dataset comprised of 7,880 pigs born between May 2017 and September 2021. In total, these pigs had 522,122 AFS recordings for weight, feed intake, feeding duration and number of visits (on average 66 records per pig for each trait). Moreover, for all these pigs with AFS recordings, individual weights were also recorded by technicians at birth, 14 days of age, start of test (81 ± 5 d and 32.6 ± 7.6 kg) and end of test (161 ± 12 d and 114.3 ± 13.1 kg). At the end of test, muscle thickness and fat thickness were measured via ultrasound probing between 3^rd^ and 4^th^ last rib using Exago (IMV) device for these pigs.

### Quality control

This study investigates variability in longitudinal data from AFS, and links this variability with underlying biological/genetic factors. Therefore, it is vital that variability due to technical errors and/or noise are removed as much as possible. In a first step of quality control, outlier correction limits were designed based on population statistics to identify and exclude gross weight recording inaccuracies. Specifically, AFS weight recordings below 10 kg (*n* = 874) or above 160 kg before an age of 160 days (*n* = 9,339) were set to missing (511,909 AFS weight records retained). Additionally, only pigs with at least twenty AFS weight recordings were retained to ensure a sufficient number of records per individual for the accurate estimation of resilience traits. After this first step of QC, 6,831 pigs and 505,990 AFS weight records were retained.

**Fig. 1:**
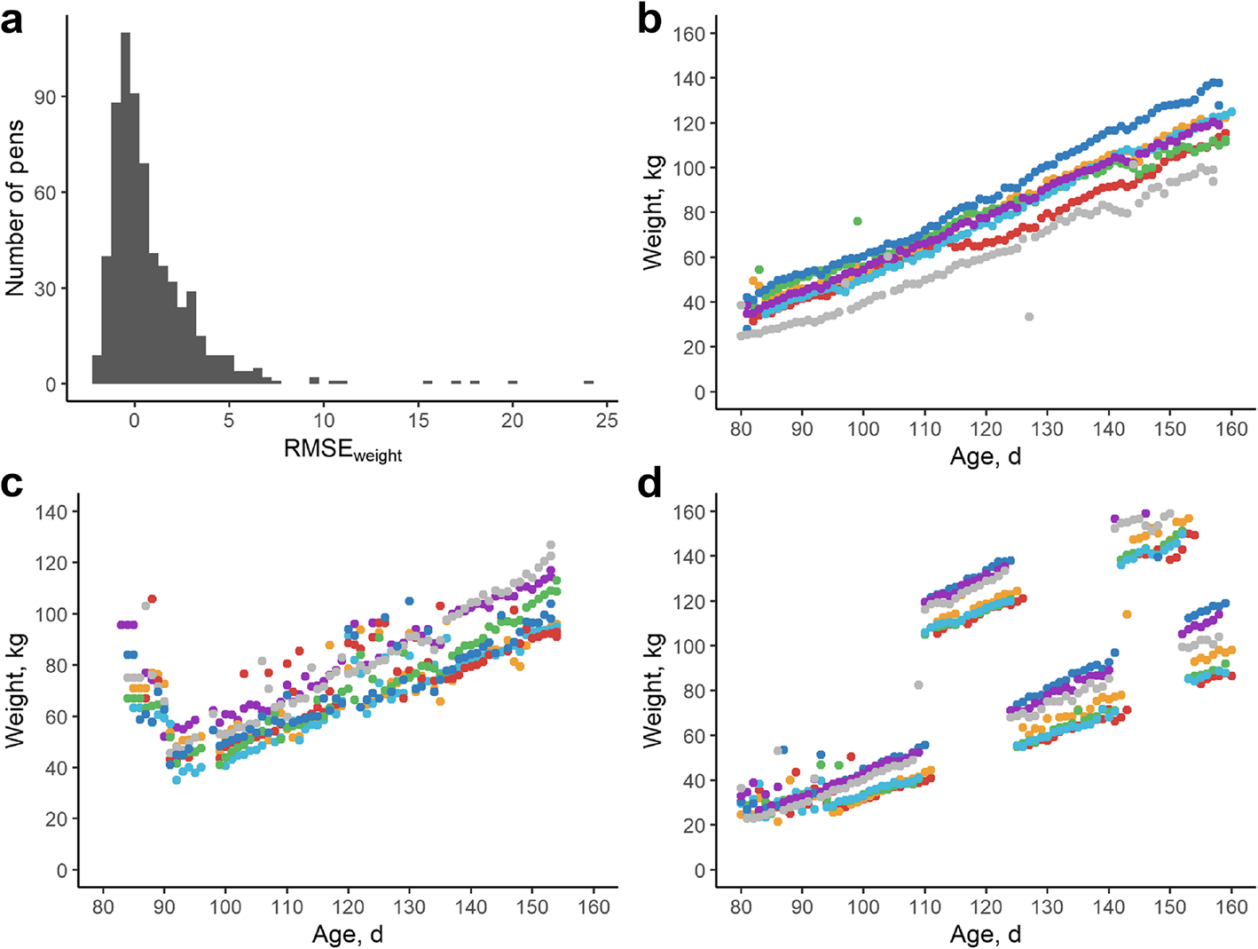
Outlier detection on pen level by analyzing root mean squared error of weight (RMSE_weight_). **a** Histogram of RMSE_weight_ in function of age on a pen level before quality control. Pens with high RMSE_weight_ estimates were visually inspected for (technical) errors. **b** Example of a pen with no severe outlying weights at the pen level, although some individual weight recordings are outlying. Weight evolution of individual pigs are represented with a specific color. **c** Example of a pen with outlying weights at start of trajectory. Such outliers are often due to an adaptation phase of the pigs, i.e., upon entering the automated feeding station, pigs tend to enter the station with their penmates, inflating the daily weight estimates. Weight evolution of individual pigs are represented with a specific color. **d** Example of technical issues causing outlying weights and high RMSE_weights_. In these cases, outliers were set to missing, or outlying individuals were removed from the dataset. Weight evolution of individual pigs are represented with a specific color

**Fig. 2:**
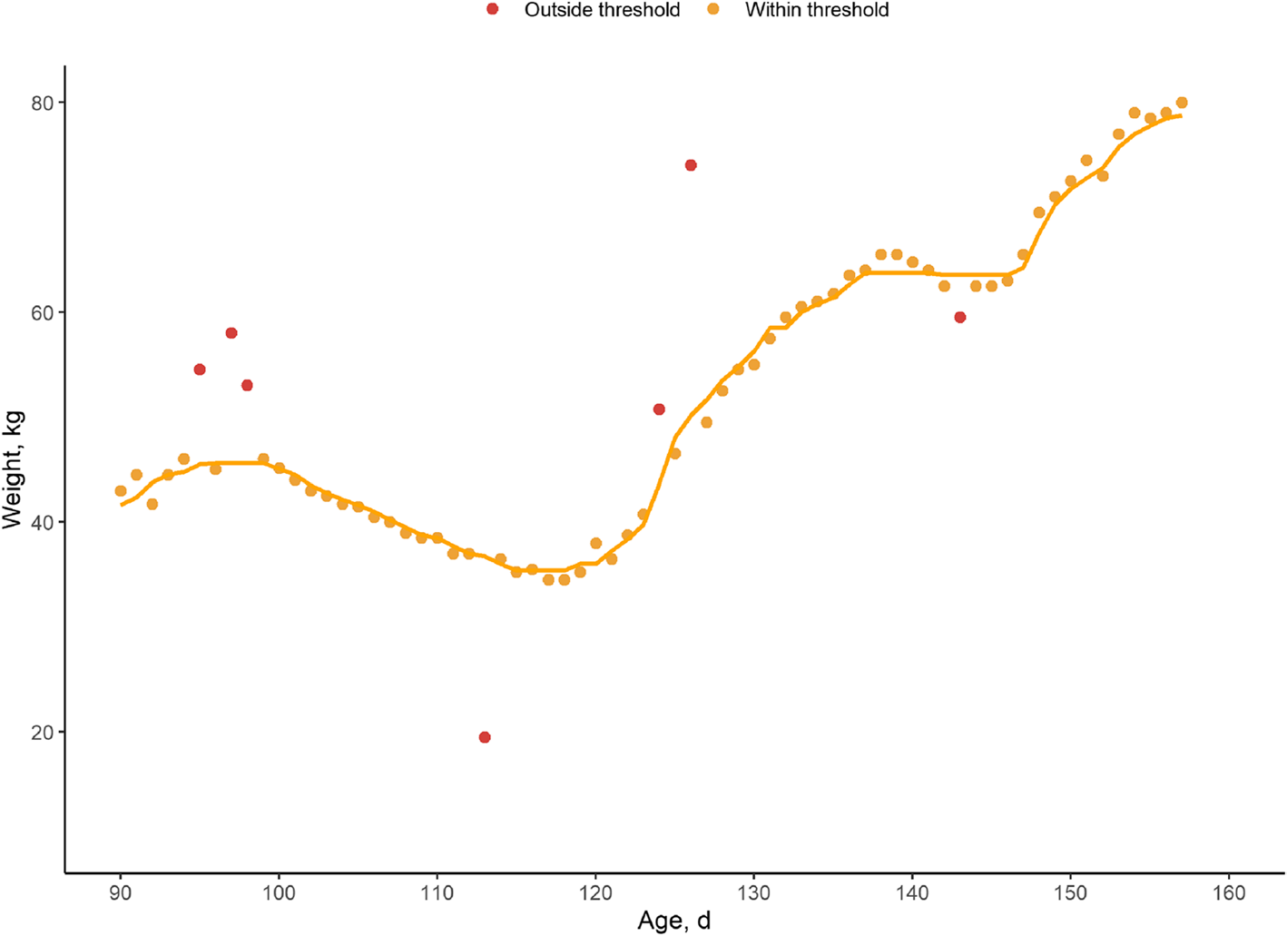
Example of 10-d rolling median approach combined with second order polynomial regression to detect outliers. Observed weights outlying predicted weight ± 3 kg were considered as outliers (red) and set to missing

Next, inaccurate AFS weight recordings were identified on a pen level using the root mean square error (RMSE), similar to [20]. RMSE was obtained by linear regression of weight on age. Weight records of individuals in outlying pens were visually inspected (Fig. 1) and treated as follows: (i) erroneous weight recordings within a time period < 20 d were set to missing; (ii) Individuals with erroneous weight recordings over a longer time period were removed from the dataset (6,788 pigs and 501,320 AFS weight records retained). Next, a 10-day rolling median weight was calculated per individual. Weight recordings deviating more than 3 kg from this median rolling weight were considered as outliers and set to missing (Fig. 2; 6,728 pigs and 495,312 AFS weight records retained). Furthermore, pigs with gaps in weight recordings larger than ten days were removed. Hereafter, the RMSE of weight regressed on age was re-calculated, and outlying individuals were checked again. After these QC steps, the dataset contained 6,457 pigs, 439,963 weight recordings (82% of pigs and 84% of records before QC).

For daily feed intake (FI), visit duration and number of visits, values exceeding the average plus four times the standard deviation were set to missing (5,550 g/d for FI; 3.3 h/d for visit duration; 33 visits/d for number of visits). Hereafter, individual and pen RMSE were obtained for these traits by regressing them on age. However, no outliers were detected using this method. After these QC steps, the dataset contained 6,457 pigs with 438,132 feed intake records, 437,753 visit duration records and 436,886 number of AFS visit records.

**Fig. 3:**
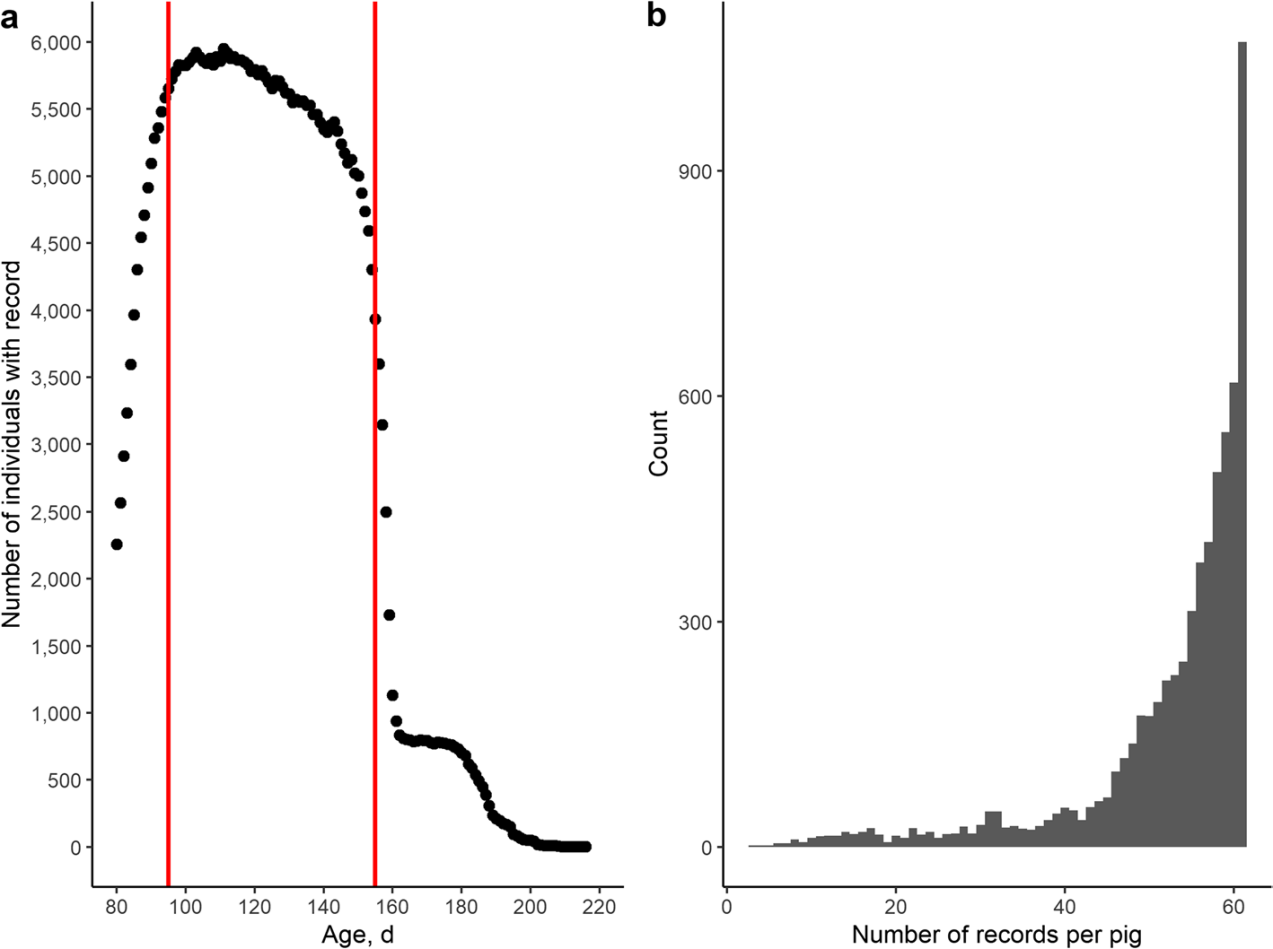
Distribution of number of weight records. **a** Number of individuals with automatic feeding station weight recordings in function of age (d). Red lines indicate thresholds of 95 and 155 d. **b** Histogram of number of records per pig after selecting the age range of 95–155 d. The maximum amount of records is 60

**Fig. 4:**
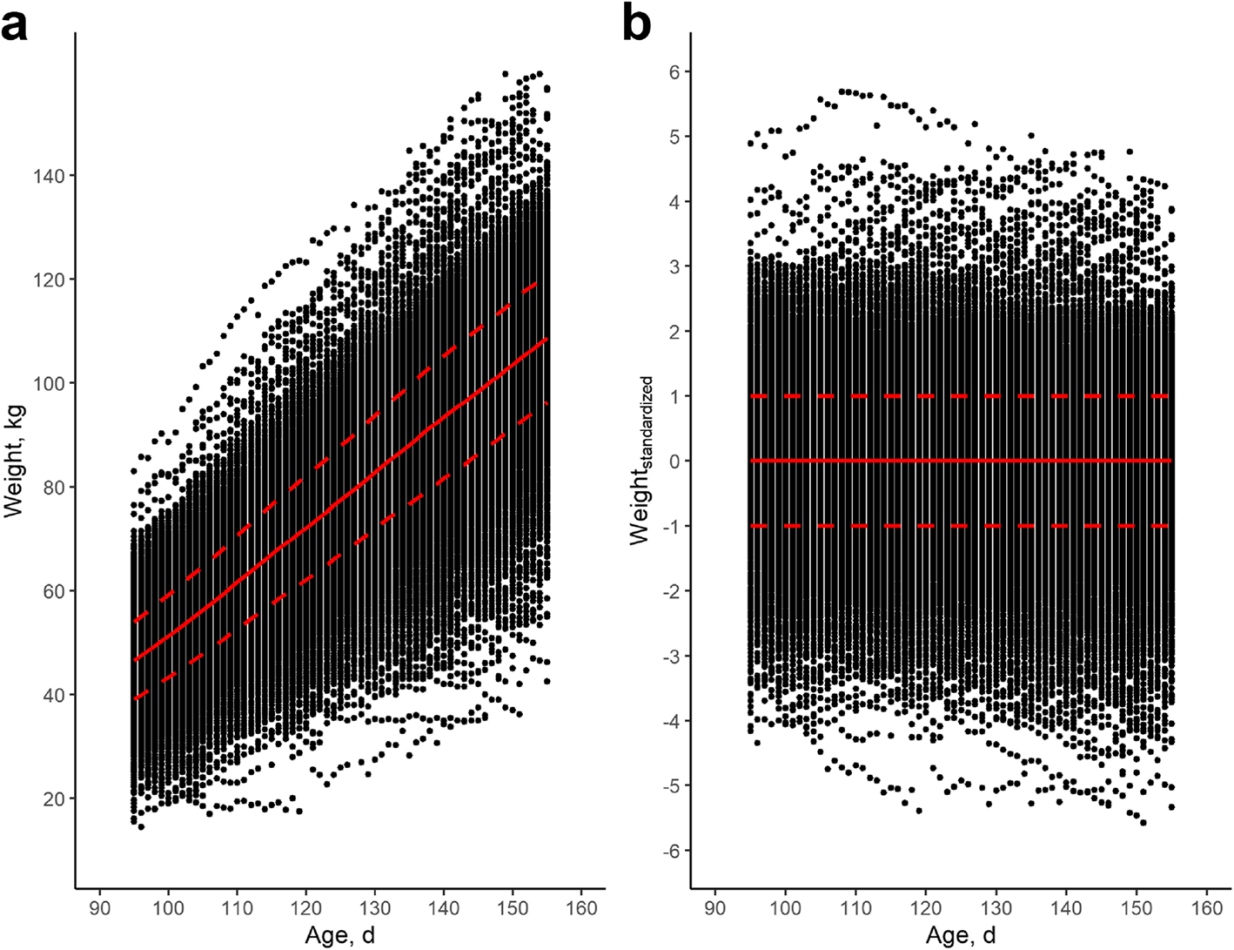
Evolution of weight and standardized weight in function of age. **a** Evolution of weight in kg in function of age in d for the dataset after quality control. The mean weight per age (d) is shown in solid red line, a one standard deviation difference from the mean is shown in dashed red lines. **b** Standardized weights with a mean of zero and a standard deviation of 1 per age in days. For example, a score of ‘2’ indicates a pig had a weight which was two standard deviations above the mean of the population on that specific age. The mean standardized weight per age (d) is shown in solid red line, a one standard deviation difference from the mean is shown in dashed red lines

Next, only AFS records were kept between an age of 95–155 d to standardize age limits across animals. These thresholds were selected because most of our AFS recordings fall within this range (Fig. 3) and because most pigs show a learning curve after entering the pen with AFS, which disappears around d 95 in our dataset. Finally, data were further standardized by removing pigs with (i) starting age > 110 d (*n* = 75), (ii) maximum age < 120 d (*n* = 226 pigs), (iii) > 30% missing records for weight or feed intake (*n* = 240 pigs) and (iv) < 20 d with AFS records (*n* = 188 pigs). The final dataset after QC comprised 5,939 pigs (5,811 boars and 128 sows) with 324,478 AFS weight recordings, 323,775 feed intake recordings, 323,304 visit duration recordings and 322,910 number of visit recordings between 95 and 155 days of age (75% of pigs and ~ 62% of records before QC). The pigs originated from 1,273 dams and 130 sires (2,105 unique litters). Pedigree consisted of 9,369 pigs with a pedigree depth ranging from 13 to 19 generations. Genomic information (45,436 SNPs) was available for 6,726 pigs in total, of which 5,160 pigs (87% of dataset) had own phenotypic records. The evolution of weight in function of age for data after QC is shown in Fig. 4a.

### Derivation of traits

After QC, traits were operationalized. Average daily gain (ADG) was estimated as

$$ADG\;(kg/d)=\frac{weight\;\left(kg\right)\;at\;maximum\;age\;AFS-weight\;(kg)\;at\;minimum\;age\;AFS}{maximum\;age\;AFS\;\left(d\right)-minimum\;age\;AFS\;(d)}$$

Average feed intake (AFI) per individual was estimated as total feed intake divided by number of days with a feed intake record. Feed conversion ratio (FCR) was estimated by dividing ADG by AFI.

**Fig. 5:**
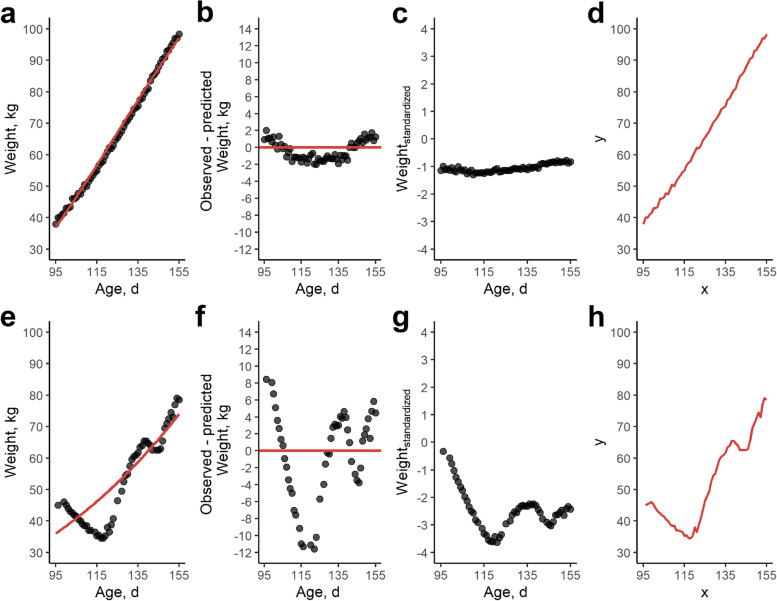
Example of trait construction for two pigs (**a–d** versus **e**–**h**). The upper pig (**a–d**) showed little deviations in observed versus expected body weight, whereas the lower pig (**e**–**h**) showed many deviations in observed versus expected body weight. These examples are the same animals as shown in Fig. 6. **a** and **e** Example of Gompertz growth curve modelling on automated feeding station data of individual pigs. The Gompertz growth curve is shown as a solid red line, observed daily weights are given as black dots. **b** and **f** Deviations of observed versus predicted weights after Gompertz modeling: lnvar_weight_, lag1_weight_ and skew_weight_ are estimated based on these deviations. **c** and **g** Example of standardized weights with mean zero and standard deviation one for the population on a daily basis. The variance of these standardized weights for an individual was used to calculate lnvar_weight_standardized_. **d** and **h** Trajectory analysis of weight. Here, weight gain/loss is seen as a trajectory from start until end, with age in d as *x*-coordinate and weight as *y*-coordinate. From this trajectory, mean speed and straightness were calculated as resilience traits

An overview of resilience trait definitions is given in Table 2. A number of resilience traits was operationalized based on deviations in weight trajectories. First, this was established by individually fitting a Gompertz growth curve [28] based on AFS weights between 95–155 days of age, supplemented with birth weight, weight at 14 d, weight at start and end of test. Expected weights were estimated with R [29] using the *nls* function and the Gompertz growth curve formula (Additional file 1: Fig. S1):

$${weight}_{ij}={A}_{i}\times{e}^{-{B}_{i}\times{e}^{{k}_{i}\times{t}_{ij}}}+{\varepsilon }_{ij}$$

where *A*_*i*_, *B*_*i*_ and *k*_*i*_ are the growth curve parameters for individual *i*, t_*ij*_ is day* j* for individual *i* and ${\varepsilon }_{ij}$ is residual error. For every individual, we quantified lnvar_weight_ as the natural logarithm of the variance in the daily differences between observed weights versus expected weights via Gompertz modeling (calculated with *ln* function in R), as well as skewness (skew_weight_; calculated with *skewness* function) and the lag-one autocorrelation (lag1_weight_; calculated with *acf* function) (Fig. 5), following Berghof et al. [1].

**Table 2:** Trait definition for the resilience traits

Resilience trait	Definition
lnvar_weight_	The natural logarithm of the variance of pigs’ daily differences between observed weights versus expected weights via Gompertz modeling of weight versus age (example shown in Fig. 5). A higher value indicates more deviations and, hence, a lower resilience
lnMSE_weight_	The natural logarithm of the mean squared error (equivalent to variance) of pigs’ daily differences between observed weights versus expected weights via linear modeling of weight versus age. A higher value indicates more deviations and, hence, a lower resilience
lnvar_weight_standardized_	The natural logarithm of the variance of a pigs’ standardized weights versus age (mean is zero, standard deviation is one; Fig. 4b, Fig. 5c and g). A higher value indicates more deviations and, hence, a lower resilience
Skew_weight_	The skewness of pigs’ daily differences between observed weights versus expected weights via Gompertz modeling of weight versus age
Lag1_weight_	The lag1 autocorrelation of pigs’ daily differences between observed weights versus expected weights via Gompertz modeling of weight versus age
Straightness	The straightness index, estimated after trajectory analysis of a pigs’ observed weight versus age. Straightness index is estimated as the Euclidean distance between start and end point divided by the total path length covered by the weight trajectory. Maximum value is one (straight line), minimum value is zero (infinite body weight deviations). A lower value indicates more deviations and, hence, a lower resilience
Mean speed	The Mean speed, estimated after trajectory analysis of a pigs’ observed weight versus age. Mean speed is estimated as the total path length covered by the weight trajectory divided by the age difference (d) between end and start. A higher value indicates more deviations and, hence, a lower resilience
lnMSE_FI_	The natural logarithm of the mean squared error (equivalent to variance) of pigs’ daily differences between observed feed intake versus expected feed intake via linear modeling of feed intake versus age. A higher value indicates more deviations and, hence, a lower resilience
lnMSE_dur_	The natural logarithm of the mean squared error (equivalent to variance) of pigs’ daily differences between observed visit duration versus expected visit duration via linear modeling of visit duration versus age. A higher value indicates more deviations and, hence, a lower resilience
lnMSE_n_visit_	The natural logarithm of the mean squared error (equivalent to variance) of pigs’ daily differences between observed number of visits versus expected number of visits via linear modeling of number of visits versus age. A higher value indicates more deviations and, hence, a lower resilience
QR_FI_	The number of off-feed days, calculated as the number of days during which feed intake was in the 5% lowest quantile using quantile regression on age over all pigs. A higher value indicates more off-feed days and, hence, a lower resilience
QR_dur_	The number of off-feed days, calculated as the number of days during which visit duration was in the 5% lowest quantile using quantile regression on age over all pigs. A higher value indicates more off-feed days and, hence, a lower resilience

Next, similar to Putz et al. [16], a linear regression of weight on age was used to estimate the root mean squared error (RMSE) of observed versus expected body weight deviations (calculated with *lm* function in R). As growing pigs (95–155 d) are more or less in a linear phase of their growth curve [30], this linear approach seems justified (Fig. 5a). Hereafter, we calculated the natural logarithm of the MSE (lnMSE_weight_). Here, we used MSE instead of RMSE to make lnMSE_weight_ equivalent to lnvar_weight_.

A major challenge with modelling has to do with some circularity: expected weights are also estimated based on the observed weights, and these “expectations” might come from a biased curve [1, 22]. To circumvent this issue, two different approaches were used. First, following Berghof et al. [24], all weights were standardized by age with a mean of zero and a standard deviation of one for each single day of age (Fig. 4b, Fig. 5c and g). From these standardized weights per day, the natural logarithm of the variance was then calculated (lnvar_weight_standardized_). Pigs with a high lnvar_weight_standardized_ hence showed great variations in weight over time, compared to the population mean. Second, additional deviation traits were derived from trajectory analysis using the *trajr* package in R [31] (Fig. 5d and h). Trajectory analysis can be used to estimate deviations from expected patterns. Here, we estimated mean speed (*TrajDerivatives* function) and the straightness (*TrajStraightness* function). The *trajr* package estimates mean speed as:

$$Mean\ speed=\frac{Total\ path\ length\ of\ weight\ trajectory}{Age\ difference\ between\ start\ and\ end\ point}$$

whereas straightness index was estimated as:

$$straightness =\frac{Euclidean\ distance\ between\ start\ and\ end\ point}{Total\ path\ length\ of\ weight\ trajectory}$$

Hence, an animal with more body weight deviations will have a higher mean speed and a lower straightness index, as the total path length of weight trajectory will increase due to more deviations. The maximum straightness index value is 1, with values below one indicating more deviations from a straight line. The straightness index and mean speed are related, but can differ due to different ADG between animals. For example, two animals with a straightness index of 1 might still differ in mean speed, as a faster growing animal will have a higher mean speed as it will have more’distance traveled’ over the same time.

**Fig. 6:**
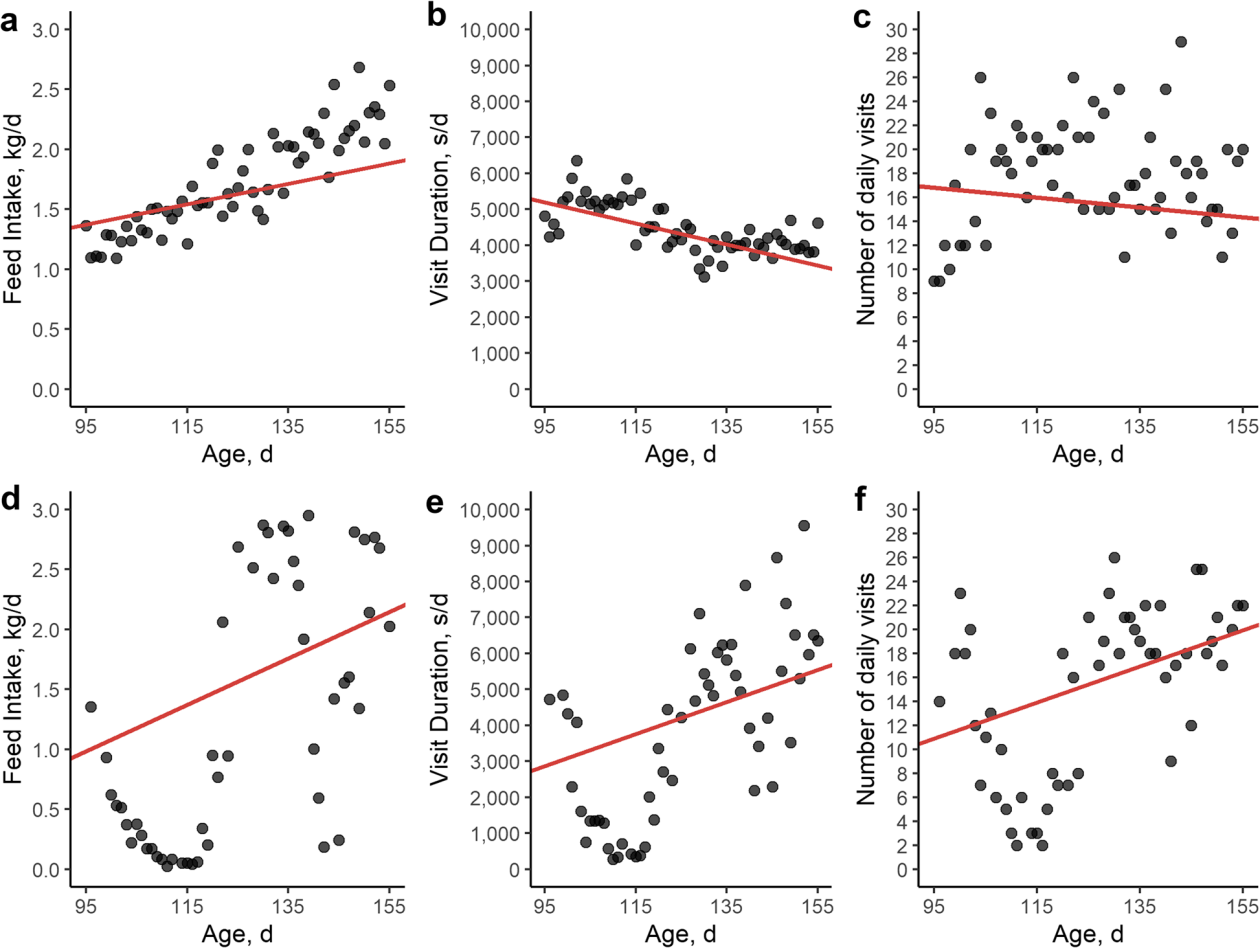
Example of feed intake, visit duration and number of daily visits for two pigs (**a–c** versus **d–f**). These examples are the same animals as shown in Fig. 5, and were selected based on body weight deviations, where the upper pig showed little deviations in observed versus expected body weight, whereas the lower pig showed many deviations in observed versus expected body weight. Red lines indicates the regression line from linear modeling. **a** and **d** Evolution of feed intake (kg/d) versus age (d). Based on the linear regression, lnMSE_FI_ was quantified. **b** and **e** Evolution of visit duration (s/d) versus age in d. Based on the linear regression, lnMSE_dur_ was quantified. **c** and **f** Evolution of number of daily visits to feeder versus age (d). Based on the linear regression, lnMSE_n_visit_ was quantified

For daily feed intake, visit duration and number of visits, the natural logarithm of MSE after linear modeling was calculated using the same methodology as for lnMSE_weight_, respectively leading to the traits lnMSE_FI_, lnMSE_dur_ and lnMSE_n_visit_ (Fig. 6). Moreover, following Putz et al. [16], the number of off-feed days was calculated as the number of days during which feed intake (QR_FI_) and/or visit duration (QR_dur_) was in the 5% lowest quantile using quantile regression (QR) on age over all pigs (Additional file 2: Fig. S2).

Finally, after estimating these traits per pig from the daily AFS recordings, estimates deviating by more than four standard deviations from the mean were set to missing (184 for *A*; 55 for *B*; 30 for *k*; 27 for FI; 35 for ADG; 73 for FCR; 2 for lag1_weight_; 11 for skew_weight_; 0 for lnvar_weight_; 1 for lnMSE_weight_; 2 for lnvar_weight_standardized_; 0 for straightness; 7 for mean speed; 2 for lnMSE_FI_; 1 for lnMSE_dur_; 0 for lnMSE_n_visit_).

### Genetic modelling

The *blupf90* suite of programs [32] was used to estimate genetic parameters. Heritability (*h*^2^) was estimated as the proportion of additive genetic variance divided by total variance. Likewise, the common environmental effect (*c*^2^) was estimated as the proportion of variance explained by random contemporary group effects (*c*), divided by total variance. For the resilience traits, the genetic coefficient of variation (GCV) was estimated as a measure of evolvability or possible selection response of a trait [33]. For the lnvar and lnMSE traits, GCV was estimated as: $GCV=\sqrt{{\sigma }_{a}^{2}}$, as described by [18, 34, 35] for an exponential model.

All single trait animal models were of the form:

$${\varvec{y}}={\varvec{X}}{\varvec{b}}+{\varvec{Z}}{\varvec{a}}+{\varvec{W}}{\varvec{c}}+{\varvec{e}}$$

where ***y*** is the vector with phenotypes for the studied trait; ***b*** is the vector containing the fixed effects (sex, 2 levels; farm, 2 levels) and covariates (maximum age); ***a*** is the vector of additive genetic effects (9,371 animals in pedigree, 6,723 with genotype information), which is assumed to follow a normal distribution for the pedigree matrix (***A***) using only pedigree relationships:

$${\varvec{a}}\sim N(0,{{\varvec{A}}\sigma }_{a}^{2})$$

Or a normal distribution for the ***H*** matrix, combining both pedigree (***A***) and genomic (***G***) relationship matrices following [36–38] using single-step genomic evaluation:

$${\varvec{a}}\sim N(0,{{\varvec{H}}\sigma }_{a}^{2})$$

***c*** is the vector of contemporary group effects (113 levels), assumed to follow a normal distribution $c\sim N(0,{{\varvec{I}}\sigma }_{c}^{2})$, with ***I*** the identity matrix; ***e*** is the vector of residual effects, assumed to follow a normal distribution $e\sim N(0,{{\varvec{I}}\sigma }_{e}^{2})$; ***X***, ***Z*** and ***W*** are incidence matrices for respectively fixed effects, random animal effects and random contemporary group effects. The random contemporary group effect ***c*** is a combination of farm, compartment and date of entrance at farm. Contemporary groups with less than ten pigs were combined in a remainder group (165 pigs).

Likewise, genetic correlations (*r*_g_) between traits were estimated via bivariate animal models of the form:

$$\left[\begin{array}{c}{\varvec{y}}1\\ {\varvec{y}}2\end{array}\right]=\left[\begin{array}{cc}{\varvec{X}}1& 0\\ 0& {\varvec{X}}2\end{array}\right]\left[\begin{array}{c}{\varvec{b}}1\\ {\varvec{b}}2\end{array}\right]+\left[\begin{array}{cc}{\varvec{Z}}1& 0\\ 0& {\varvec{Z}}2\end{array}\right]\left[\begin{array}{c}{\varvec{a}}1\\ {\varvec{a}}2\end{array}\right]+\left[\begin{array}{cc}{\varvec{W}}1& 0\\ 0& {\varvec{W}}2\end{array}\right]\left[\begin{array}{c}{\varvec{c}}1\\ {\varvec{c}}2\end{array}\right]+\left[\begin{array}{c}{\varvec{e}}1\\ {\varvec{e}}2\end{array}\right]$$

Similar to the single-trait animal model, ***y1*** and ***y2*** are the vectors with phenotypes for the studied traits; ***b1*** and ***b2*** are the vectors containing the fixed effects and covariates; ***a1*** and ***a2*** are the vectors of additive genetic effects, which is assumed to follow a normal distribution for the ***H*** matrix using single-step genomic evaluation:

$$\left[\begin{array}{c}{\varvec{a}}1\\ {\varvec{a}}2\end{array}\right]\sim N(\left[\begin{array}{c}0\\ 0\end{array}\right],\left[\begin{array}{cc}{\sigma }_{a1}^{2}& {\sigma }_{a1,a2}\\ {\sigma }_{a1,a2}& {\sigma }_{a2}^{2}\end{array}\right]\otimes {\varvec{H}})$$

***c1*** and ***c2*** are the vectors of contemporary group effects (113 levels), assumed to follow a normal distribution $$\left[\begin{array}{c}{\varvec{c}}1\\ {\varvec{c}}2\end{array}\right]\sim N(\left[\begin{array}{c}0\\ 0\end{array}\right],\left[\begin{array}{cc}{\sigma }_{c1}^{2}& {\sigma }_{c1,c2}\\ {\sigma }_{c1,c2}& {\sigma }_{c2}^{2}\end{array}\right]\otimes {\varvec{I}})$$; ***e1*** and ***e2*** are the vector of residual effects, assumed to be independently normal distributed $$\left[\begin{array}{c}{\varvec{e}}1\\ {\varvec{e}}2\end{array}\right]\sim N(\left[\begin{array}{c}0\\ 0\end{array}\right],\left[\begin{array}{cc}{\sigma }_{e1}^{2}& 0\\ 0& {\sigma }_{e2}^{2}\end{array}\right])$$; ***X1***, ***X2***, ***Z1***, ***Z2***, ***W1*** and ***W2*** are incidence matrices for respectively fixed effects, random animal effects and random contemporary group effects.

### Cross validation

Cross validation was performed using three data masking strategies: within family masking, across family masking and temporal masking. For within and across family masking, we decided to use 5-fold cross-validation with random masking of 20% of the data (based on [39]), resulting in validation datasets of ~ 1,200 pigs. Moreover, ten replications were used to avoid random sampling effects [39], resulting in 50 models (10 × 5-fold validation) per trait. For within family masking strategy, one out of five offspring was randomly masked per sire. In our dataset, every sire had a mean of 45.7 offspring (range = 1–182, SD = 42.9), leading to a mean of 9 masked offspring per sire. For the across family masking strategy, all progeny from one out of five sires was randomly masked. As a result, the within-family strategy is valuable to estimate predictive ability from close relationships, whereas across-family masking allows to estimate predictive ability of distant relationships [39]. For the temporal cross-validation strategy, animals born after 01-10-2020 were masked (~ 30% of dataset). Temporal cross-validation allows to estimate forward (future) predictive ability.

The process of cross-validation was as follows. First, a univariate animal model (as specified before) was used on the full dataset using the *remlf90* software. Observed phenotypes were adjusted for fixed and non-genetic random effects based on these results using the *predictf90* software:

$${{\varvec{y}}}^{*}={\varvec{y}}-(\widehat{{\varvec{b}}}+\widehat{{\varvec{c}}} )=\widehat{{\varvec{a}}}+\widehat{{\varvec{e}}}$$

Predictive abilities were estimated as the Pearson correlation between breeding values of a validation dataset (with masked phenotypes) and the adjusted phenotypes ($y^\ast$ ):

$$Predictive\ ability=r({EBV}_{masked},{y}^{*})$$

Next, predictive abilities were expressed as a cross validation accuracy. This was done by dividing the predictive ability by the square root of the estimated *h*^2^:

$$Predictive\ ability\ accuracy=\frac{r\left({EBV}_{masked},{y}^{*}\right)}{h}$$

### Evaluating the impact of observation frequency and observation period

**Fig. 7:**
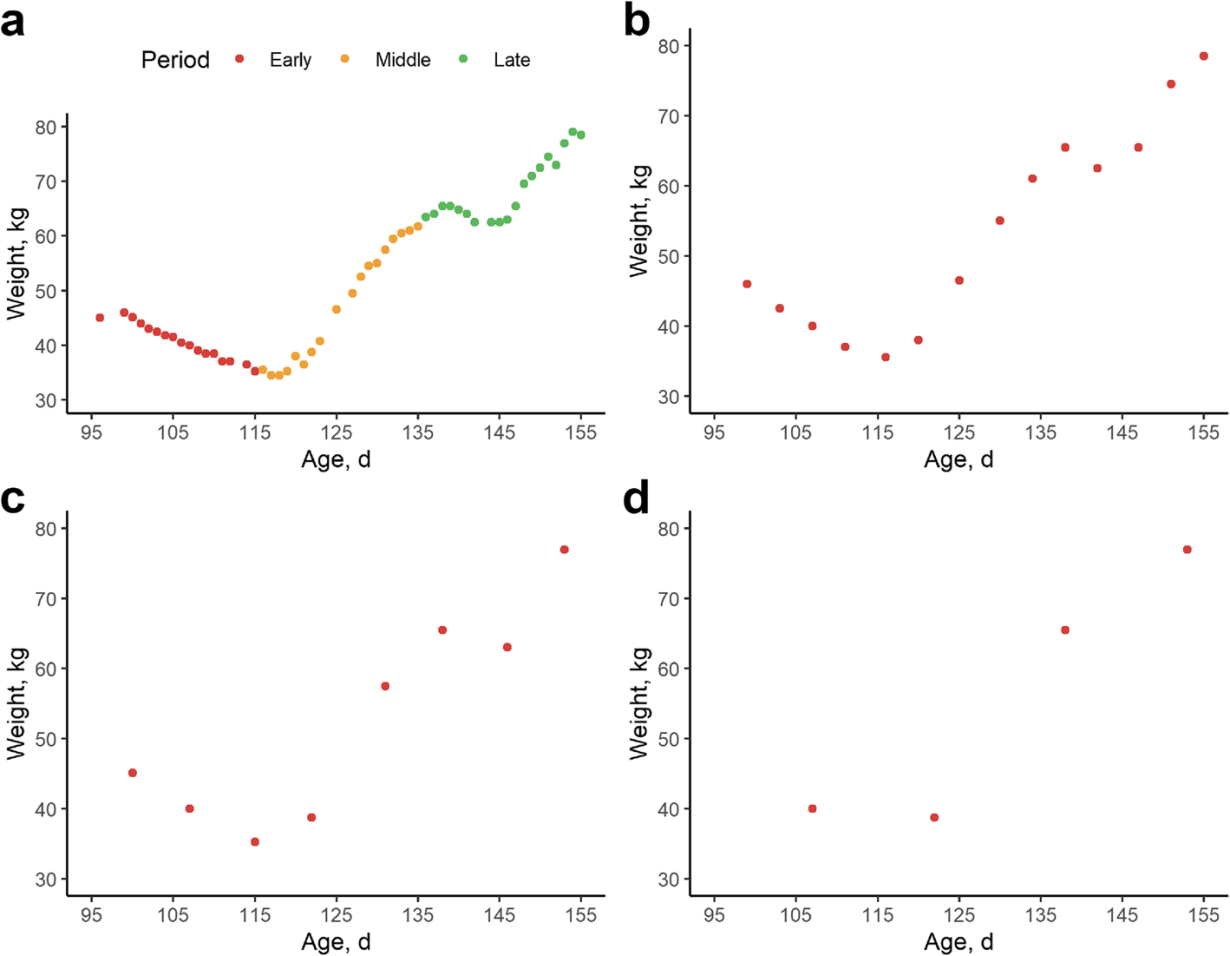
Example of different observation frequency and observation period settings for an individual pigs’ weight data. **a** All daily weight records within the 95–155 days of age interval, colored per observation period 95–115 d (early, red), 115–135 d (middle, orange), 135–155 d (late, green). **b** A subset sampled from the full dataset with only 1 out of 4 data points, which corresponds to about two records per week. **c** A subset sampled from the full dataset with only 1 out of 7 data points, which corresponds to about one record per week. **d** A subset sampled from the full dataset with only 1 out of 14 data points, which corresponds to about one record every two weeks

Finally, the impact of observation frequency and observation period were evaluated. First, the influence of observation frequency on parameter stability was evaluated. Based on the full dataset, subsets were made with 1 out of 4 records per animal (~ 2 records per week), 1 out of 7 records per animal (1 record per week) and 1 out of 14 records per animal (1 record every two weeks) (Fig. 7). Phenotypic and genetic correlations were estimated for the full model versus reduced datasets using bivariate animal models. These (genetic) correlations indicate to what extent traits are sensitive to changes in observation frequency. Second, to assess influence of observation period, the full 60-day dataset with all records was divided in three age groups of twenty days: (i) 95–115 days of age (early), (ii) 115–135 days of age (middle) and (iii) 135–155 days of age (late). Based on these subsets, all traits were recalculated leading to, for example, lnvar_weight-early_, lnvar_weight-middle_ and lnvar_weight-late_. Hereafter, bivariate animal models were run within each trait to estimate phenotypic and genetic correlations between periods. These (genetic) correlations indicate the repeatability of a trait and whether a given trait genetically shifts over time.

## Results

An overview of the main trait distributions and their estimated *h*^2^ and variance components is given in Table 3. All estimated phenotypic and genetic correlations are given in Table 4. Heritabilities for Gompertz growth curve parameters *A*, *B* and *k* were low (6.8%–10.3%). The body weight deviation traits skew_weight_ (2.9%) and lag1_weight_ (6.2%) were also lowly heritable. Standardizing weights before estimating lnvar increased *h*^2^ (12.1% for lnvar_weight_standardized_ versus 11.0% for lnvar_weight_). However, *h*^2^ estimates for body weight deviations were highest for trajectory parameters straightness (15.5%) and mean speed (20.2%), which were moderately heritable. Deviations related to feed intake and feeding behaviour had higher *h*^2^ (20.7%–28.3%) than body weight deviations (8.9%–20.2%). Despite low to moderate *h*^2^, the resilience trait indicators had high genetic coefficients of variation: 20.5%–30.2% for body weight deviations and 29.1%–33.4% for feed intake and feeding behaviour deviations. QR_FI_ (*h*^2^ = 9.4%) and QR_dur_ (*h*^2^ = 16.1%) had low to moderate *h*^2^ estimates.

**Table 3:** Descriptive statistics and genetic parameters

Trait	Mean (sd)	Range	*h*^2^ (se)	*c*^2^ (se)	σ_a_	σ_c_	σ_e_
Weight_start_, kg	46.5 (7.5)	15.5–83.0	-	-	-	-	-
Weight_end_, kg	108.9 (12.2)	42.5–156.8	-	-	-	-	-
ADG, g/d	1,038 (155)	−16–1,840	16.5 (2.6)	33.6 (3.4)	67	96	117
AFI, g/d	2,326 (319)	1,006–3,619	33.8 (3.5)	22.0 (2.9)	201	162	229
FCR, g/g	2,240 (240)	1,380–3,200	22.9 (3.3)	21.8 (2.9)	111	108	172
*A*	243.4 (124.4)	36.2–945.5	6.8 (2.3)	24.7 (2.9)	33.5	63.9	106.5
*B*	6.75 (2.75)	2.73–28.27	8.9 (1.8)	15.4 (2.1)	0.81	1.07	2.36
*k* × 1,000	14.8 (6.0)	2.2–46.0	10.3 (2.3)	23.9 (2.9)	1.9	2.9	4.8
Fat depth, mm	9.4 (1.5)	4.8–17.4	52.7 (4.2)	5.6 (1.2)	1.2	0.4	1.1
Muscle depth, mm	82.6 (6.5)	59.3–105.1	36.6 (3.8)	10.9 (1.9)	3.9	2.1	4.7
lnvar_weight_	0.85 (0.72)	−2.01–3.61	11.0 (2.8)	24.3 (2.9)	0.22	0.32	0.52
lnMSE_weight_	1.08 (0.73)	−1.97–3.94	8.9 (2.5)	23.3 (2.8)	0.21	0.33	0.57
lnvar_weight_standardized_	−2.86 (0.85)	−5.67–0.45	12.1 (2.8)	17.0 (2.4)	0.30	0.36	0.73
Skew_weight_	−0.29 (0.44)	−2.08–1.48	2.9 (0.9)	10.4 (1.7)	0.08	0.14	0.41
Lag1_weight_	0.56 (0.17)	−0.05–0.97	6.2 (1.8)	19.2 (2.5)	0.04	0.08	0.15
Straightness	0.80 (0.08)	0.57–0.97	15.5 (2.9)	21.6 (2.7)	0.03	0.03	0.05
Mean speed	1.85 (0.19)	1.24–2.51	20.2 (3.2)	15.2 (2.2)	0.07	0.06	0.13
lnMSE_FI_	−1.16 (0.69)	−3.71–0.48	23.3 (3.4)	19.8 (2.6)	0.29	0.27	0.46
lnMSE_dur_	13.42 (0.64)	11.24–15.91	28.3 (3.3)	16.1 (2.3)	0.33	0.25	0.47
lnMSE_n_visit_	1.90 (0.68)	−0.78–4.25	20.7 (3.5)	17.9 (2.3)	0.33	0.30	0.56
QR_FI_, d	2.30 (2.67)	0.00–15.00	9.4 (2.1)	20.9 (2.7)	0.82	1.22	2.23
QR_dur_, d	2.28 (2.99)	0.00–15.00	16.2 (3.0)	18.0 (2.5)	1.2	1.3	2.4

Parameters were estimated from pigs of 95–155 days of age. Heritability (*h*^2^) and common environmental effect (*c*^2^) estimates are given in percentages. Genetic parameters were estimated via single-step genomic evaluation, integrating both pedigree and genomic relationships. Additive genetic standard deviation (σ_a_), common environmental standard deviation (σ_c_) and residuals standard deviation (σ_e_) are given in trait units. Estimates of the *k*-parameter of Gompertz modeling are multiplied by a factor 1,000 (*k* × 1,000). ADG: average daily gain; AFI: average feed intake; FCR: feed conversion ratio; *A*, *B* and *k*: Gompertz growth curve parameters; lnvar_weight_: natural logarithm of variance of observed versus predicted weights; lnMSE_weight_: natural logarithm of mean squared error of weight in function of age; lnvar_weight_standardized_: natural logarithm of variance of standardized weights; skew_weight_: skewness of observed versus predicted weight distribution; lag1_weight_: lag1 autocorrelation of observed versus predicted weight distribution; straightness: straightness index of weight in function of age after trajectory analysis; mean speed: mean speed of weight in function of age after trajectory analysis; lnMSE_FI_: natural logarithm of mean squared error of feed intake in function of age; lnMSE_dur_: natural logarithm of mean squared error of visit duration in function of age; lnMSE_n_visit_: natural logarithm of mean squared error of number of daily visits in function of age; QR_FI_: number of days with feed intake below 5% of quantile after quantile regression; QR_dur_: number of days with visit duration below 5% of quantile after quantile regression

**Table 4:** Correlation table with phenotypical (below diagonal) and genetic correlations (above diagonal)

Trait	ADG	AFI	FCR	*A*	*B*	*k*	Fat	Muscle	lnvar_wg_	lnMSE_wg_	lnvar_st_	Skew_wg_	Lag1_wg_	Straight	Speed	lnMSE_FI_	lnMSE_d_	lnMSE_n_	QR_FI_	QR_dur_
ADG		0.79	0.01	0.35	0.4	0.15	0.05	−0.18	0.05	0.09	0.19	0.08	−0.05	0.38	0.50	0.15	−0.08	0.12	−0.70	−0.48
AFI	0.73		0.61	−0.06	0.35	0.45	0.18	−0.10	0.26	0.31	0.34	0.38	−0.09	0.01	0.67	0.44	−0.04	0.06	−0.58	−0.18
FCR	−0.39	0.3		−0.54	0.1	0.52	0.23	0.05	0.37	0.39	0.33	0.30	0.02	−0.41	0.41	0.49	0.03	−0.09	−0.02	0.30
*A*	0.19	0.04	−0.2		−0.46	-0.83	0.01	−0.17	−0.34	−0.27	−0.13	0.04	−0.04	0.53	−0.21	−0.44	−0.18	0.32	−0.34	−0.34
*B*	0.01	0.03	0.03	−0.41		0.76	0.15	0.04	0.05	0.18	0.32	0.31	0.1	0.06	0.24	0.27	0.14	−0.25	−0.04	−0.02
*k*	−0.08	0.08	0.22	−0.71	0.82		0.05	0.14	0.32	0.38	0.38	0.19	0.03	−0.34	0.46	0.50	0.13	−0.26	0.09	0.23
Fat	0.12	0.16	0.05	0.04	0.04	−0.02		0.02	−0.10	−0.08	−0.03	0.18	0.03	0.15	−0.07	−0.04	0.00	0.08	−0.13	−0.07
Muscle	0.14	0.16	0.01	−0.09	0.02	0.09	0.08		−0.02	−0.03	−0.10	0.16	0.01	−0.04	−0.07	−0.02	−0.02	0.08	0.05	0.01
lnvar_wg_	−0.15	−0.03	0.17	−0.07	0.04	0.07	−0.06	0.15		0.93	0.74	0.32	0.53	−0.77	0.74	0.78	0.36	−0.22	0.53	0.49
lnMSE_wg_	−0.19	−0.05	0.21	0.04	0.21	0.13	−0.05	0.1	0.88		0.80	0.45	0.56	−0.72	0.76	0.76	0.33	−0.18	0.54	0.51
lnvar_st_	−0.14	−0.05	0.13	−0.05	0.2	0.16	0.01	0.06	0.58	0.61		0.15	0.61	−0.36	0.55	0.55	0.28	−0.07	0.32	0.39
Skew_wg_	−0.15	−0.08	0.1	0.04	0.01	0	−0.02	−0.08	0.01	0.08	0.07		−0.10	−0.37	0.46	0.28	0.05	0.01	0.08	0.07
Lag1_wg_	−0.22	−0.15	0.11	0.08	−0.03	−0.08	−0.01	−0.02	0.64	0.6	0.42	0.1		−0.04	0.00	0.16	0.11	0.04	0.42	0.29
Straight	0.32	0.16	−0.24	0.21	−0.08	−0.19	0.11	−0.15	−0.73	−0.65	−0.38	0	−0.09		−0.62	−0.73	−0.37	0.31	−0.67	−0.62
Speed	0.35	0.33	0.01	−0.06	0.08	0.12	−0.03	0.21	0.62	0.52	0.31	−0.07	−0.03	−0.75		0.80	0.30	−0.21	0.09	0.22
lnMSE_FI_	−0.03	0.11	0.2	−0.15	0.08	0.16	−0.08	0.18	0.69	0.6	0.36	−0.01	0.2	−0.71	0.69		0.49	−0.34	0.44	0.51
lnMSE_d_	−0.21	−0.18	0.04	−0.05	0.03	0.02	-0.05	0.04	0.49	0.43	0.28	0.01	0.19	−0.53	0.39	0.69		0.00	0.43	−0.12
lnMSE_n_	−0.16	−0.1	0.07	0.09	−0.06	−0.12	0.01	−0.11	0.04	0.08	0.05	0.07	0.07	−0.05	−0.05	0.03	0.16		−0.18	−0.37
QR_FI_	−0.51	−0.53	0.01	−0.09	0.02	0.03	−0.12	−0.01	0.5	0.48	0.37	0.07	0.29	−0.56	0.23	0.55	0.53	0.13		0.72
QR_dur_	−0.37	−0.31	0.11	−0.09	0	0.05	-0.09	0.04	0.49	0.47	0.34	0.07	0.26	−0.54	0.3	0.57	0.37	0.05	0.72	

Standard errors of genetic correlations are given in Additional file 3: Table S1. Genetic parameters were estimated via single-step genomic evaluation, integrating both pedigree and genomic relationships. ADG: average daily gain; AFI: average feed intake; FCR: feed conversion ratio; *A*, *B* and *k*: Gompertz growth curve parameters; Fat: fat depth; Muscle: muscle depth; lnvar_wg_: natural logarithm of variance of observed versus predicted weights; lnMSE_wg_: natural logarithm of mean squared error of weight in function of age; lnvar_st_: natural logarithm of variance of standardized weights; skew_wg_: skewness of observed versus predicted weight distribution; lag1_wg_: lag1 autocorrelation of observed versus predicted weight distribution; straight: straightness index of weight in function of age after trajectory analysis; speed: mean speed of weight in function of age after trajectory analysis; lnMSE_FI_: natural logarithm of mean squared error of feed intake in function of age; lnMSE_d_: natural logarithm of mean squared error of visit duration in function of age; lnMSE_n_: natural logarithm of mean squared error of number of daily visits in function of age; QR_FI_: number of days with feed intake below 5% of quantile after quantile regression; QR_dur_: number of days with visit duration below 5% of quantile after quantile regression

Phenotypic and genetic correlations (Table 4) between lnvar_weight_ and most other body weight deviation traits were high (*r*_p_ = 0.58–0.88; *r*_g_ = 0.53–0.93), except for skew_weight_ (*r*_p_ = 0.01; *r*_g_ = 0.32). Furthermore, lnvar_weight_ was phenotypically and genetically also moderately to highly correlated with deviations in feeding duration (lnMSE_dur_; *r*_p_ = 0.49; *r*_g_ = 0.36) and feed intake (lnMSE_FI_; *r*_p_ = 0.69; *r*_g_ = 0.78). Deviations in feed intake (lnMSE_FI_) were moderately correlated with deviations in feeding duration (lnMSE_dur_; *r*_p_ = 0.69, *r*_g_ = 0.49) but lowly correlated with deviations in number of daily visits (lnMSE_n_vis_; *r*_p_ = 0.16, *r*_g_ = −0.34). ADG was moderately correlated with straightness (*r*_p_ = 0.32; *r*_g_ = 0.38) and mean speed (*r*_p_ = 0.32; *r*_g_ = 0.50). Additionally, ADG was negatively correlated with the number of days with a very low feed intake (QR_FI_; *r*_p_ = −0.51, *r*_g_ = −0.70), indicating that pigs with high ADG have less off-feed days. A similar pattern was observed for AFI. For FCR, a low to moderate favourable correlation was found with lnvar_weight_ (*r*_p_ = 0.17; *r*_g_ = 0.37) and lnMSE_FI_ (*r*_p_ = 0.20; *r*_g_ = 0.49), indicating that pigs with more deviations in weight and feed intake have a higher FCR.

An overview of estimated predictive abilities per trait using both pedigree relationships and single-step genomic evaluation for three cross-validation strategies is given in Table 5 as cross validation accuracy and in Additional file 4: Table S2 as correlation.

**Table 5:** Predictive ability accuracy for cross validation scenarios: masking across or within family and temporal masking

Trait	Across Family		Within Family		Temporal	
	**BLUP**	**ssGBLUP**	**BLUP**	**ssGBLUP**	**BLUP**	**ssGBLUP**
ADG	0.22	0.42	0.54	0.62	0.27	0.42
AFI	0.22	0.52	0.45	0.64	0.26	0.57
FCR	0.38	0.56	0.61	0.69	0.31	0.50
* A *	0.12	0.19	0.23	0.27	0.12	0.27
* B *	0.13	0.30	0.37	0.47	0.17	0.44
* K *	0.22	0.37	0.50	0.56	0.19	0.41
Fat	0.36	0.62	0.55	0.69	0.21	0.45
Muscle	0.31	0.58	0.51	0.66	0.31	0.55
lnvar_weight_	0.27	0.36	0.60	0.57	0.39	0.42
lnMSE_weight_	0.27	0.34	0.60	0.57	0.40	0.44
lnvar_weight_standardized_	0.20	0.34	0.49	0.52	0.26	0.34
Skew_weight_	0.00	0.12	0.23	0.23	0.12	0.23
Lag1_weight_	0.20	0.28	0.40	0.44	0.12	0.28
Straightness	0.25	0.38	0.58	0.58	0.36	0.48
Mean speed	0.29	0.40	0.53	0.60	0.31	0.49
lnMSE_FI_	0.33	0.52	0.62	0.68	0.41	0.58
lnMSE_dur_	0.39	0.60	0.56	0.68	0.53	0.71
lnMSE_n_visit_	0.37	0.53	0.59	0.64	0.42	0.53
QR_FI_	0.26	0.36	0.46	0.52	0.46	0.49
QR_dur_	0.32	0.47	0.52	0.60	0.45	0.60

Predictive ability accuracy was estimated by dividing the predictive ability correlation by the square root of the estimated heritability. These predictive abilities as a correlation and standard deviation of these estimates are provided in Additional file 4: Table S2. For temporal masking, there was only one estimate, and hence, no standard deviation was calculated. BLUP: Best linear unbiased prediction. Genetic parameters estimated with pedigree relationships; ssGBLUP: single-step genomic BLUP: genetic parameters estimated with single-step genomic evaluation. ADG: average daily gain; AFI: average feed intake; FCR: feed conversion ratio; *A*, *B* and *k*: Gompertz growth curve parameters; lnvar_weight_: natural logarithm of variance of observed versus predicted weights; lnMSE_weight_: natural logarithm of mean squared error of weight in function of age; lnvar_weight_standardized_: natural logarithm of variance of standardized weights; skew_weight_: skewness of observed versus predicted weight distribution; lag1_weight_: lag1 autocorrelation of observed versus predicted weight distribution; straightness: straightness index of weight in function of age after trajectory analysis; mean speed: mean speed of weight in function of age after trajectory analysis; lnMSE_FI_: natural logarithm of mean squared error of feed intake in function of age; lnMSE_dur_: natural logarithm of mean squared error of visit duration in function of age; lnMSE_n_visit_: natural logarithm of mean squared error of number of daily visits in function of age; QR_FI_: number of days with feed intake below 5% of quantile after quantile regression; QR_dur_: number of days with visit duration below 5% of quantile after quantile regression

Predictive ability accuracies of skew_weight_ were low for all strategies (0.00–0.23). For the body weight deviation traits, the trajectory parameters mean speed and straightness showed the highest predictive ability accuracies with single-step genomic evaluation (0.38–0.60). Feed intake deviations showed higher predictive ability accuracies than body weight deviations, and single-step genomic evaluation seemed to relatively increase predictive abilities for feed intake deviations more. Predictive abilities for lnMSE_dur_, for example, increased by 54%, 21% and 33% respectively when adding genomics to across, within and temporal masking strategy.

Phenotypic correlations per trait for different observation frequencies are given as pairwise correlation plots in Additional file 5: Fig. S3. An overview of genetic correlations within traits estimated by using different observation frequencies ranging from 1 in 4 to 1 in 14 is provided in Table 6. As expected, ADG does not change substantially with lower data density (*r*_p_ = 0.85 and *r*_g_ = 0.95 with 1 in 14 density), as it is estimated as the average gain over a long period. FCR fluctuates more with lower observation frequency: when considering only 1 in 14 data points, the phenotypic and genetic correlations with the full dataset drop (*r*_p_ = 0.45 and *r*_g_ = 0.76) and *h*^2^ drops from 22.1% to 10.1%. For the resilience traits, lnvar and lnMSE estimates were least dependent on observation frequency with *r*_p_ = 0.44–0.76 and *r*_g_ = 0.79–0.96 in the most extreme scenario, although the *h*^2^ estimates decreased substantially from *h*^2^ = 10.6%–23.3% to *h*^2^ = 5.1%–10.1%. Skew_weight_ and lag1_weight_ were strongly impacted by differences in observation frequency, with *r*_p_ = 0.05–0.08 and *r*_g_ = 0.02–0.14 in the most extreme scenario. The trajectory parameters mean speed and straightness were moderately affected by data density (*r*_p_ = 0.29–0.33 and *r*_g_ = 0.50 with one in 14 data points), but showed a smaller decrease in *h*^2^ estimate from *h*^2^ = 15.0%–21.4% to *h*^2^ = 13.1%–17.7%.

**Table 6:** Genetic parameters of full dataset versus reduced datasets (1 in x data points)

Trait (full dataset)	1 in 4		1 in 7		1 in 14	
	***h***^**2**^ **(se)**	***r***_**g**_ **(se)**	***h***^**2**^ **(se)**	***r***_**g**_ **(se)**	***h***^**2**^ **(se)**	***r***_**g**_ **(se)**
ADG	17.0 (2.8)	0.99 (0.00)	17.0 (2.9)	0.98 (0.00)	17.1 (2.7)	0.95 (0.01)
AFI	21.5 (2.9)	0.99 (0.00)	19.4 (2.6)	0.97 (0.00)	15.2 (2.2)	0.93 (0.00)
FCR	22.1 (2.9)	0.92 (0.02)	14.0 (2.1)	0.84 (0.01)	10.1 (1.8)	0.76 (0.03)
*A*	11.1 (2.6)	0.92 (0.02)	10.3 (2.4)	0.86 (0.04)	9.1 (2.5)	0.65 (0.07)
*B*	5.7 (1.7)	0.95 (0.03)	4.3 (1.8)	0.90 (0.04)	3.8 (1.3)	0.79 (0.07)
*k*	12.0 (2.4)	0.97 (0.02)	9.6 (2.2)	0.92 (0.03)	9.3 (1.9)	0.87 (0.05)
lnvar_weight_	14.7 (2.7)	0.96 (0.01)	9.2 (1.8)	0.92 (0.02)	5.8 (1.3)	0.79 (0.06)
lnMSE_weight_	10.6 (2.4)	0.97 (0.01)	6.6 (1.6)	0.93 (0.00)	5.1 (1.3)	0.79 (0.02)
lnvar_weight_standardized_	12.9 (2.7)	1.00 (0.00)	10.4 (2.0)	0.99 (0.00)	9.3 (1.9)	0.96 (0.01)
Skew_weight_	3.4 (1.1)	0.66 (0.08)	2.8 (0.8)	0.50 (0.11)	3.5 (0.9)	0.14 (0.13)
Lag1_weight_	5.4 (0.7)	0.47 (0.08)	5.8 (0.8)	0.23 (0.09)	5.5 (0.8)	0.02 (0.09)
Straightness	15.0 (0.8)	0.76 (0.08)	14.0 (0.0)	0.63 (0.06)	13.1 (0.0)	0.50 (0.05)
Mean speed	21.4 (2.9)	0.69 (0.08)	21.2 (3.0)	0.56 (0.09)	17.7 (2.8)	0.50 (0.10)
lnMSE_FI_	21.6 (2.9)	0.97 (0.00)	12.4 (2.1)	0.93 (0.01)	5.5 (1.3)	0.84 (0.09)
lnMSE_dur_	20.6 (2.8)	0.96 (0.01)	13.4 (2.3)	0.95 (0.00)	7.8 (1.8)	0.90 (0.03)
lnMSE_n_visit_	23.3 (3.1)	0.98 (0.00)	18.5 (2.9)	0.97 (0.01)	10.1 (2.2)	0.91 (0.03)
QR_FI_	4.3 (1.1)	0.91 (0.01)	4.1 (1.6)	0.87 (0.06)	2.8 (0.9)	0.62 (0.16)
QR_dur_	9.2 (1.7)	0.94 (0.01)	8.6 (2.0)	0.91 (0.04)	5.1 (1.6)	0.57 (0.05)

Heritability estimates (*h*^2^) of traits and genetic correlation (*r*_g_) estimates between traits estimated on full dataset versus traits estimated on reduced datasets are given. Pairwise Pearson correlation plots for each trait over different observation frequencies are given in Additional file 5: Fig. S3. Genetic parameters were estimated via pedigree evaluation, using only pedigree relationships. ADG: average daily gain; AFI: average feed intake; FCR: feed conversion ratio; *A*, *B* and *k*: Gompertz growth curve parameters; lnvar_weight_: natural logarithm of variance of observed versus predicted weights; lnMSE_weight_: natural logarithm of mean squared error of weight in function of age; lnvar_weight_standardized_: natural logarithm of variance of standardized weights; skew_weight_: skewness of observed versus predicted weight distribution; lag1_weight_: lag1 autocorrelation of observed versus predicted weight distribution; straightness: straightness index of weight in function of age after trajectory analysis; mean speed: mean speed of weight in function of age after trajectory analysis; lnMSE_FI_: natural logarithm of mean squared error of feed intake in function of age; lnMSE_dur_: natural logarithm of mean squared error of visit duration in function of age; lnMSE_n_visit_: natural logarithm of mean squared error of number of daily visits in function of age; QR_FI_: number of days with feed intake below 5% of quantile after quantile regression; QR_dur_: number of days with visit duration below 5% of quantile after quantile regression

The influence of observation period was studied by dividing the full 60-day dataset (95–155 days of age) in three 20-day time periods during the finishing phase (early, middle and late). Phenotypic correlations per trait over time periods are given as pairwise correlation plots in Additional file 7: Fig. S4. Genetic correlations for each time period versus the full dataset within each trait are given in Additional file 6: Table S3. For ADG and FCR, early, middle and late estimates are moderately to highly correlated with the full dataset (respectively *r*_p_ = 0.59–0.63; *r*_g_ = 0.71–0.82 and *r*_p_ = 0.48–0.55; *r*_g_ = 0.65–0.85), although genetic correlations are low to moderate within time periods (respectively *r*_p_ = 0.04–0.17; *r*_g_ = 0.24–0.41 and *r*_p_ = −0.03–0.05; *r*_g_ = 0.34–0.66). This is in contrast to AFI, where correlations were also moderate to high between time periods (*r*_p_ = 0.45–0.68; *r*_g_ = 0.65–0.87). The body weight deviation traits lnvar_weight_, lnMSE_weight_, lnvar_weight_standardized_, straightness and mean speed show high correlations between time periods and the full dataset (*r*_p_ = 0.47–0.74; *r*_g_ = 0.63–0.92) and moderate to high correlations within time periods (*r*_p_ = 0.23–0.50; *r*_g_ = 0.45–0.78). In contrast, lag1_weight_ and skew_weight_ show in general low correlations over time periods (*r*_p_ = −0.02–0.11; *r*_g_ = −0.15–0.35). Feed intake deviations lnMSE_FI_, lnMSE_duration_ and lnMSE_n_visit_ showed moderate to high (genetic) correlations (*r*_p_ = 0.32–0.65; *r*_g_ = 0.73–0.97).

## Discussion

Increasing resilience is becoming priority in modern pig breeding [1, 6]. Therefore, this study investigated resilience traits based on weight, feed intake and feeding behaviour in pigs which were estimated as perturbations in longitudinal data. We demonstrate that these resilience traits are lowly to moderately heritable and have good predictive abilities in cross-validation analyses. Moreover, deviations in individual body weight and feed intake trajectories are genetically highly correlated and show low to moderate favourable genetic correlations with feed conversion ratio. Lastly, we show that the observation frequency and observation period impact some resilience traits more severely than others. lnvar_weight_standardized_ and lnMSE_FI_, for example, were more robust to low observation frequencies (as low as one data point in fourteen days) and showed moderate repeatability over three 20-day time periods of the finishing phase.

In the first part of our study, we quantified and evaluated several resilience traits. The body weight deviation traits lnvar_weight_, skew_weight_, lag1_weight_ were based on [1, 5] after Gompertz growth curve modelling, whereas lnvar_weight_standardized_ was based on Berghof et al. [24] after standardizing weights per age. The main difference between the two lnvar traits is that lnvar_weight_ uses the pigs’ individual data as a reference (based on growth curve modelling), whereas lnvar_weight_standardized_ takes the population statistics as a reference. The deviations in feed intake and behaviour (lnMSE_FI_, lnMSE_dur_, lnMSE_n_visit_, QR_FI_, QR_dur_) were based on Putz et al. [16], although we chose to use MSE instead of RMSE, as this allowed us to directly estimate GCV [18]. In addition to these previously described resilience traits, we deducted resilience traits from linear modelling and trajectory analysis to our weight data in the finishing phase of pigs (lnMSE_weight_, straightness, mean speed). We believe this approach is justified, as an expected weight evolution in the finishing phase of pigs is more or less linear [30]. Our hypothesis is that any deviation from this linear trajectory is probably due to an external challenge which can impact a pigs’ optimal production potential and challenges its resilience. Although trajectory analysis was developed for the analysis of (wild) animals’ actual trajectories in time and space [31], we believe this methodology could be translated to weight patterns of finishing pigs. The start weight of a pig can be regarded as the starting point, following a specific path over time to reach an end weight. Moreover, trajectory analysis is appealing as it does not require any complex modelling of expected (weight) trajectories. The main issue with modelling is that the predicted values tend to follow the observed values, complicating the prediction of the optimal production curve for challenged animals [1, 22]. Figure 5e, for example, shows that the modelled Gompertz growth curve is more or less the mean of the observed values, which results in an overestimation of positive deviations, and an underestimation of negative deviations [1].

To our knowledge, this is the first study to report *h*^2^ for body weight deviation traits lnvar_weight_, lnvar_weight_standardized_ and lnMSE_weight_ in pigs (Table 3). Our *h*^2^ estimates range 8.9%–12.1% for these traits, which is similar to the *h*^2^ estimate of 9%–11% in similar body weight deviations in layer chickens [24]. *h*^2^ and GCV for lnvar_weight_standardized_ were higher (*h*^2^ = 12.1%; GCV = 30.2%) compared to lnvar_weight_ (*h*^2^ = 11.0%; GCV = 21.6%) and lnMSE_weight_ (*h*^2^ = 8.9%; GCV = 20.5%). This might be because lnvar_weight_standardized_ corrects for a scaling effect, since changes in mean levels tend to change variance levels as well [1, 40, 41]. Remarkably, straightness and mean speed had slightly higher *h*^2^ estimates (15.5% and 20.2%), whereas *h*^2^ of lag1_weight_ (2.9%) and skew_weight_ (6.2%) was very low, similar to Poppe et al. [23]. The estimated *h* ^2^ of feed intake deviations (QR_FI,_ lnMSE_FI_; *h*^2^ = 9.4%–23.3%) and feeding behaviour deviations (QR_dur,_ lnMSE_dur_, lnMSE_n_visit_; *h*^2^ = 16.2%–28.3%) were also comparable to previous studies in pigs by Putz et al. [16] (*h*^2^ = 8%–26% for feed intake), Homma et al. [18] (*h*^2^ = 31% for feed intake; *h*^2^ = 36%–40% for feeding behaviour), and Kavlak and Uimari [19] (*h*^2^ = 7%–11% for feed intake; *h*^2^ = 16%–20% for feeding behaviour). Estimated GCV for lnvar_weight_ and lnMSE_weight_ were 21%–22% and were lower than GCV estimates of 29%–33% for lnvar_weight_standardized_, lnMSE_FI_, lnMSE_dur_ and lnMSE_n_visit_, but in the same range as (22%–39%) [18]. These high genetic coefficients of variation indicate a large potential for genetic improvement of these traits [1, 35].

There are no standard guidelines yet on how to perform quality control of weight data from AFS. The quality control procedure in the current paper was based on the structure and identified issues from our dataset, combined with the methodology from previous work [20]. We would like to stress the importance of rigid quality control on an individual level when quantifying resilience traits, especially for body weight deviations. In contrast to feed intake and feeding behaviour, weight is accumulated over time, i.e. you can only gain or lose weight gradually. However, erroneous weights, such as sudden drops and rises, do often appear in raw data from AFS. These errors can be technical (machine error) or due to a learning curve of the pigs after introduction to AFS [20] (Fig. 1). Without any quality control, estimated *h*^2^ for lnvar_weight_, straightness and mean speed were very low (*h*^2^ = 1.8%–4.0%; results not shown). Applying a limited quality control on a population level, for example applying minimum and maximum thresholds for weight as a function of age, increased *h*^2^ estimates to *h*^2^ = 5.7%–7.1% (results not shown). However, these estimates are still considerably lower than what is achieved in a dataset with a rigid, individual quality control. Here, standard guidelines on quality control of AFS data might be valuable, although there might be no “one size fits all” approach. Our advice is to always visually check the weight trajectories of individual animals with outlying resilience traits, for example lnvar_weight_ > 3 standard deviations from mean, even after quality control.

The data in Table 4 suggests a strong connection between resilience traits for feed intake and weight, as shown by the estimated genetic correlation of 0.78 between lnvar_weight_ and lnMSE_FI_. This correlation implies that individual deviations in feed intake are rapidly reflected in weight perturbations. However, the correlation does not equal one, indicating that these various indicators of resilience may signify different aspects of pigs’ resilience. Here, changes in feed intake might be considered as a short term response to a challenge, as a challenged animals’ appetite is usually directly affected [1, 2]. Variations in weight can be considered as a moderate term response since weight gain/loss is mainly determined by food and water intake and several other factors over time. Moreover, we estimated a favourable genetic correlation between lnMSE_weight_ or lnMSE_FI,_ and FCR (*r*_g_ = 0.39–0.49). As feed efficiency is one of the most important traits in pig breeding, this favourable correlation would facilitate an implementation of resilience traits into breeding programs. Correlations between lnvar_weight_ and most other body weight deviation traits were high (*r*_p_ = 0.58–0.88; *r*_g_ = 0.53–0.93) except for skewness (*r*_p_ = 0.01; *r*_g_ = 0.32). These correlations indicate different traits mostly capture the same genetic variation, but some differences exist between traits. Since the weight trajectory parameters straightness and mean speed showed higher *h*^2^ and do not rely on complex modeling, these traits might be more interesting to implement in breeding programs. Additionally, straightness has a favourable genetic correlation with FCR (*r*_g_ = −0.41) and ADG (*r*_g_ = 0.38). Notably, lnMSE_n_visits_ was lowly to negatively correlated with lnMSE_FI_ (*r*_p_ = −0.05, *r*_g_ = −0.34) and lnMSE_dur_ (*r*_p_ = 0.16, *r*_g_ = 0.00). Similar genetic correlations were found by [18]. These findings might imply that more deviations in daily visits to feeding station do not necessarily lead to more variation in the time spent at the AFS and might even reduce deviations in feed intake which is counterintuitive.

It should be noted that our data were collected in purebred pigs in a high health breeding farm. This is in contrast to commercial crossbred finishing pigs, which are typically raised in a more challenging environment with, for example, a higher disease pressure and more social stressors such as a higher pig density. The commercial conditions might elicit more easily differences in resilience [1]. Nonetheless, our data show considerable heritable variation for resilience traits with reasonable predictive ability. However, the purebred-crossbred correlation (*r*_pc)_ of these resilience traits in pigs is not yet known. Research on this topic is essential for pig breeding programs, as an *r*_pc_ < 0.80 indicates crossbred information should be taken into account [42]. For example, in a study on egg production data in layer chicken, an *r*_pc_ was estimated ranging from 0.16–0.47 (lnvar of egg production) to 0.56–0.63 (lag1 autocorrelation) [25]. Furthermore, the main limitation of the present study is that we could not corroborate our resilience traits with resilience related factors such as mortality, disease prevalence, treatments, etc., as done by Putz et al. [16].

Predictive ability analysis using three masking strategies indicated good prospects for selection on most resilience traits (Table 5). The across family masking strategy generally yielded lower predictive abilities compared to the within family masking strategy, as family relationships are more distant in the across family masking strategy. Moreover, adding genotypes to the analysis in general improved predictive abilities. Interestingly, trajectory parameters straightness and mean speed yielded the highest predictive abilities for body weight deviations, demonstrating their potential use for breeding programs. Moreover, resilience indicators for feed intake and feeding behaviour yielded higher predictive abilities. Using single-step genomic evaluation generally improved predictive ability, mainly for the across-family (average increase of +62.2%) and temporal (+67.8%) masking strategy compared to within-family masking (+13.2%). This was expected, as these masking strategies use more distant family information, without own phenotypes and, hence, adding extra genomic information relatively improves predictive ability more [39]. Sae-Lim et al. [40] previously showed that predictive ability of (untransformed) body weight uniformity in salmon could be improved by adding genomic information.

As indicated by Berghof et al. [1], the frequency of observations and observation length are crucial to determine good resilience traits. In our study, we used daily recordings from AFS over a 60-day period within a pigs’ finishing phase (95–155 d). However, AFS may be used more efficiently and/or a limited number of manual weight recordings might be a suitable alternative. Moreover, AFS have not yet been developed and generally used for many livestock species. Therefore, we examined the influence of frequency of observations (Table 6 and Additional file 5: Fig. S3) and length of observation period (Additional file 6: Fig. S4 and Additional file 7: Table S3) by using different data densities and by splitting the dataset in three 20-day periods. If only one record every two weeks or daily records for a short time period would be informative for some resilience traits, these observations could also be collected manually. For example, Berghof et al. [24] used seven weight recordings with a 4-week interval in layer chickens, whereas [43] only had five manual weight recordings of Nile tilapia over a 162-day period. Another option would be to more efficiently use the expensive technology (e.g., AFS), by rotating it over animals so it can be used more efficiently, or by only recording a shorter observation period, although this might pose practical/sanitary issues in pigs. Interestingly, lnvar_weight_standardized_ seemed to be very stable with *r*_p_ > 0.76 and *r*_g_ > 0.96 between full dataset and only one weight recording every two weeks (±5 records in total). These results reiterate the need for data standardization, particularly for traits with a changing average and variance over time such as weight. Whereas trajectory parameters straightness and mean speed seem to have highest *h*^2^ and predictive ability for body weight deviations, these traits are also more sensitive to low data densities, with *r*_p_ = 0.29–0.33 and *r*_g_ = 0.50 for 1 in 14 data density compared to the full dataset. Further, lnMSE_FI_ showed to be quite stable with lower data densities with *r*_p_ = 0.44 and *r*_g_ = 0.84 between full data and 1 in 14 scenario. Moreover, phenotypic and genetic correlations for deviations in feed intake were high over different time periods, with *r*_p_ = 0.48 and *r*_g_ = 0.80 between lnMSE_FI_early_ and lnMSE_FI_late_, and *r*_p_ = 0.73–0.87 and *r*_g_ = 0.90–0.97 between 20-day time periods and the total 60-d period. These results show that, similar to FI, feed intake deviations are moderately repeatable over time: pigs with a high variability in feed intake at the start of the finishing phase, will generally also have a high variability in feed intake at the end of the finishing phase. Observational period and frequency had a large impact on skew_weight_ and lag1_weight_. Therefore, these indicators might not be useful for data with a low observation frequency and/or a short observation period.

In light of our findings, we provided suggestions on the choice of resilience traits to include in a breeding program. The inclusion of resilience traits based on feed intake and feeding behaviour deviations show to be most promising, with highest *h*^2^, GCV and predictive ability. Additionally, these traits seem to be robust to changes in observation frequency and period. However, our study also suggests to include body weight deviations as resilience indicator in breeding programs, as the (genetic) correlations with feed intake and feeding behaviour resilience traits substantially differed from one. We hypothesize that body weight deviations reflect more moderate term responses to external challenges, whereas feed intake and feeding behaviour better reflect short term responses to external stressors. For body weight deviation traits, we recommend to perform a rigid quality control of body weights, as we found that outliers can significantly affect results. Although we provide some guidelines for QC of AFS body weight data, most studies currently still perform an ad-hoc QC. Future work on (more) uniform guidelines for QC could further improve standardization and replicability of results across studies. Regarding quality control of body weights based on AFS data, future studies should focus on more uniform guidelines. We also recommend standardizing weights over time. Finally, the trajectory analysis traits straightness and mean speed showed promise as body weight resilience traits as they had the highest *h*^2^ and predictive ability and a favourable (genetic) correlation with FCR. However, these traits seem more sensitive to observation frequency.

## Conclusions

To our knowledge, this is the first study comparing resilience traits from longitudinal body weight, feed intake and feeding behaviour data in pigs. We showed these resilience traits are lowly to moderately heritable (*h*^2^ = 3%–28%) with good predictive abilities. Moreover, we suggested new, promising resilience indicators based on trajectory analysis with higher *h*^2^ and predictive ability, although these traits were more sensitive to observation frequency. Next, we were the first to report the influence of observation frequency and observation period on these resilience traits and showed that feed intake and feeding duration deviations are very robust to low data density and moderately repeatable over time. Within body weight deviation traits, lnvar_weight_standardized_ seemed most robust to low data density, stressing the need for weight standardization over age when quantifying body weight deviations. Our results can help the design of future studies to look at the relationship between these resilience traits and resilience-related traits such as mortality and disease incidence, and to estimate the purebred-crossbred correlation. We believe our findings will be very useful for pig breeding programs, and will aid in the improvement of pigs’ general resilience by selective breeding. We recommend the inclusion of resilience indicators from both feed intake and body weight deviations in breeding programs, as they could offer valuable insights into different aspects of pigs' resilience. Moreover, we are confident our methodology can be extended to other species as well.

## Supplementary Information

**Additional file 1: Fig. S1. **Gompertz growth curve distribution and parameters.

**Additional file 2: Fig. S2. **Quantile regressionof feed intake and visit duration.

**Additional file 3: Table S1. **Genetic correlations between all trait combinations using bivariate models.

**Additional file 4: Table S2. **Predictive abilities as Pearson correlation between masked breeding values and corrected phenotype.

**Additional file 5: Fig. S3. **Pairwise correlation plots for all evaluated traits with full datasets and reduced datasets.

**Additional file 6: Table S3. **Estimated genetic correlations between the full dataset and reduced datasets.

**Additional file 7: Fig. S4. **Pairwise correlation plots for all evaluated traits with full datasets and reduced datasets.

## Data Availability

The datasets generated and/or analysed during the current study are not publicly available due to data restriction from Hendrix-Genetics but are available from the corresponding author on reasonable request and with permission of Hendrix Genetics.

## Abbreviations

*A*, * B * and *k*: Gompertz growth curve parameters
ADG: Average daily gain
AFI: Average feed intake
AFS: Automated feeding station
BLUP: Best linear unbiased prediction
*c*^2^: Common environmental effect
EBV: Estimated breeding value
FCR: Feed conversion ratio
GCV: Genetic coefficient of variation
*h*^2^: Heritability
lag1_weight_: Lag1 autocorrelation of observed versus predicted weight distribution
lnMSE_dur_: Natural logarithm of mean squared error of visit duration in function of age
lnMSE_FI_: Natural logarithm of mean squared error of feed intake in function of age
lnMSE_n_visit_: Natural logarithm of mean squared error of number of daily visits in function of age
lnMSE_weight_: Natural logarithm of mean squared error of weight in function of age
lnvar_weight_: Natural logarithm of variance of observed versus predicted weights
lnvar_weight_standardized_: Natural logarithm of variance of standardized weights
Mean speed: Mean speed of weight in function of age after trajectory analysis
QC: Quality control
QR_dur_: Number of days with visit duration below 5% of quantile after quantile regression
QR_FI_: Number of days with feed intake below 5% of quantile after quantile regression
*r*_g_: Genetic correlation
RMSE: Root mean square error
*r*_p_: Phenotypic correlation
skew_weight_: Skewness of observed versus predicted weight distribution
SNP: Single nucleotide polymorphism
ssGBLUP: Single-step genomic best linear unbiased prediction
straightness: Straightness index of weight in function of age after trajectory analysis
$$y^\ast$$: Adjusted phenotype
σ_a_: Additive genetic standard deviation
σ_c_: Common environmental standard deviation
σ_e_: Residual standard deviation

## References

[1] Berghof TVL, Poppe M, Mulder HA (2019). Opportunities to improve resilience in animal breeding programs. Front Genet.

[2] Colditz IG, Hine BC (2016). Resilience in farm animals: Biology, management, breeding and implications for animal welfare. Anim Prod Sci.

[3] Knap PW (2005). Breeding robust pigs. Aust J Exp Agric.

[4] Mulder HA (2017). Is GxE a burden or a blessing? opportunities for genomic selection and big data. J Anim Breed Genet.

[5] Scheffer M, Bolhuis JE, Borsboom D, Buchman TG, Gijzel SM, Goulson D, Kammenga JE, Kemp B, van de Leemput IA, Levin S, Martin CM (2018). Quantifying resilience of humans and other animals. Proc Natl Acad Sci.

[6] Knap PW (2020). The scientific development that we need in the animal breeding industry. J Anim Breed Genet.

[7] Kasper C, Ribeiro D, de Almeida AM, Larzul C, Liaubet L, Murani E (2020). Omics application in animal science—a special emphasis on stress response and damaging behaviour in pigs. Genes.

[8] de Souza Iung LH, Carvalheiro R, de Rezende Neves HH, Mulder HA (2020). Genetics and genomics of uniformity and resilience in livestock and aquaculture species: a review. J Anim Breed Genet.

[9] Bellini S. The pig sector in the European Union. In: Iacolina L, Penrith M-L, Bellini S, Chenais E, Jori F, Montoya M, et al., editors. Understanding and combatting African Swine Fever: A European perspective. Wageningen: Wageningen Academic Publishers; 2021. p. 183–95.

[10] Guy S, Thomson PC, Hermesch S (2012). Selection of pigs for improved coping with health and environmental challenges: Breeding for resistance or tolerance?. Front Genet.

[11] Gorssen W, Maes D, Meyermans R, Depuydt J, Janssens S, Buys N (2021). High heritabilities for antibiotic usage show potential to breed for disease resistance in finishing pigs. Antibiotics.

[12] Flori L, Gao Y, Laloë D, Lemonnier G, Leplat JJ, Teillaud A (2011). Immunity traits in pigs: substantial genetic variation and limited covariation. PLoS ONE.

[13] Bishop SC, Woolliams JA (2014). Genomics and disease resistance studies in livestock. Livest Sci.

[14] Blasco A, Martínez-Álvaro M, García ML, Ibáñez-Escriche N, Argente MJ (2017). Selection for environmental variance of litter size in rabbits. Genet Sel Evol.

[15] Formoso-Rafferty N, Cervantes I, Ibáñez-Escriche N, Gutiérrez JP (2016). Correlated genetic trends for production and welfare traits in a mouse population divergently selected for birth weight environmental variability. Animal.

[16] Putz AM, Harding JCS, Dyck MK, Fortin F, Plastow GS, Dekkers JCM (2019). Novel resilience phenotypes using feed intake data from a natural disease challenge model in wean-to-finish pigs. Front Genet.

[17] Cheng J, Putz AM, Harding JCS, Dyck MK, Fortin F, Plastow GS (2020). Genetic analysis of disease resilience in wean-to-finish pigs from a natural disease challenge model. J Anim Sci.

[18] Homma C, Hirose K, Ito T, Kamikawa M, Toma S, Nikaido S (2021). Estimation of genetic parameter for feed efficiency and resilience traits in three pig breeds. Animal.

[19] Kavlak AT, Uimari P. Improving welfare of pigs through selection for resilience. Book of Abstracts of the 73rd Annual Meeting of the European Federation of Animal Science. 2022;202.

[20] Revilla M, Guillaume L, Loïc F-G, Rafael M-T, Friggens NC. Quantifying growth perturbations over the fattening period in swine via mathematical modelling. Peer Community J. 2022;2:e9.

[21] Laghouaouta H, Pena RN, Ros-Freixedes R, Reixach J, Díaz M, Estany J (2021). A methodology to quantify resilience in growing pigs. Animals.

[22] Elgersma GG, de Jong G, van der Linde R, Mulder HA (2018). Fluctuations in milk yield are heritable and can be used as a resilience indicator to breed healthy cows. J Dairy Sci.

[23] Poppe M, Veerkamp RF, van Pelt ML, Mulder HA (2020). Exploration of variance, autocorrelation, and skewness of deviations from lactation curves as resilience indicators for breeding. J Dairy Sci.

[24] Berghof TVL, Bovenhuis H, Mulder HA (2019). Body weight deviations as indicator for resilience in layer chickens. Front Genet.

[25] Bedere N, Berghof TV, Peeters K, Pinard-van Der Laan MH, Visscher J, David I, Mulder HA (2022). Using egg production longitudinal recording to study the genetic background of resilience in purebred and crossbred laying hens. Genet Sel Evol.

[26] Wen H, Johnson JS, Freitas PHF, Maskal JM, Byrd MK, Tiezzi F, et al. Genetic parameter estimation of various body temperature and respiration rate indicators in maternal-line pigs under heat stress conditions. In Proceedings of 12th World Congress on Genetics Applied to Livestock Production (WCGALP). 2022;528–531.

[27] Gorssen W, Winters C, Meyermans R, D’Hooge R, Janssens S, Buys N (2022). Estimating genetics of body dimensions and activity levels in pigs using automated pose estimation. Sci Rep.

[28] Winsor CP (1932). The gompertz curve as a growth curve. Proc Natl Acad Sci.

[29] R Core Team. R: A language and environment for statistical computing. R Foundation for Statistical Computing, Vienna, Austria. 2020.

[30] Koivula M, Sevón-Aimonen ML, Strandén I, Matilainen K, Serenius T, Stalder KJ, Mäntysaari EA (2008). Genetic (co)variances and breeding value estimation of Gompertz growth curve parameters in Finnish Yorkshire boars, gilts and barrows. J Anim Breed Genet.

[31] McLean DJ, Skowron Volponi MA. trajr: An R package for characterisation of animal trajectories. Tregenza T, editor. Ethology. 2018;124(6):440–8.

[32] Misztal I, Tsuruta S, Lourenco D, Aguilar I, Legarra A, Vitezica Z (2014). Manual for BLUPF90 family of programs.

[33] Houle D (1992). Comparing evolvability and variability of quantitative traits. Genetics.

[34] Mulder HA, Bijma P, Hill WG (2007). Prediction of breeding values and selection responses with genetic heterogeneity of environmental variance. Genetics.

[35] Hill WG, Mulder HA (2010). Genetic analysis of environmental variation. Genet Res.

[36] Aguilar I, Misztal I, Johnson DL, Legarra A, Tsuruta S, Lawlor TJ (2010). Hot topic: a unified approach to utilize phenotypic, full pedigree, and genomic information for genetic evaluation of Holstein final score. J Dairy Sci.

[37] Christensen OF, Lund MS (2010). Genomic prediction when some animals are not genotyped. Genet Sel Evol.

[38] Legarra A, Aguilar I, Misztal I (2009). A relationship matrix including full pedigree and genomic information. J Dairy Sci.

[39] Legarra A, Robert-Granié C, Manfredi E, Elsen JM (2008). Performance of genomic selection in mice. Genetics.

[40] Sae-Lim P, Kause A, Lillehammer M, Mulder HA (2017). Estimation of breeding values for uniformity of growth in Atlantic salmon (Salmo salar) using pedigree relationships or single-step genomic evaluation. Genet Sel Evol.

[41] Lande R (1977). On comparing coefficients of variation. Syst Zool.

[42] Wientjes YCJ, Calus MPL (2017). Board invited review: the purebred-crossbred correlation in pigs: a review of theory, estimates, and implications. J Anim Sci.

[43] Mengistu SB, Mulder HA, Bastiaansen JW, Benzie JA, Khaw HL, Trinh TQ, Komen H (2022). Fluctuations in growth are heritable and a potential indicator of resilience in Nile tilapia (Oreochromis niloticus). Aquaculture.

