# Supplementary figures and images for: A promising resilience parameter for breeding: the use of weight and feed trajectories in growing pigs
Source: J Anim Sci Biotechnol. 2023 Aug 1;14:101. doi: 10.1186/s40104-023-00901-9 (PMC10391771; doi:10.1186/s40104-023-00901-9)

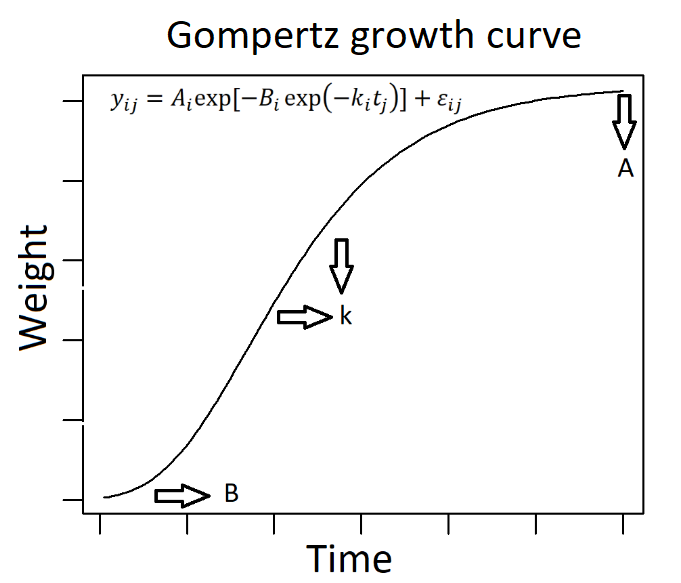

Supplement: Supplementary file 1 — Additional file 1: Fig. S1. Gompertz growth curve distribution and parameters. [file 40104_2023_901_MOESM1_ESM.tif]

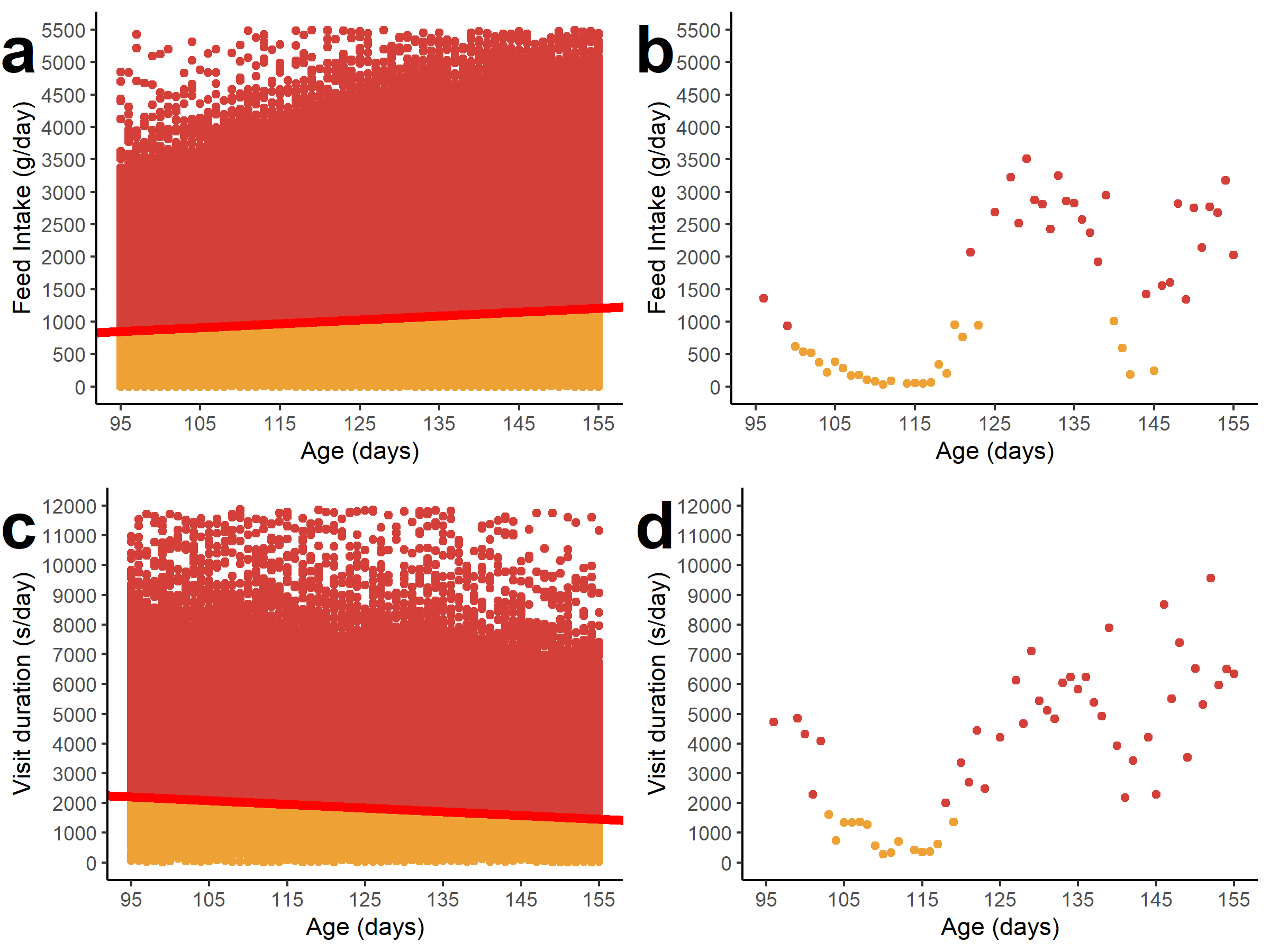

Supplement: Supplementary file 2 — Additional file 2: Fig. S2. Quantile regressionof feed intake and visit duration. [file 40104_2023_901_MOESM2_ESM.png]

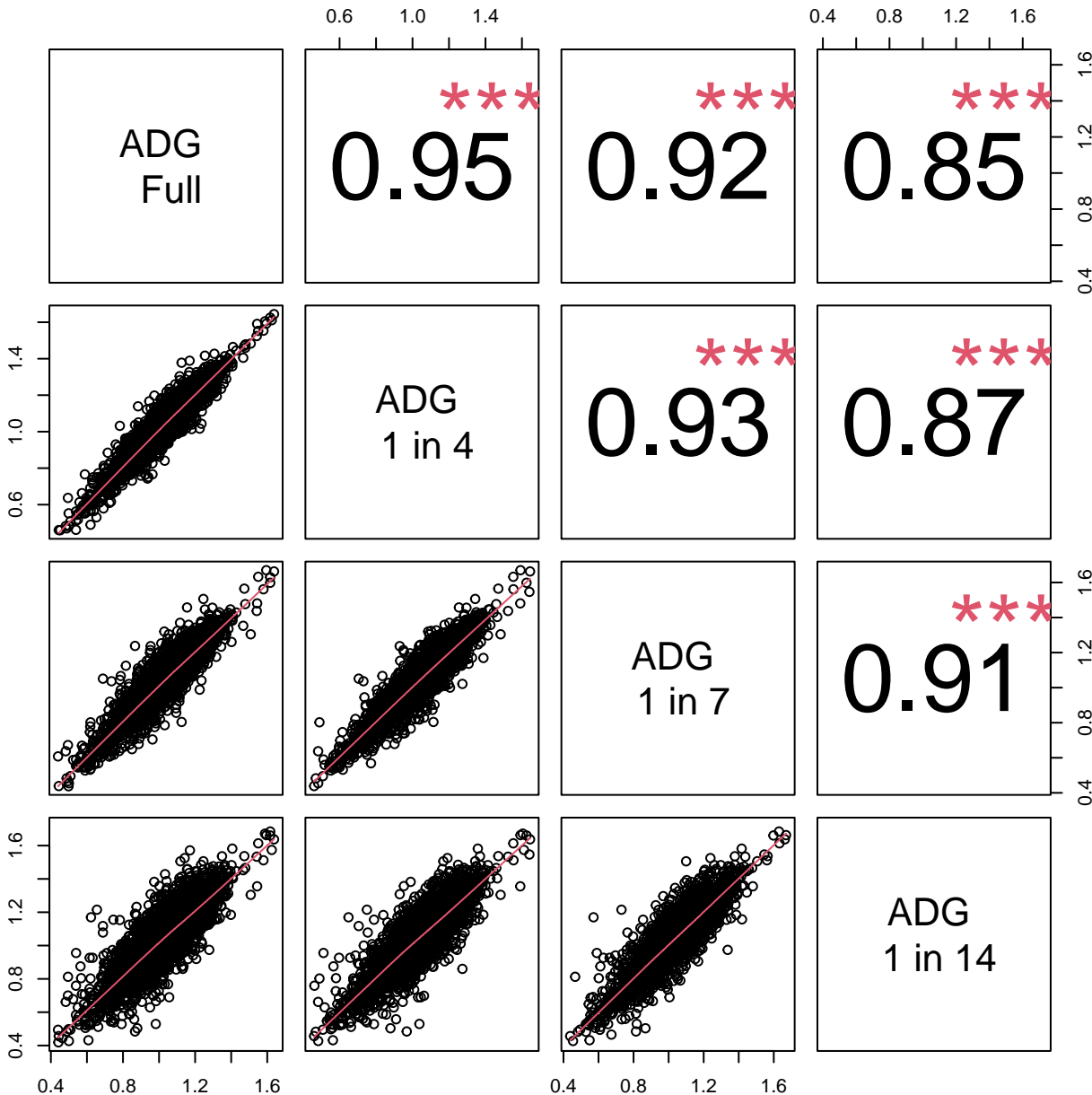

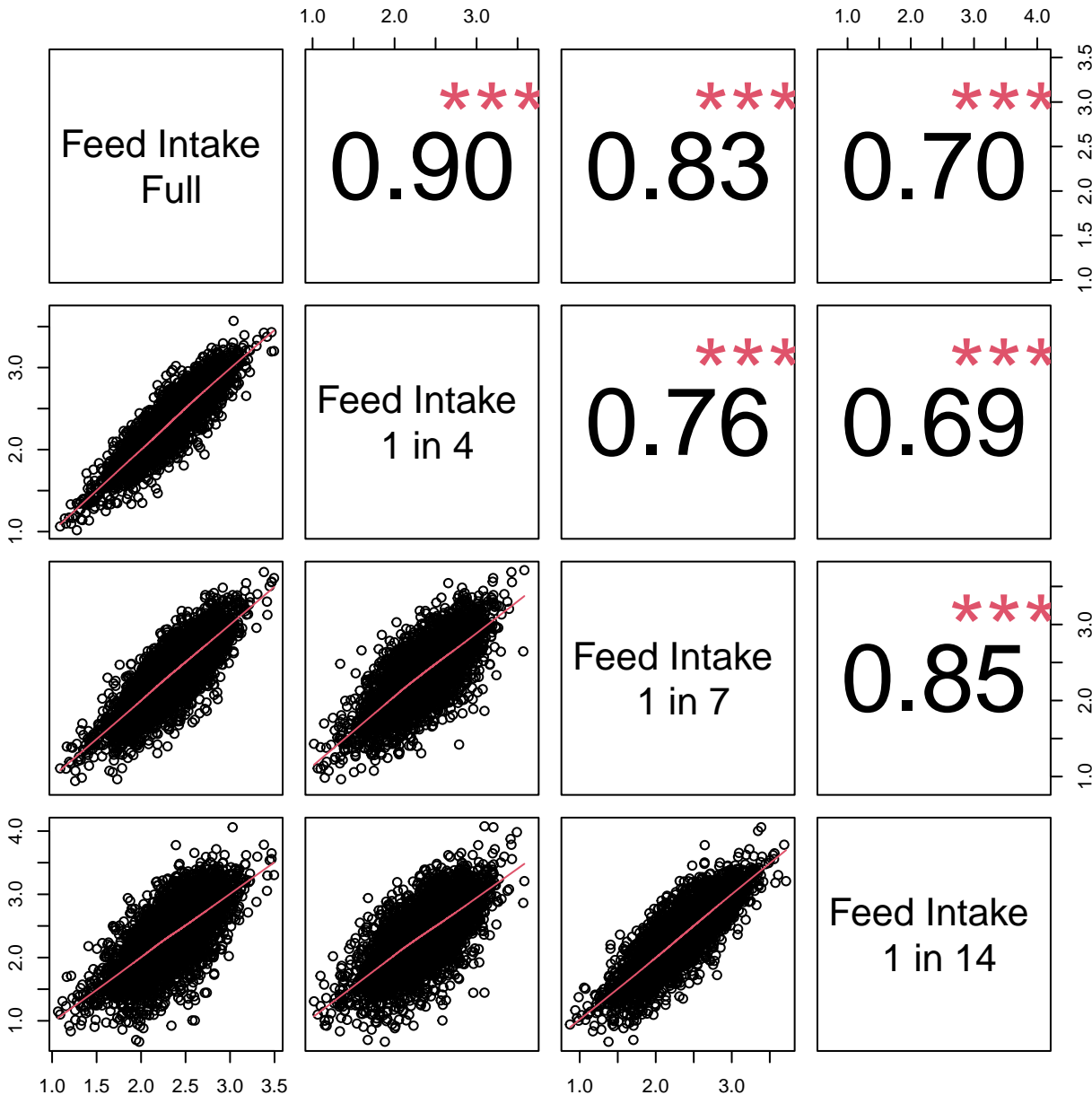

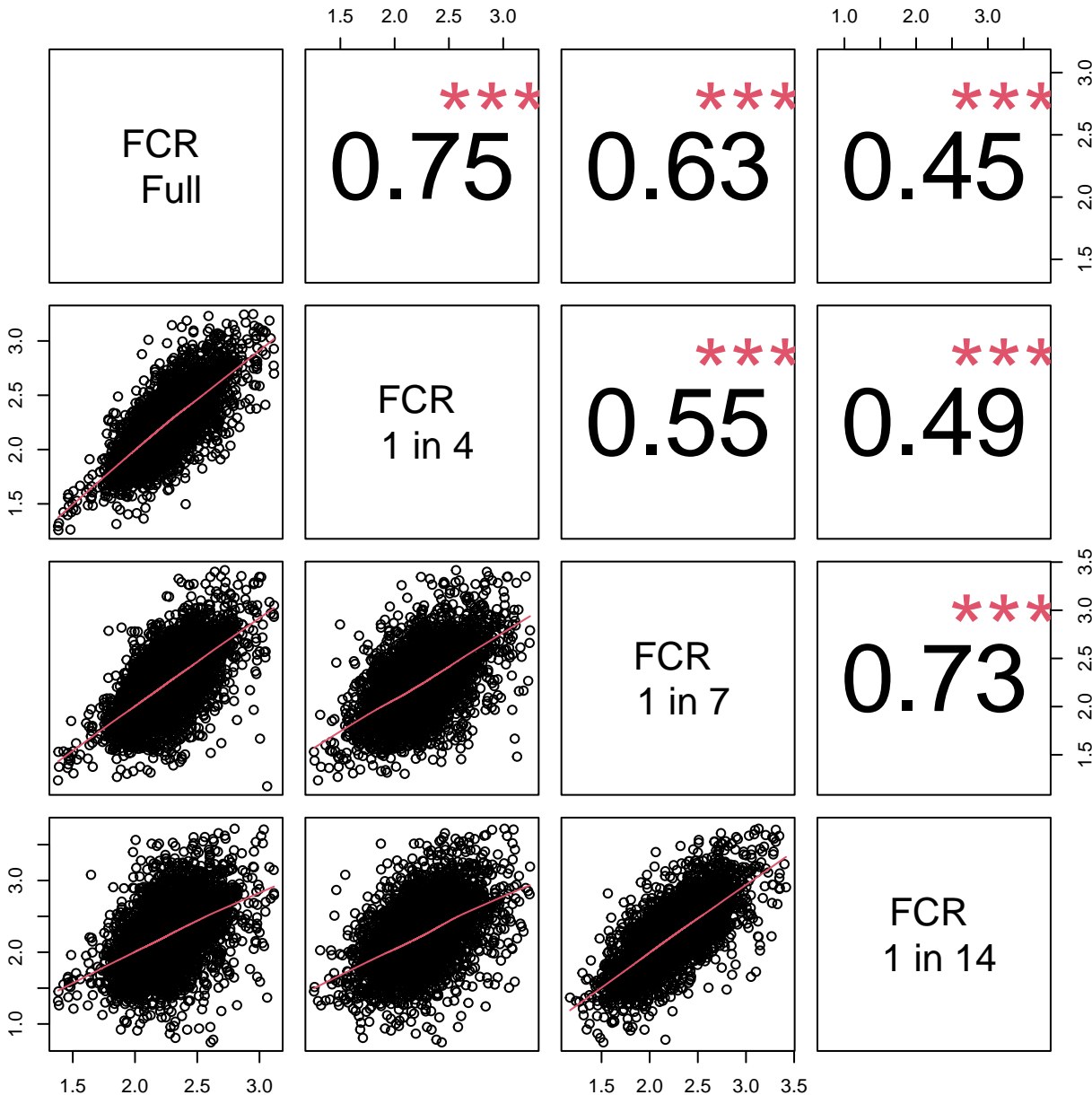

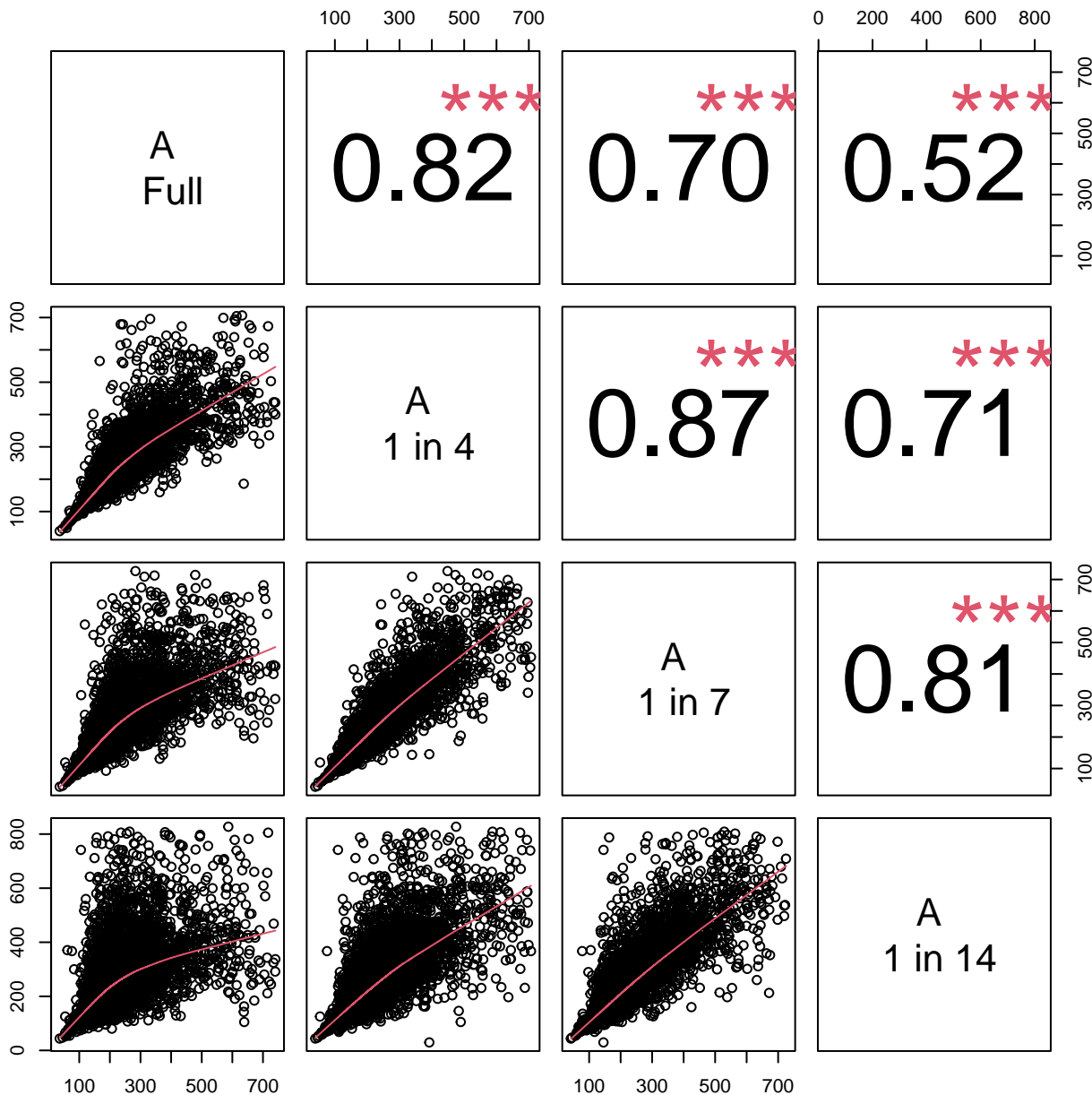

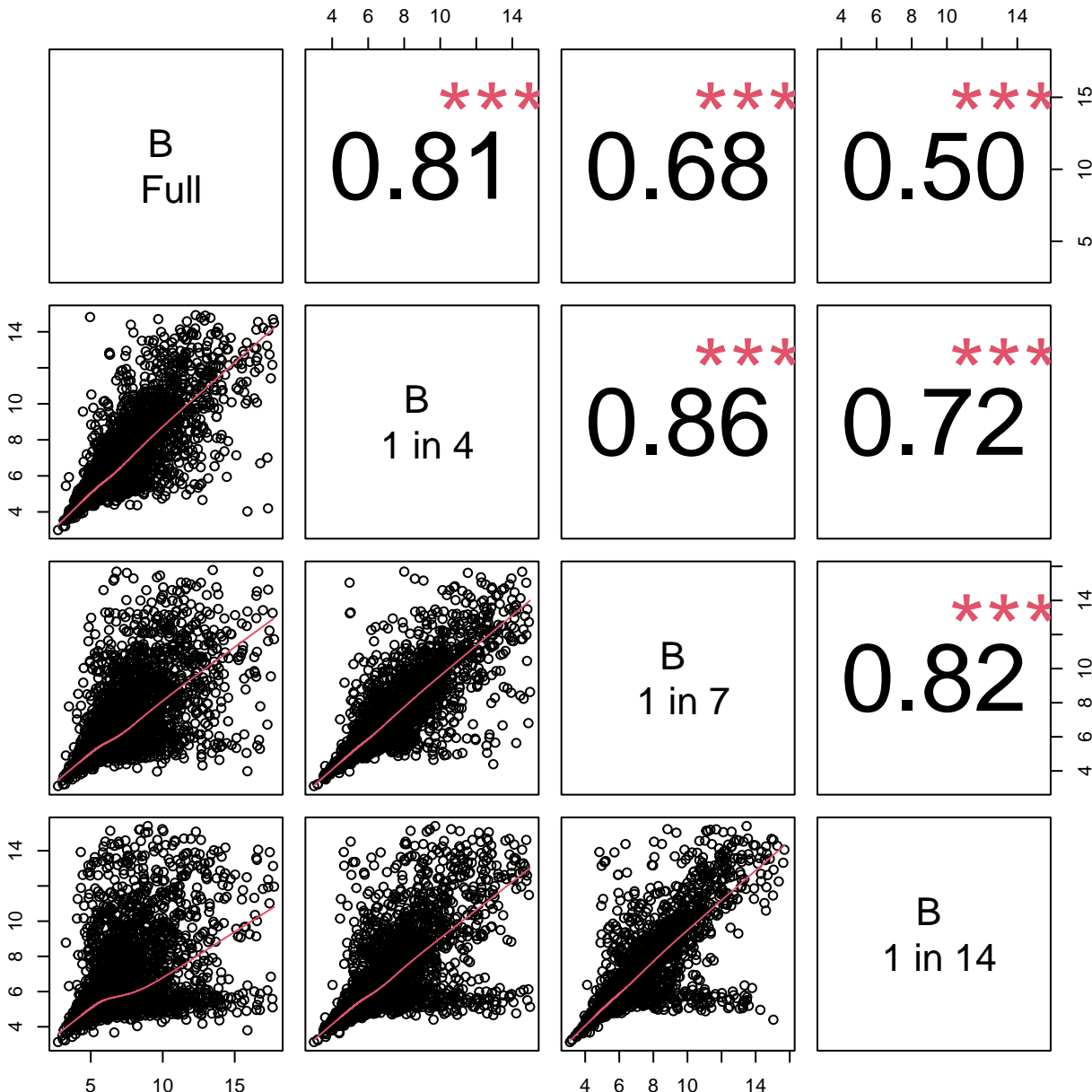

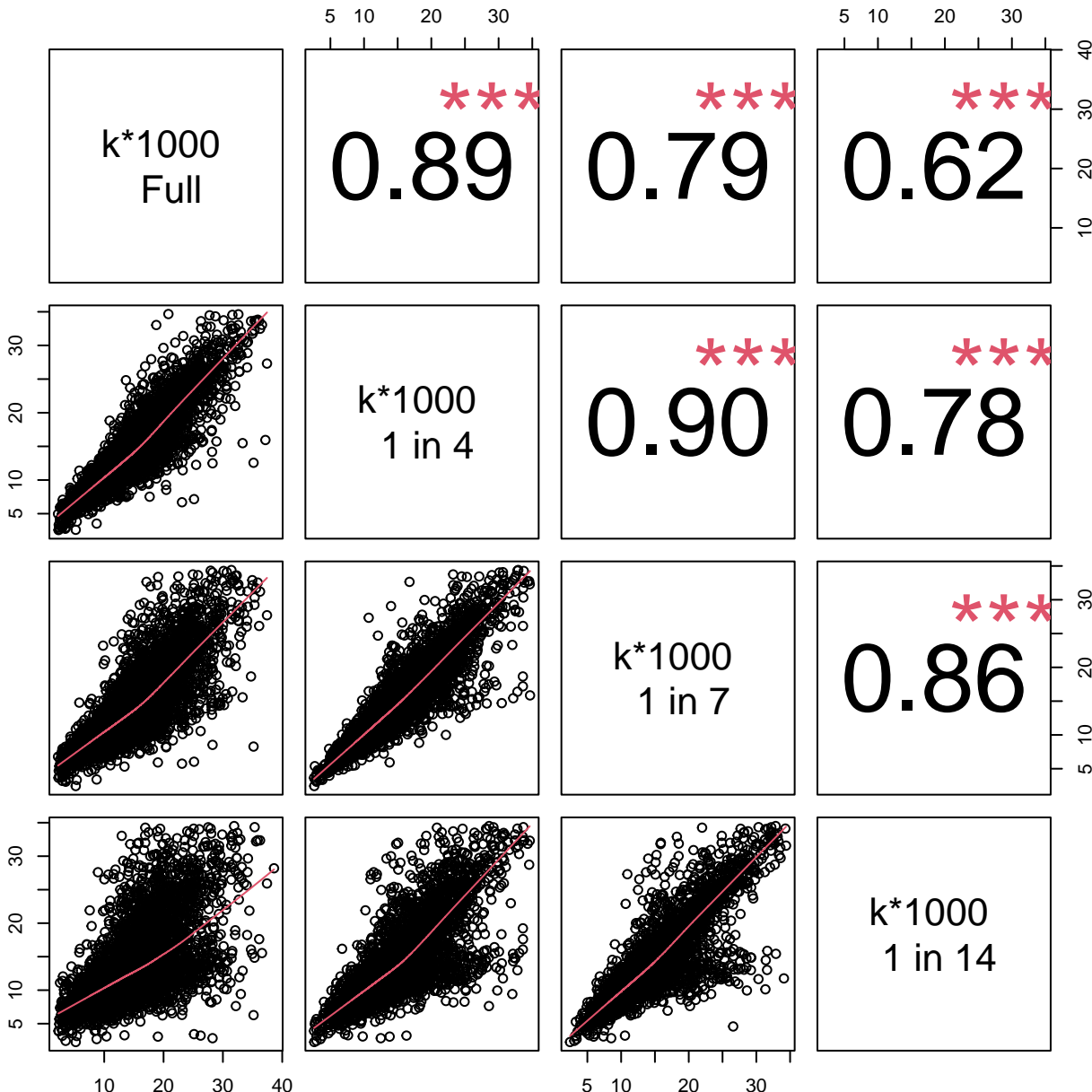

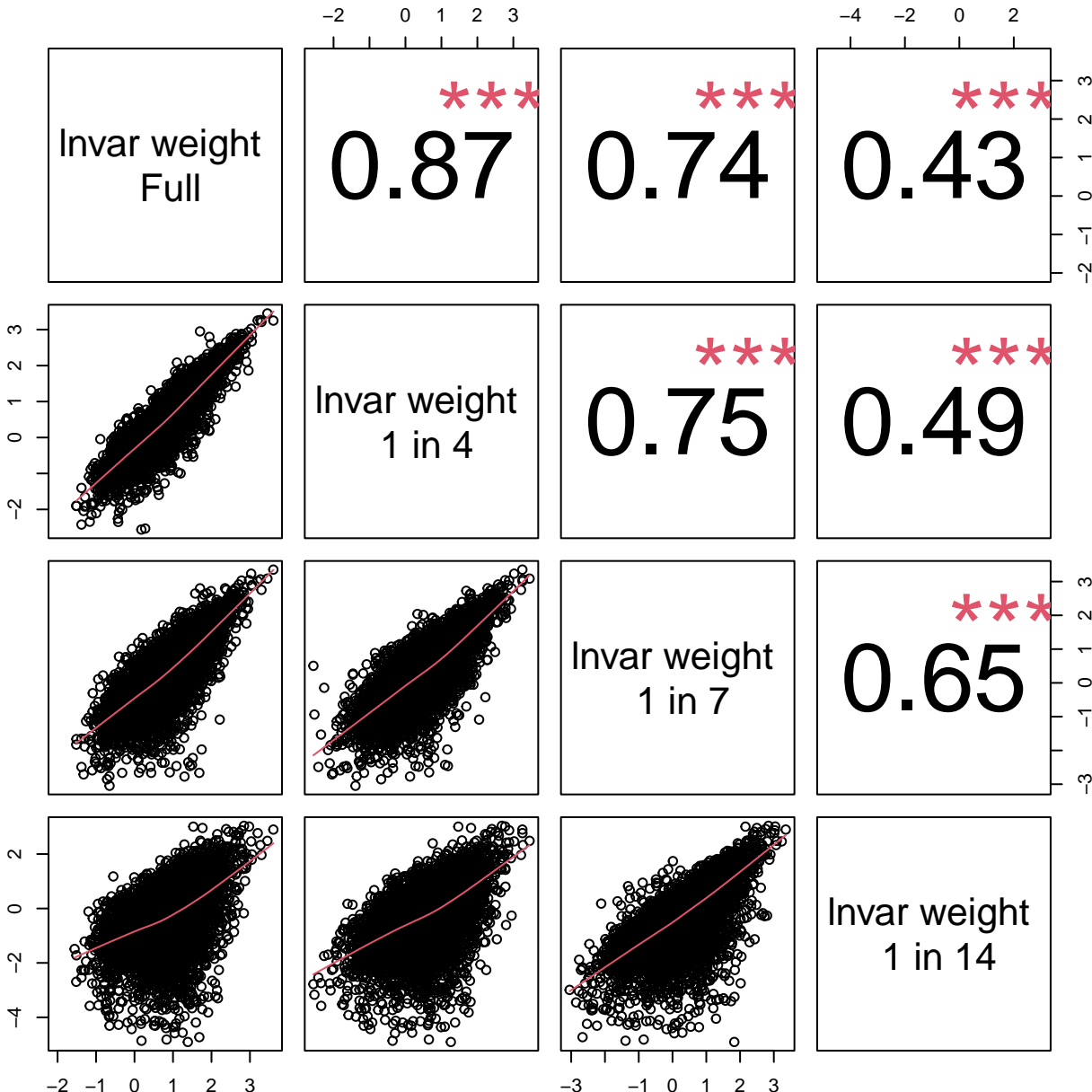

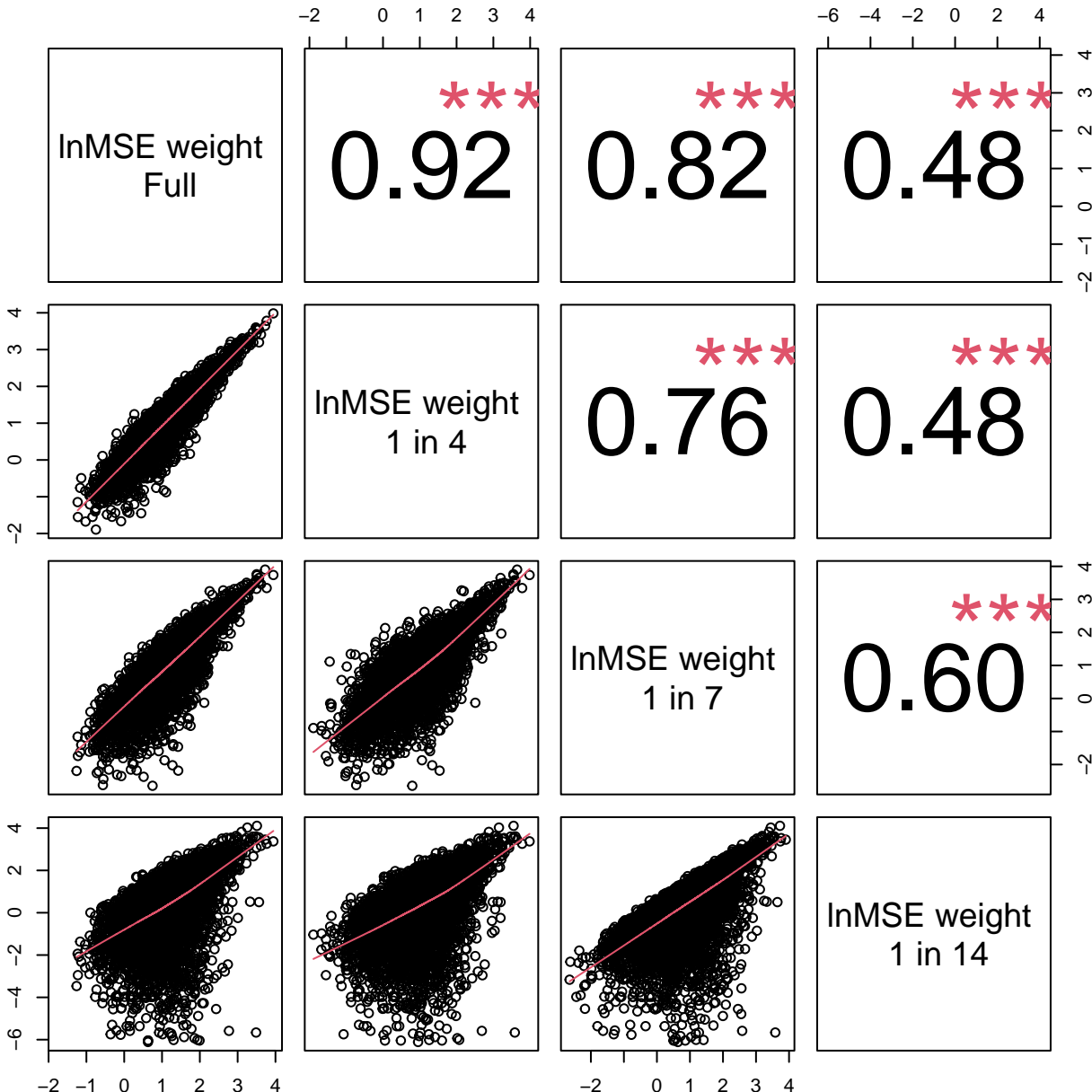

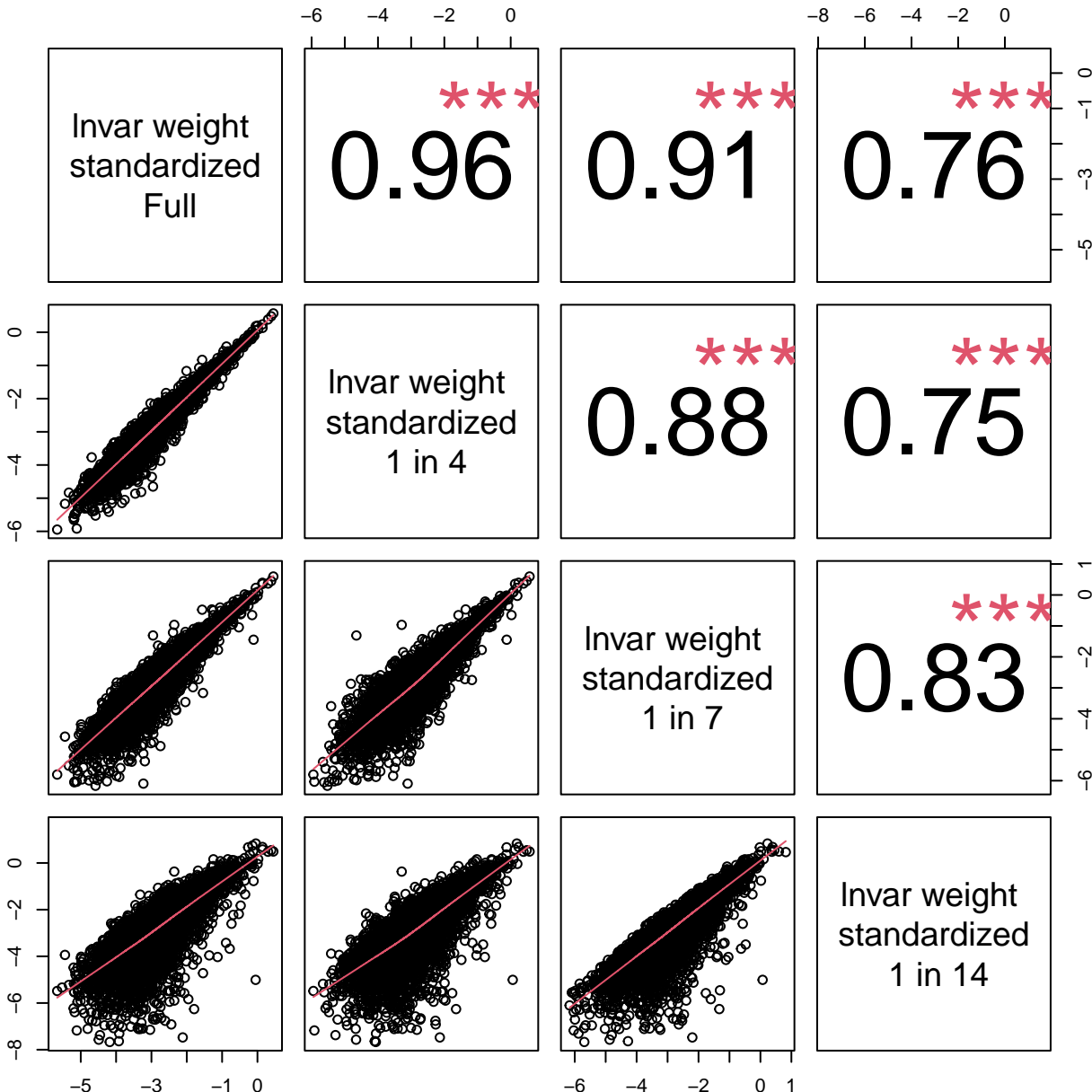

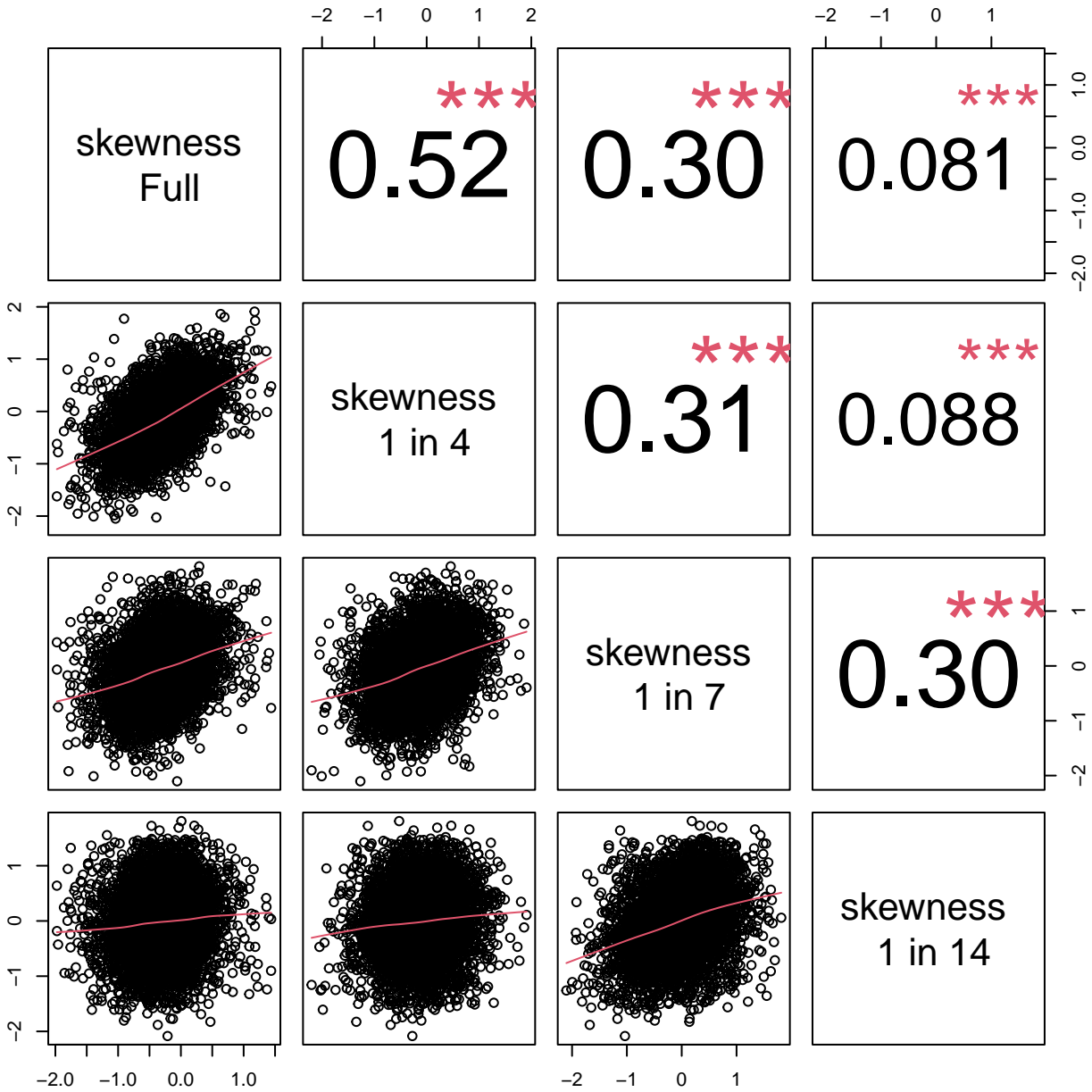



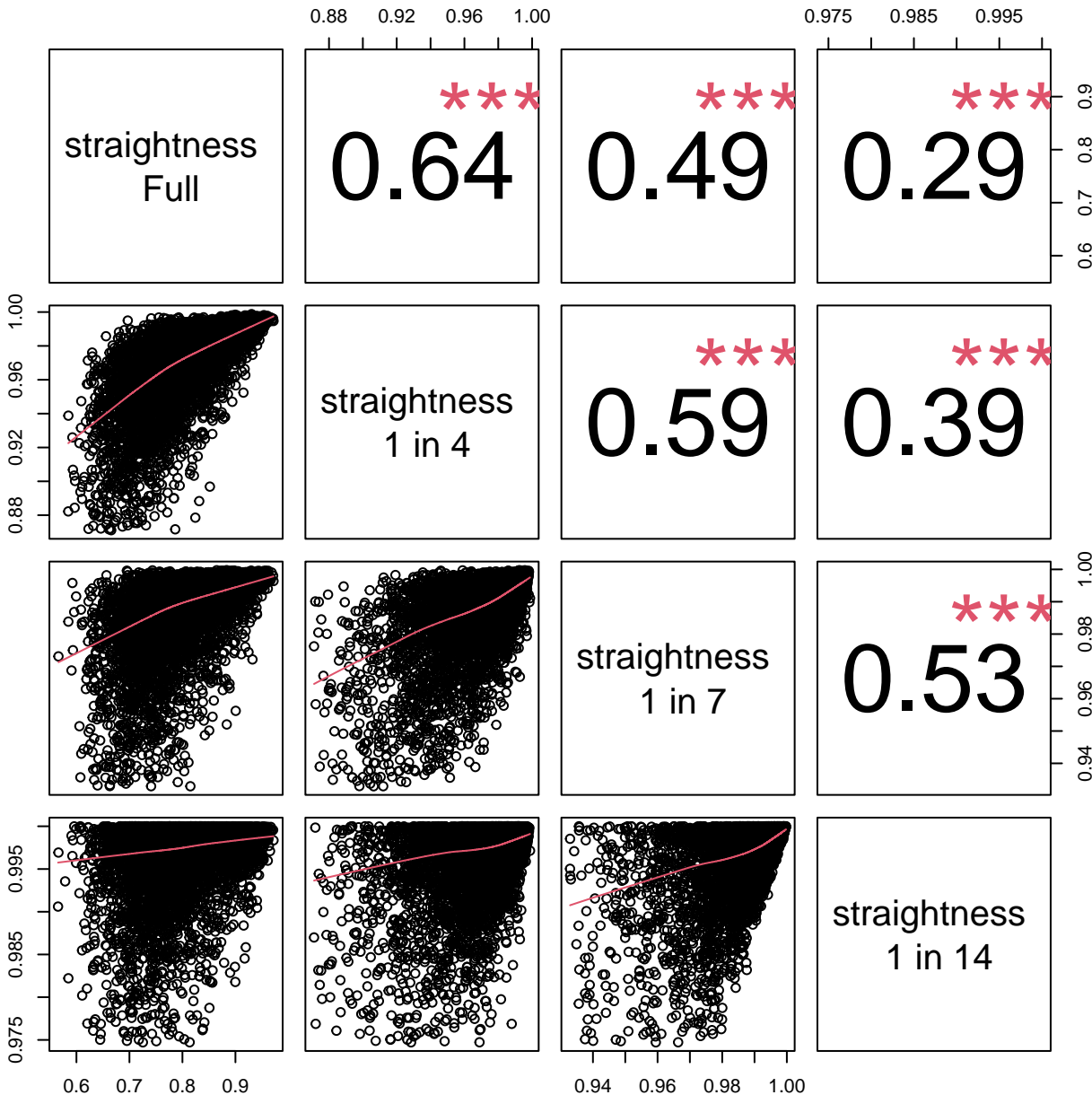



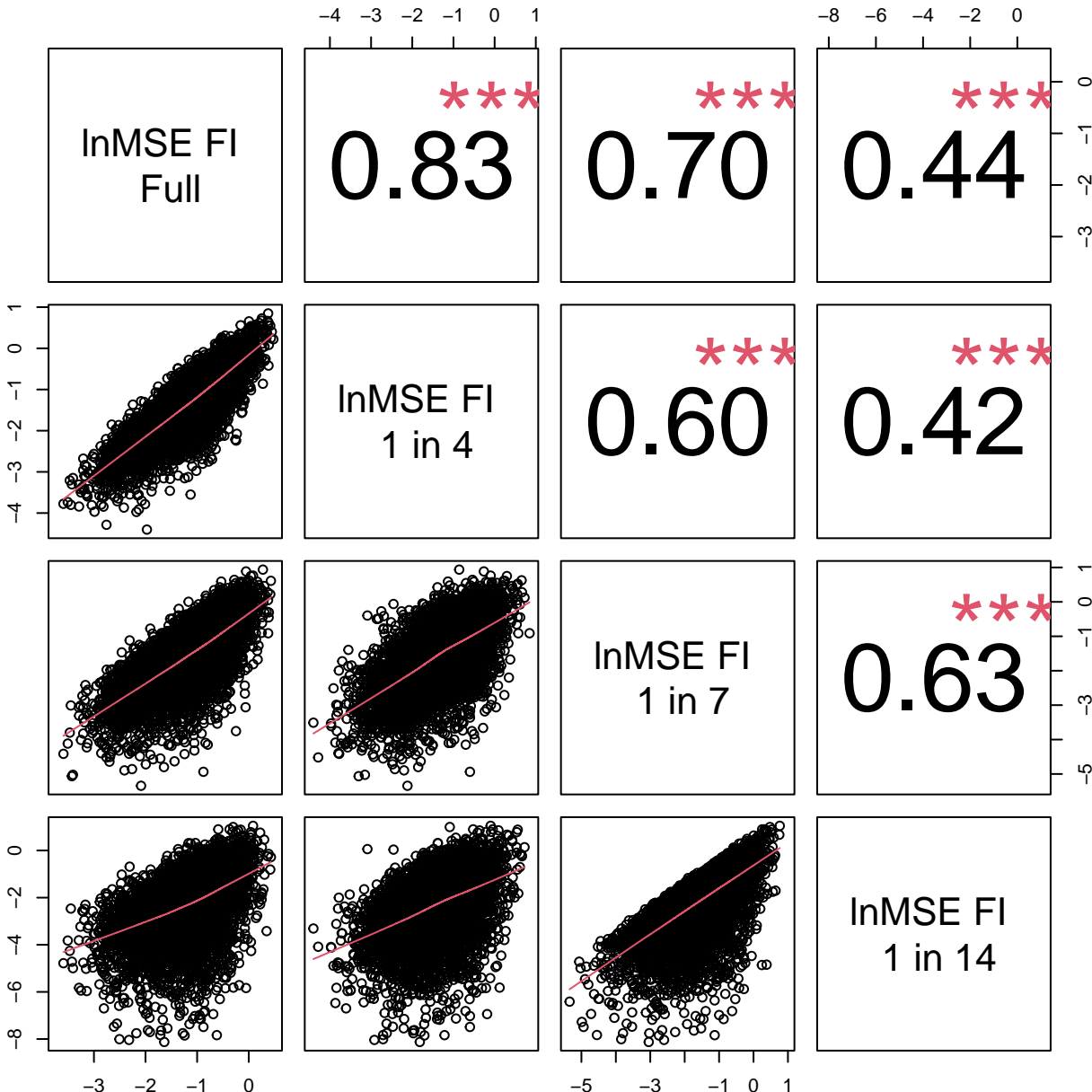

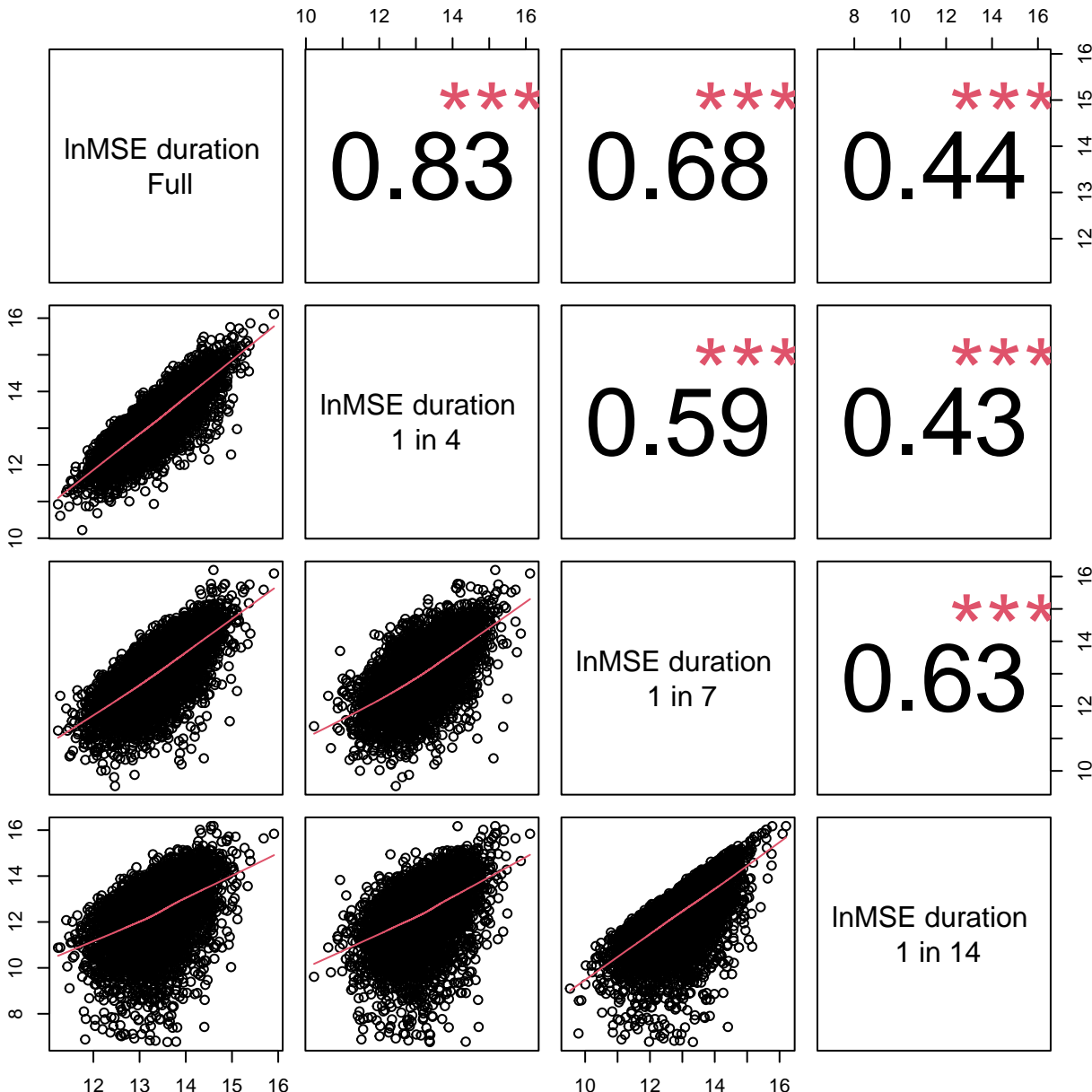

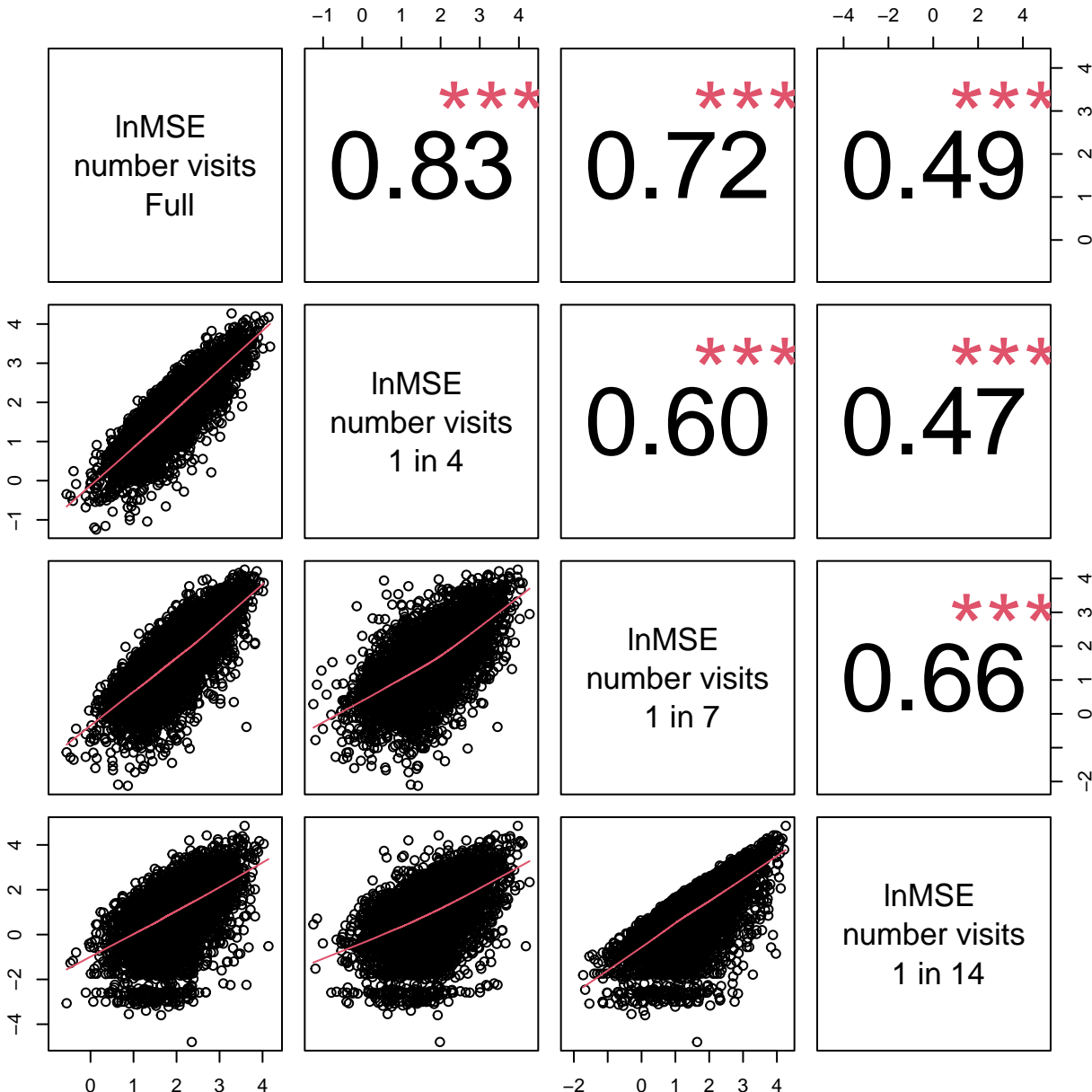

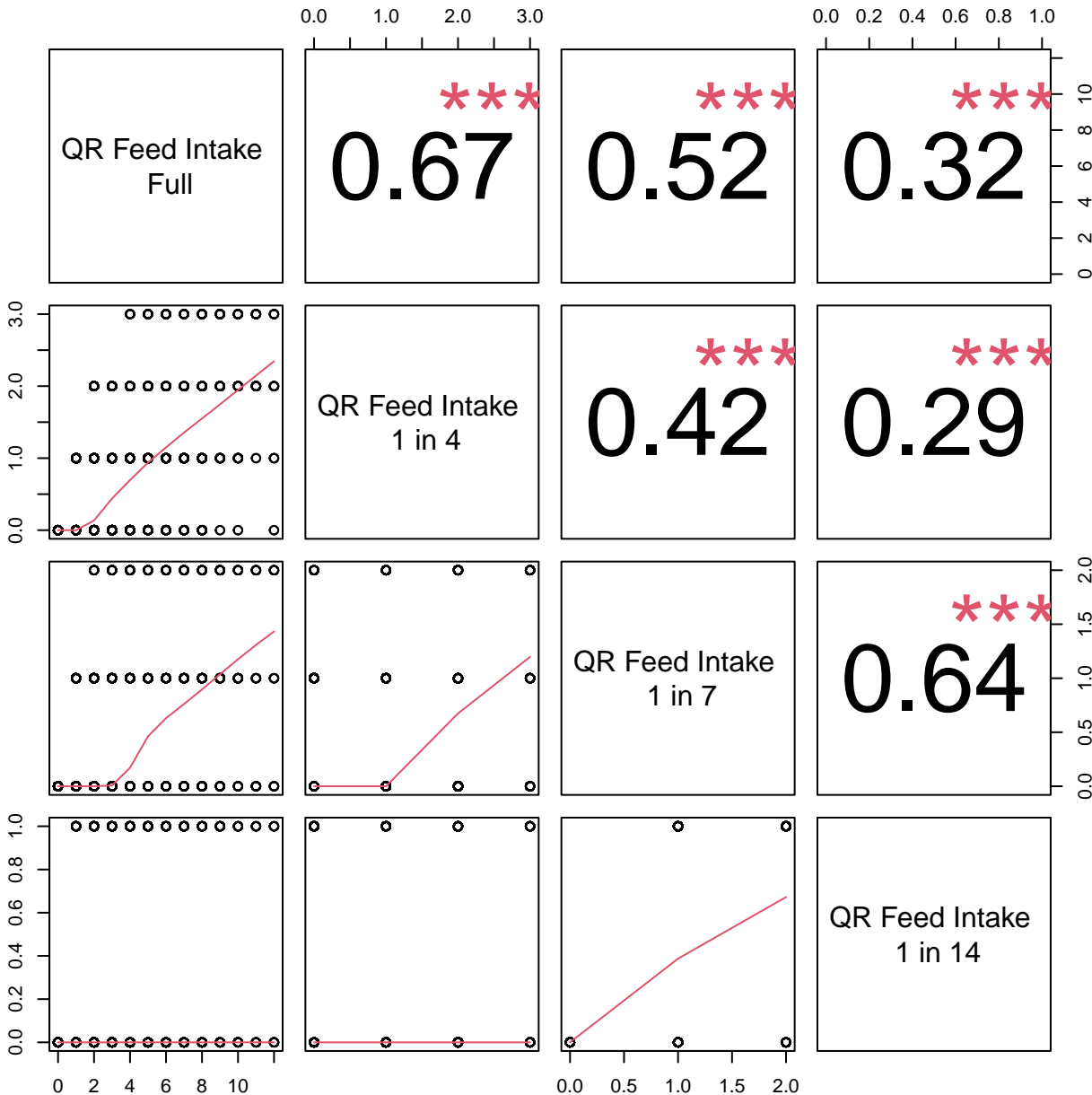

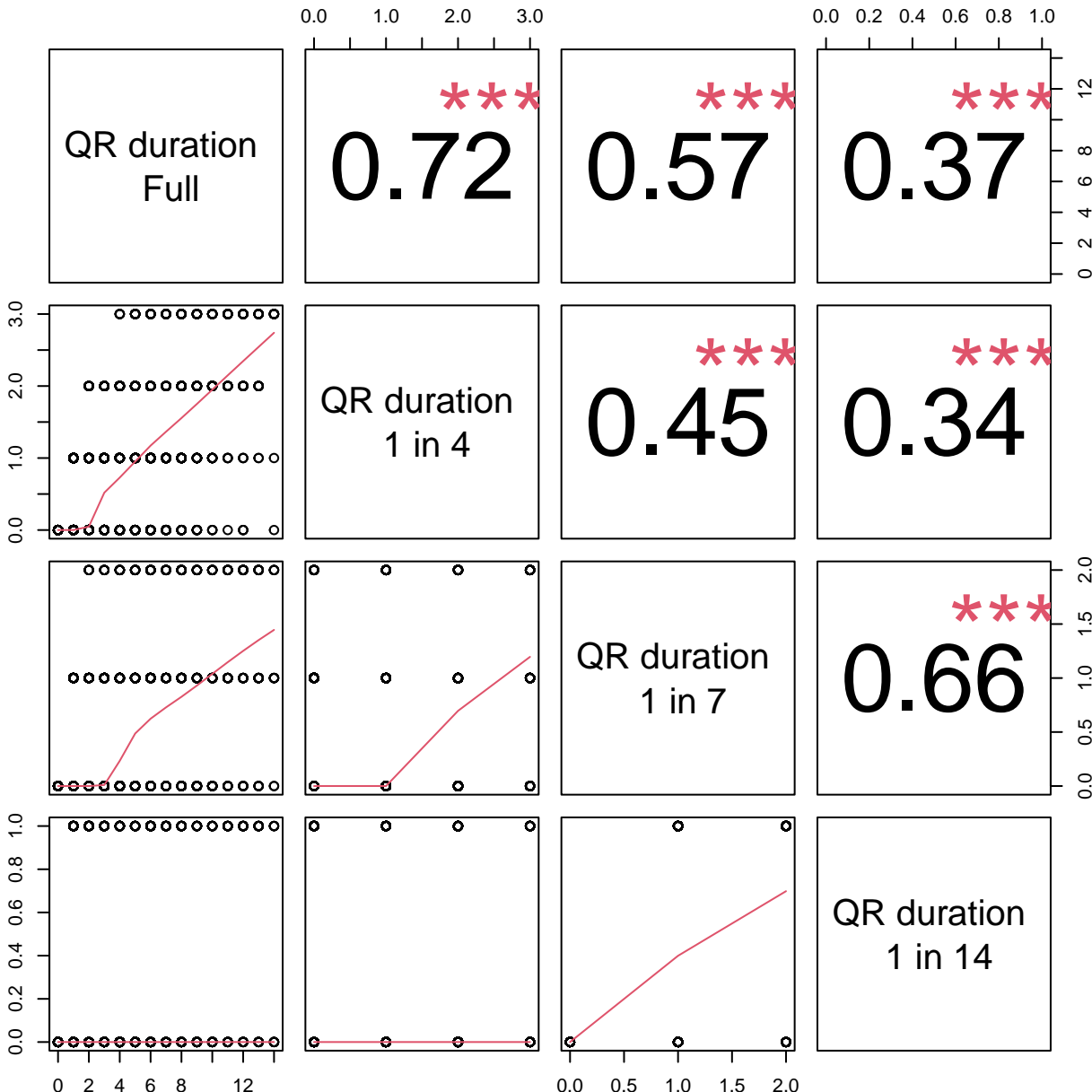

Supplement: Supplementary file 5 — Additional file 5: Fig. S3. Pairwise correlation plots for all evaluated traits with full datasets and reduced datasets. [file 40104_2023_901_MOESM5_ESM.pdf]

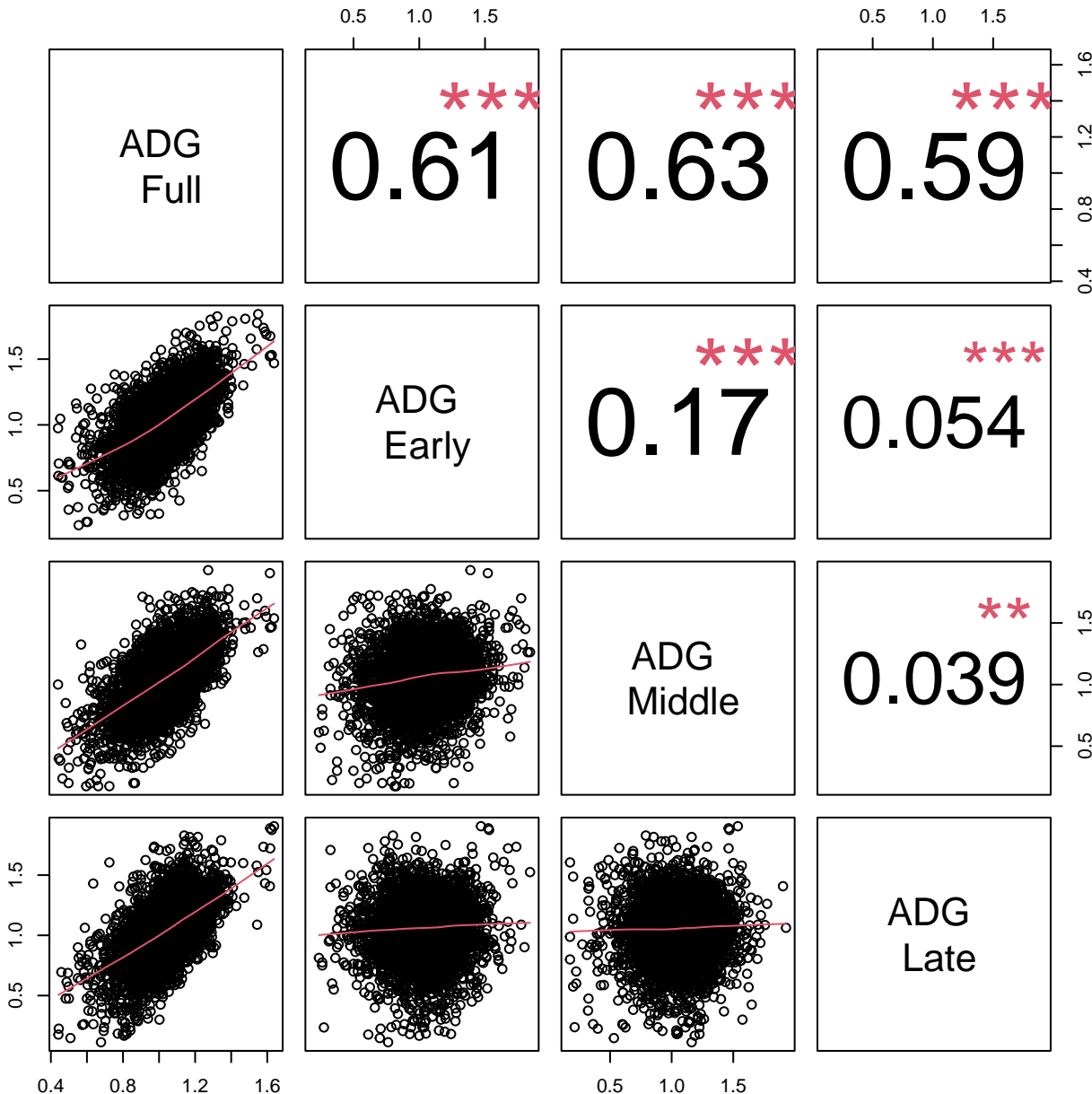



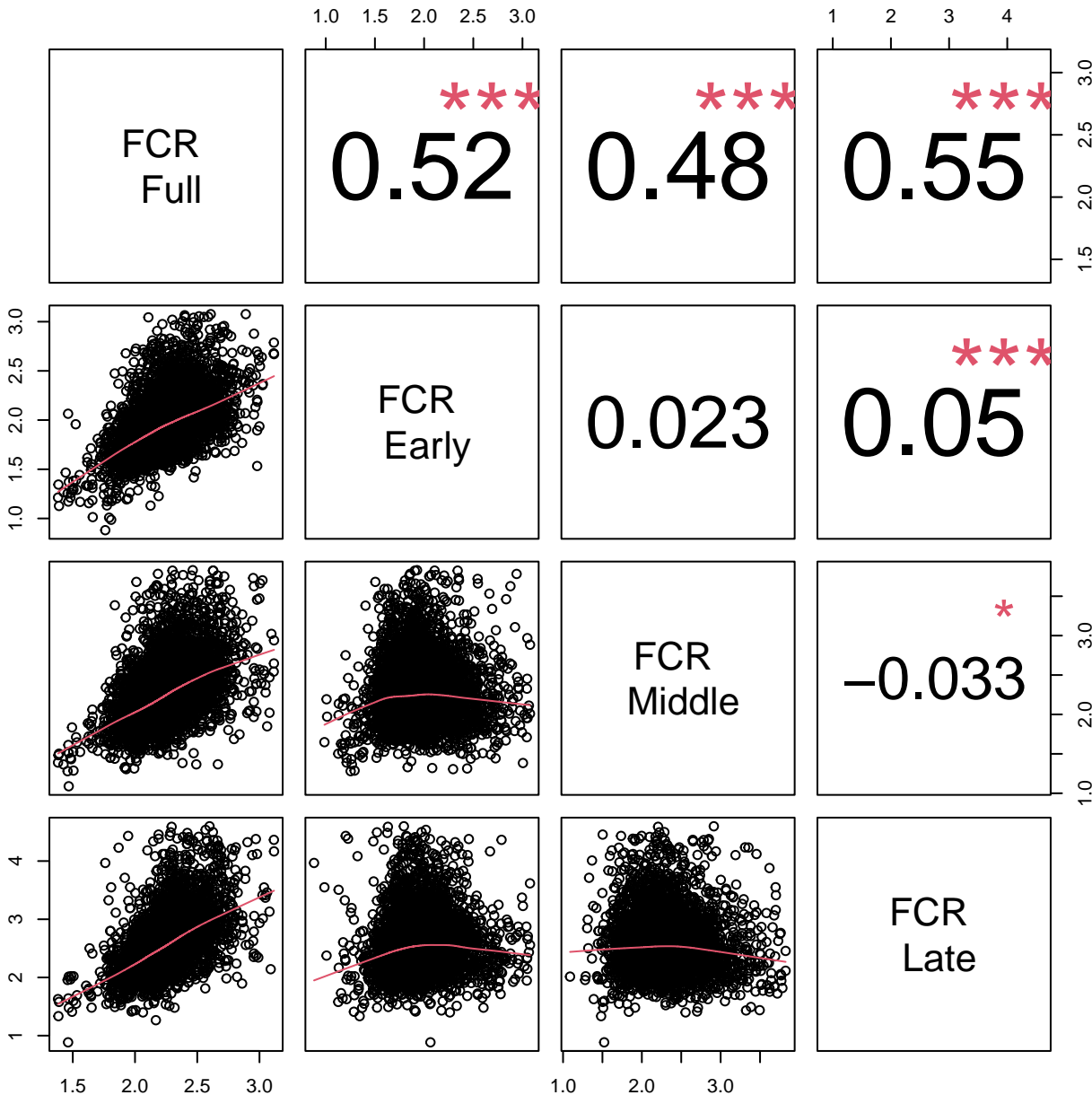

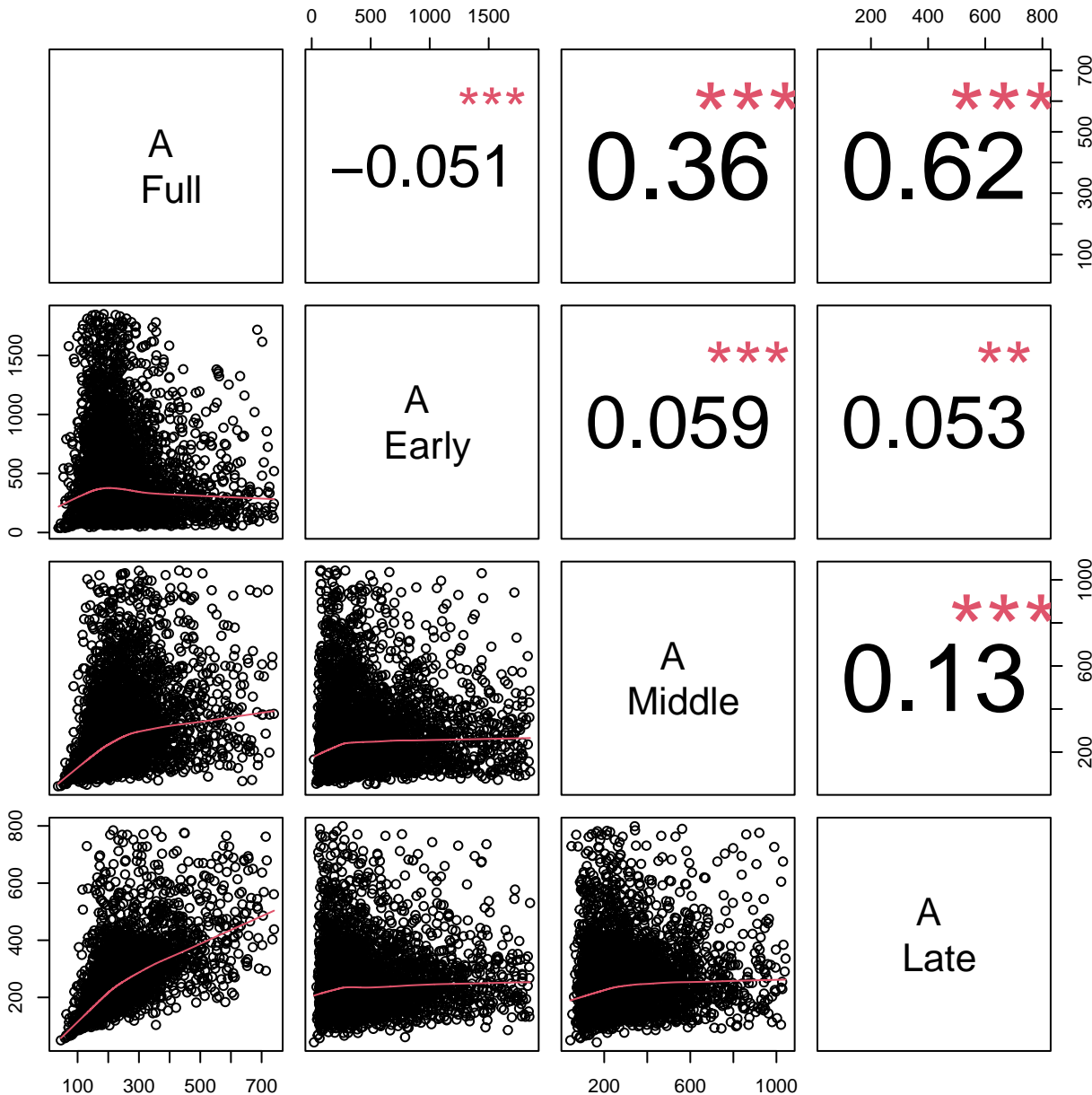

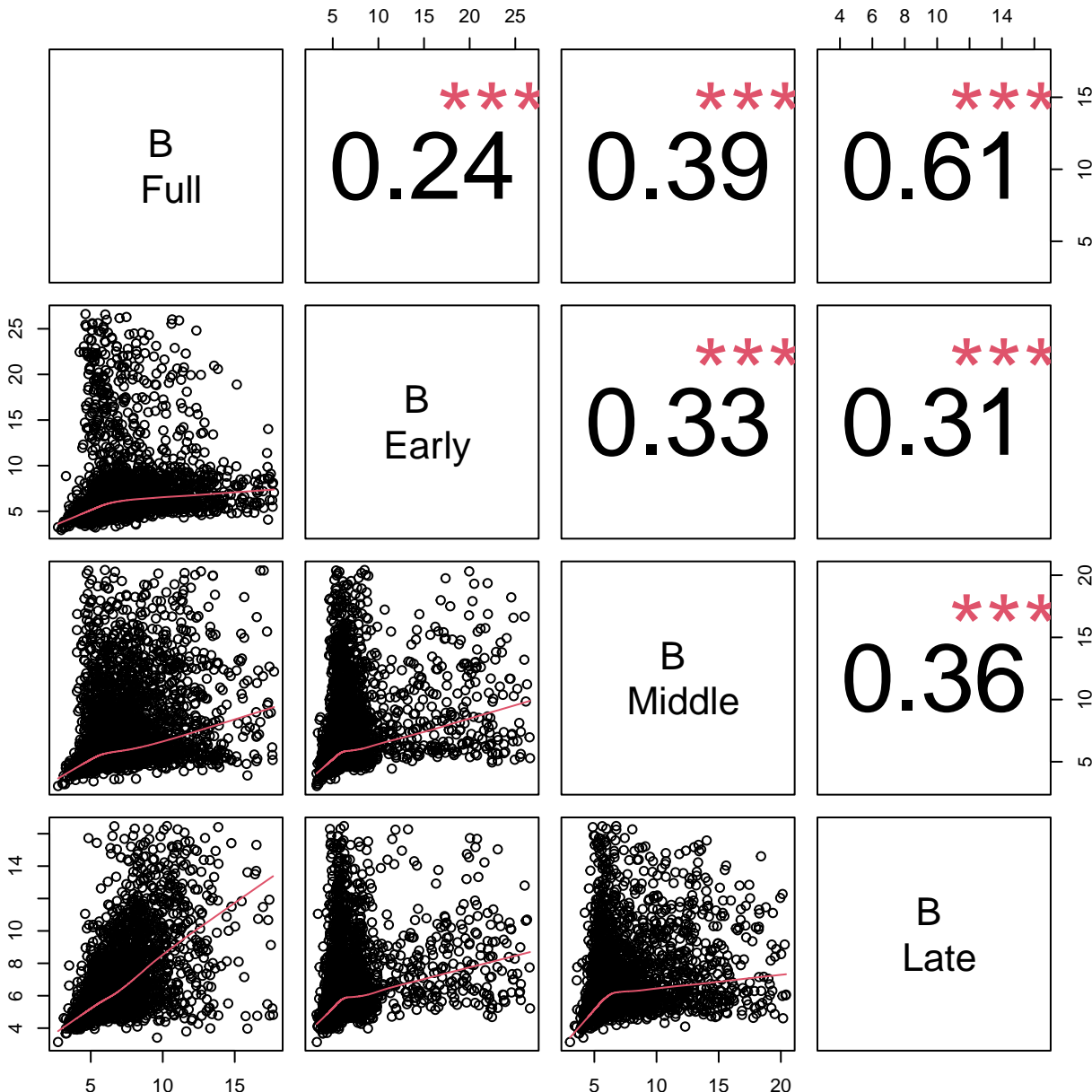

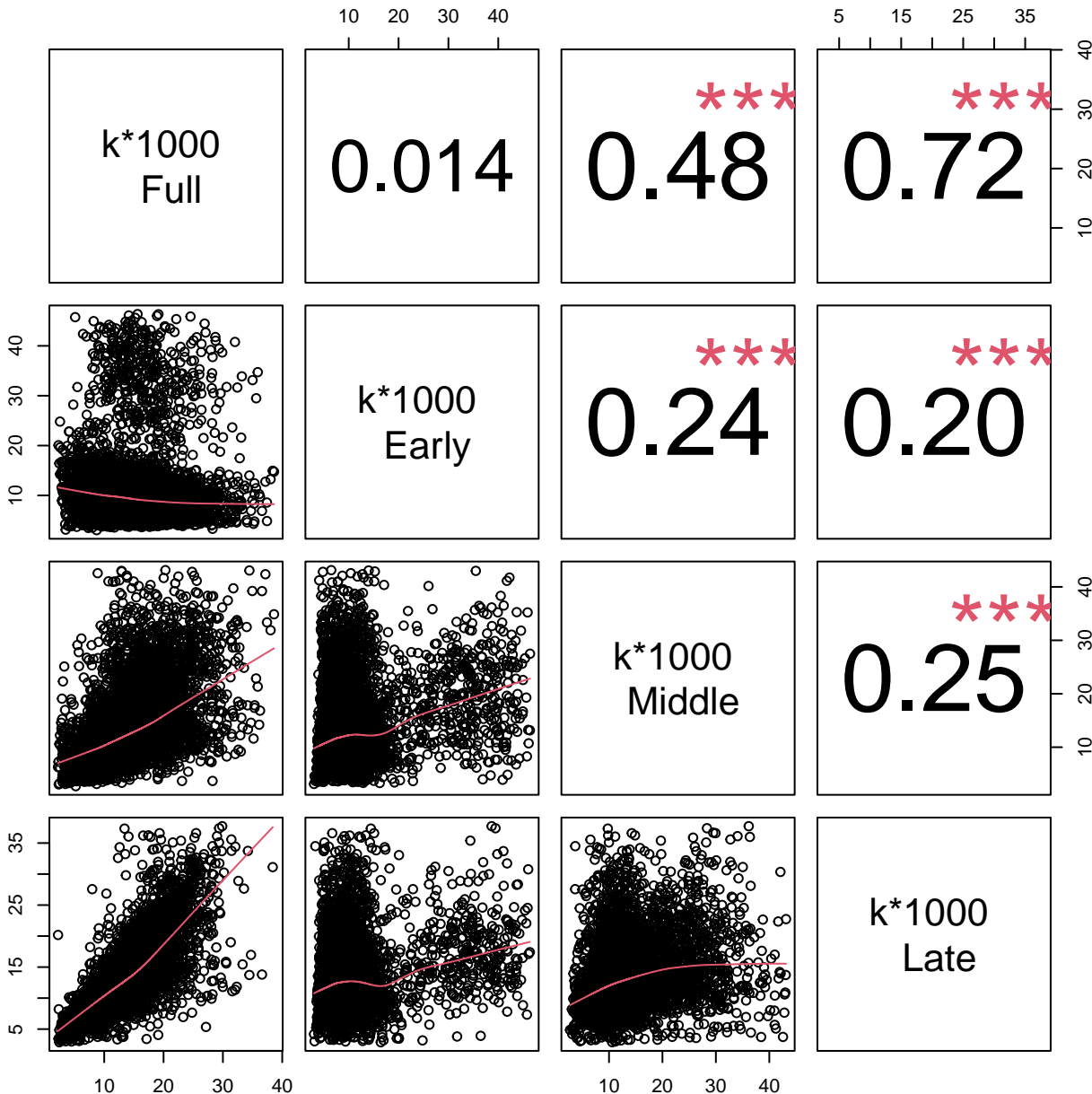

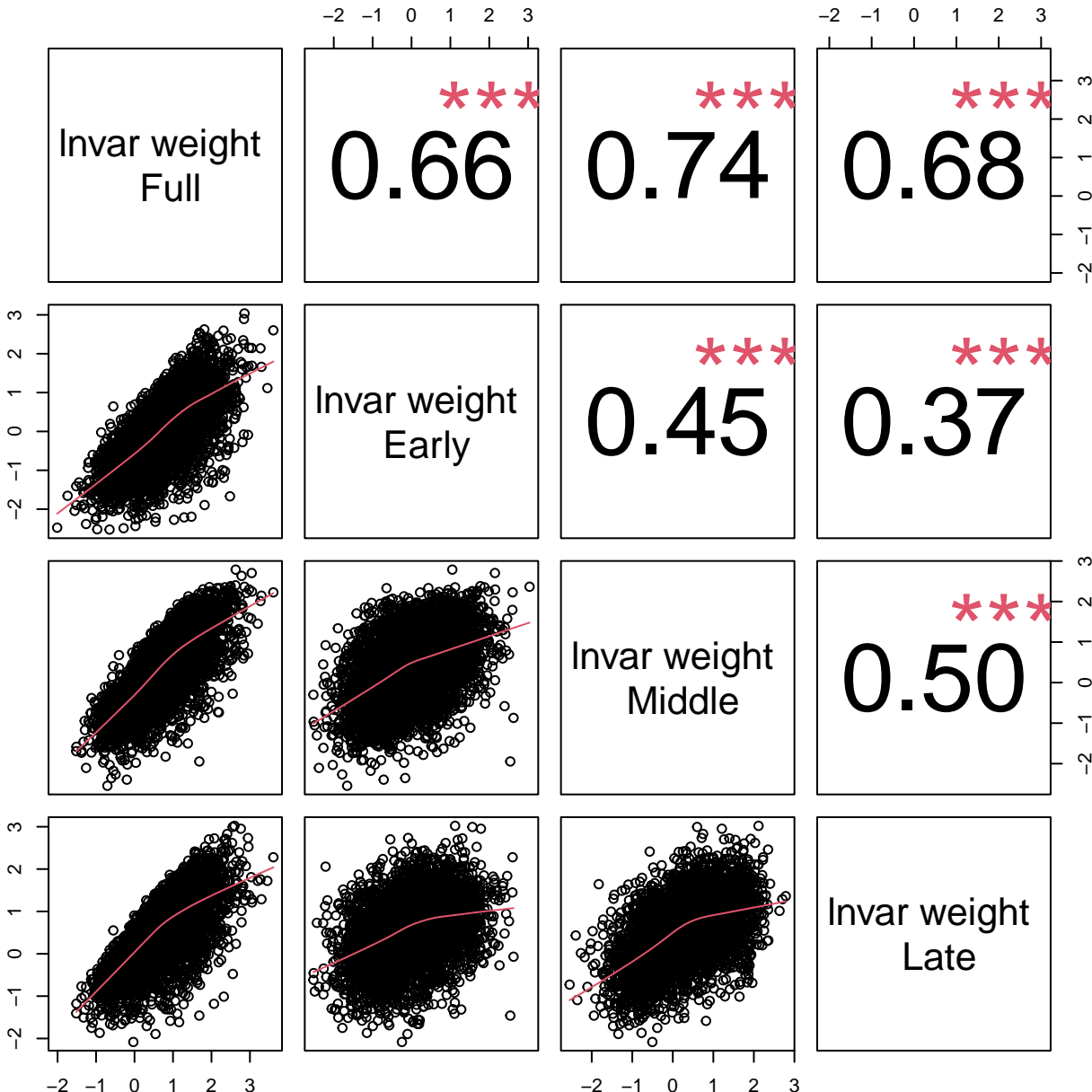

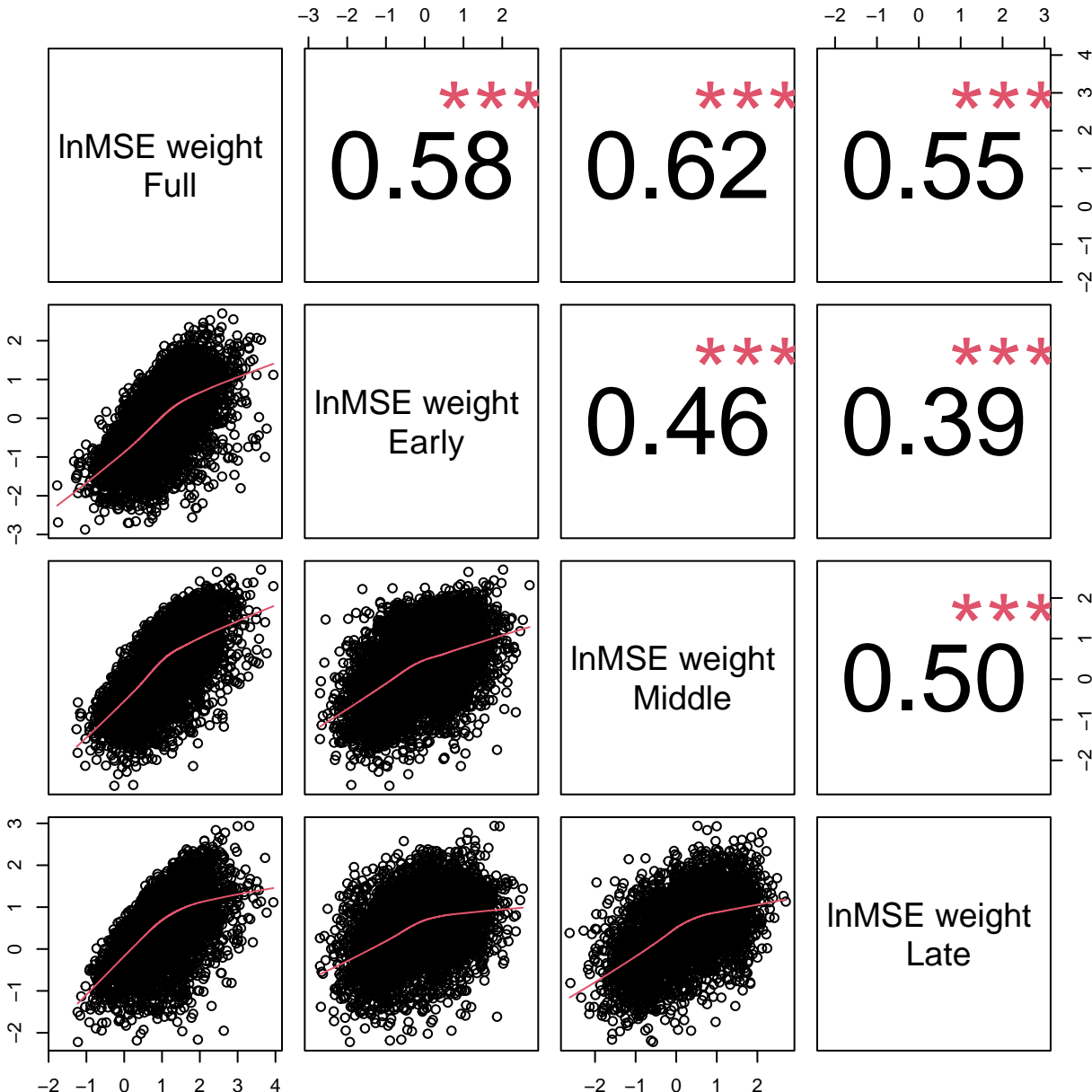

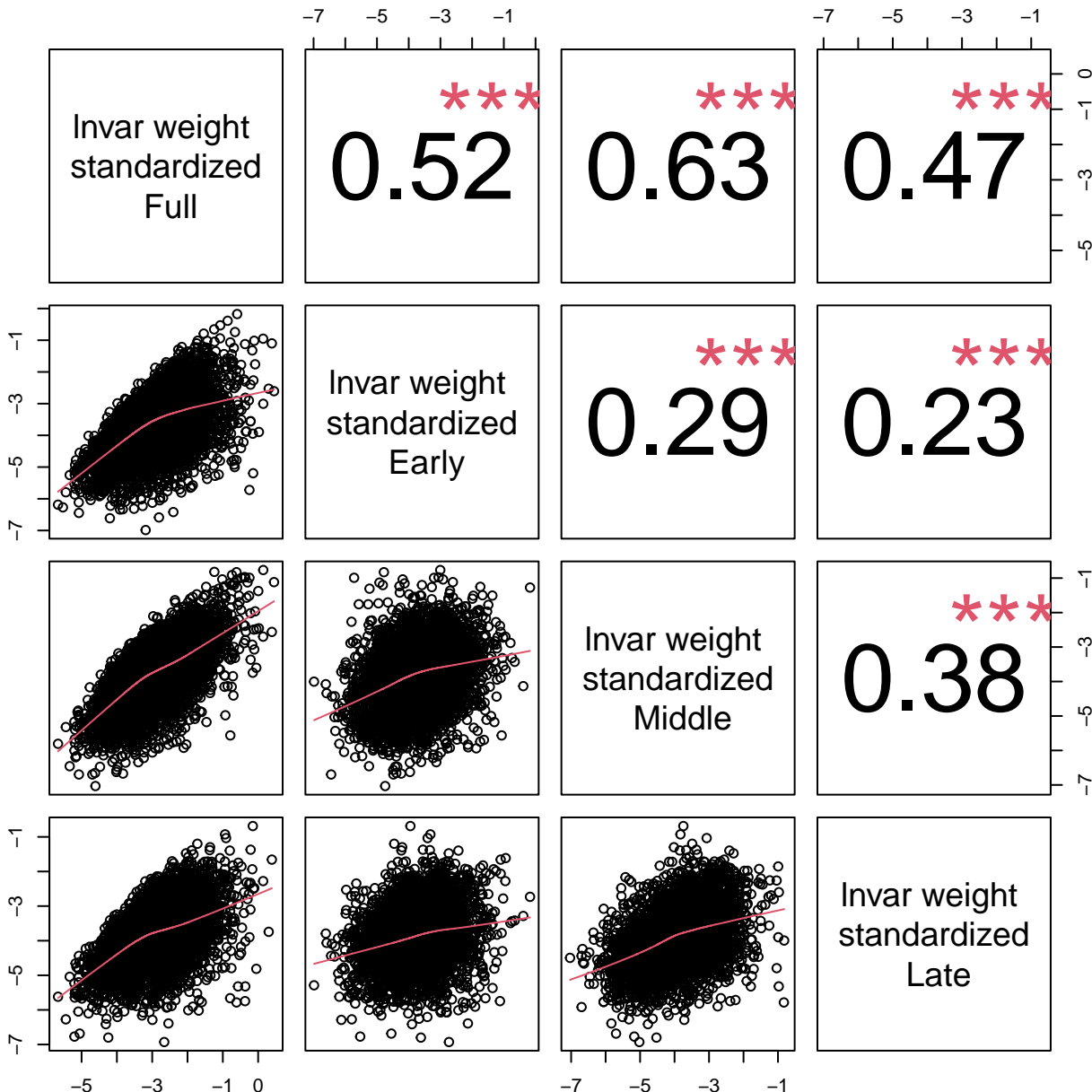

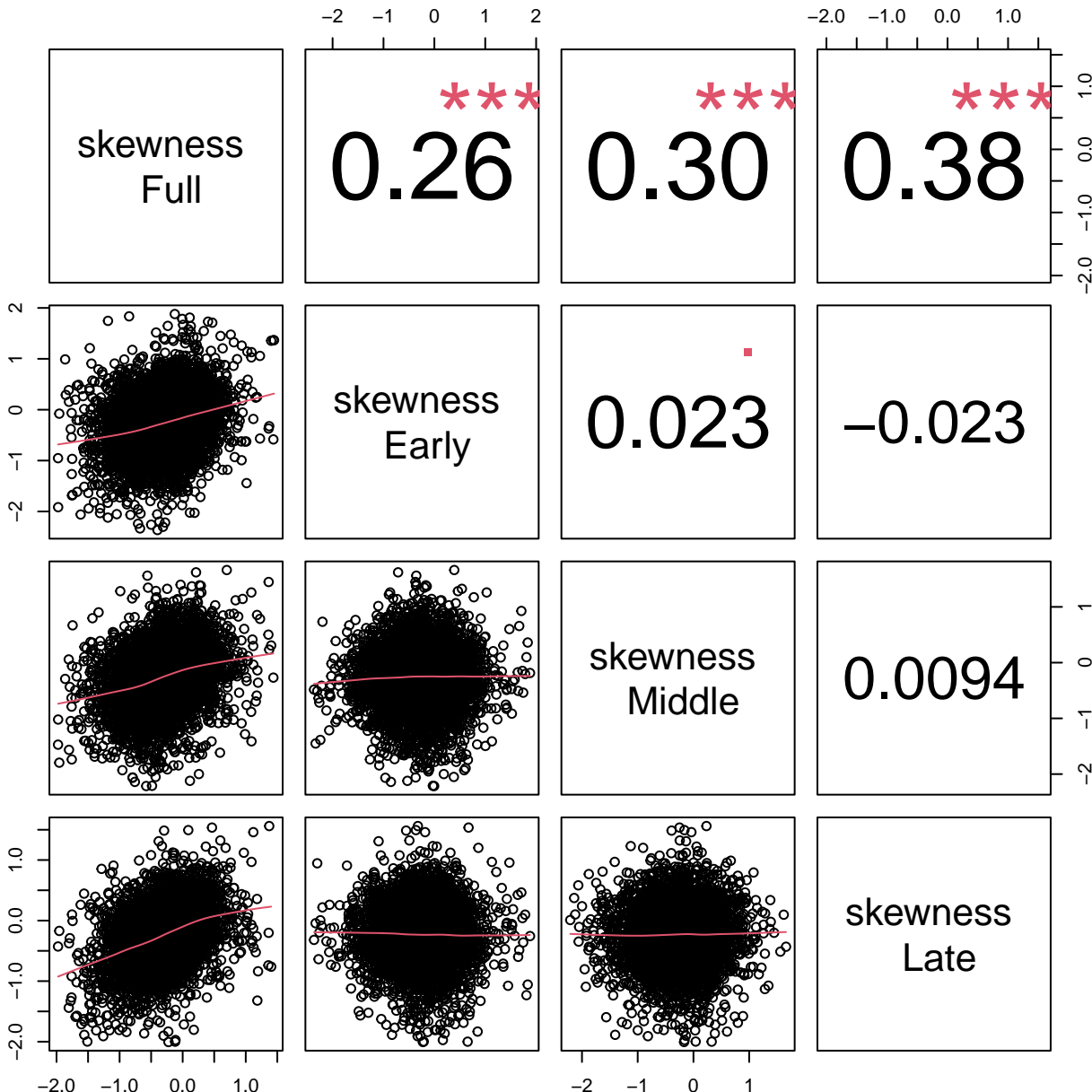

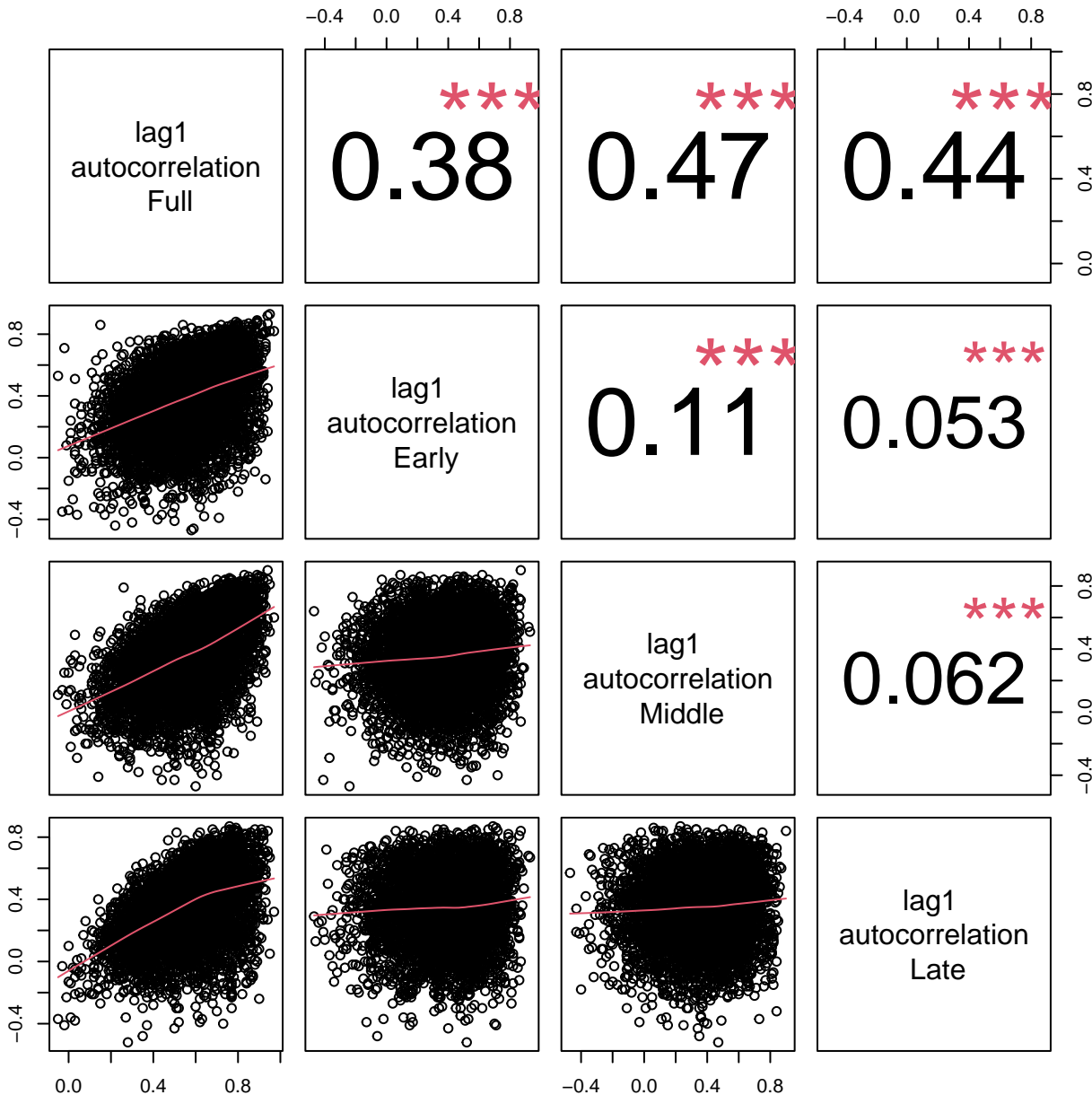



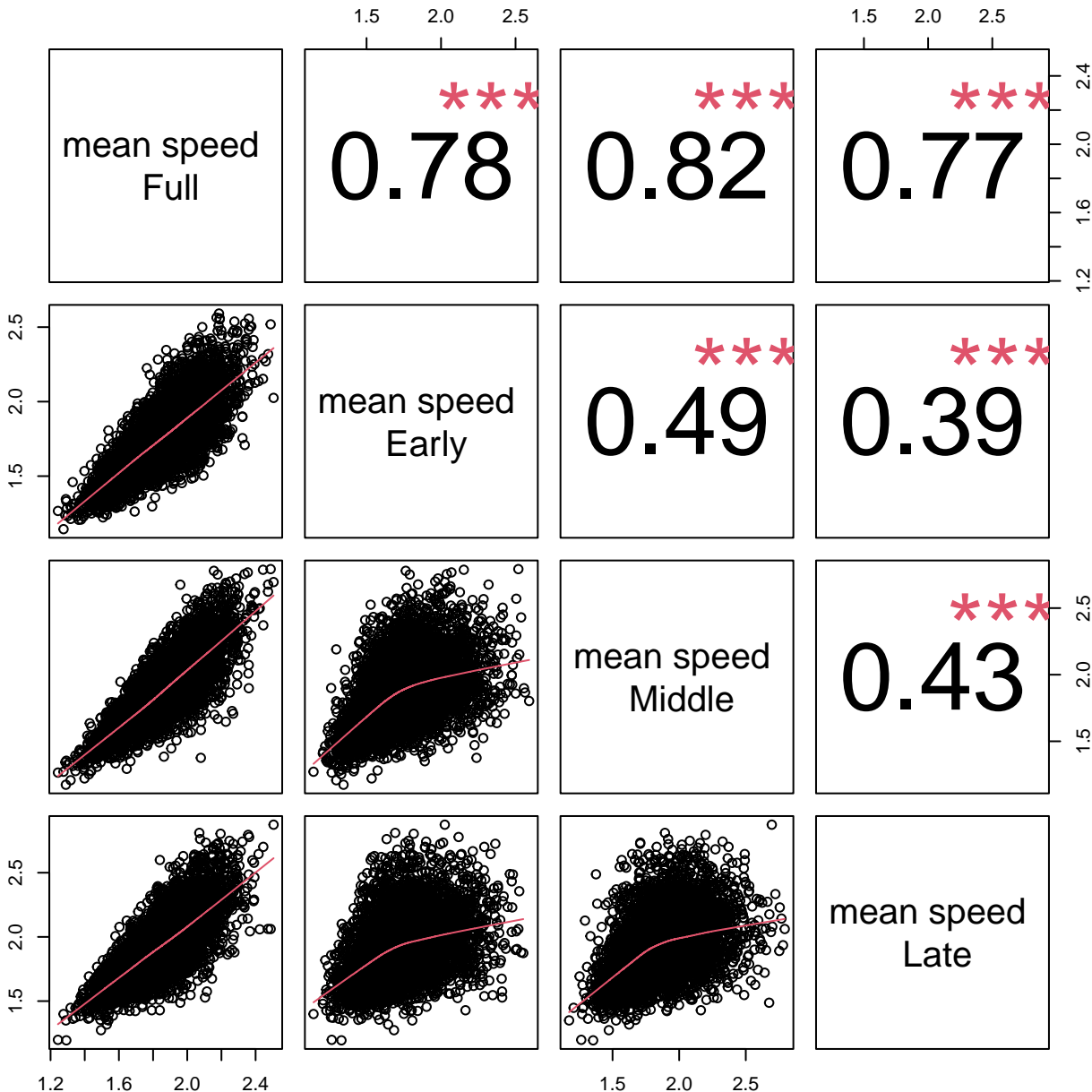

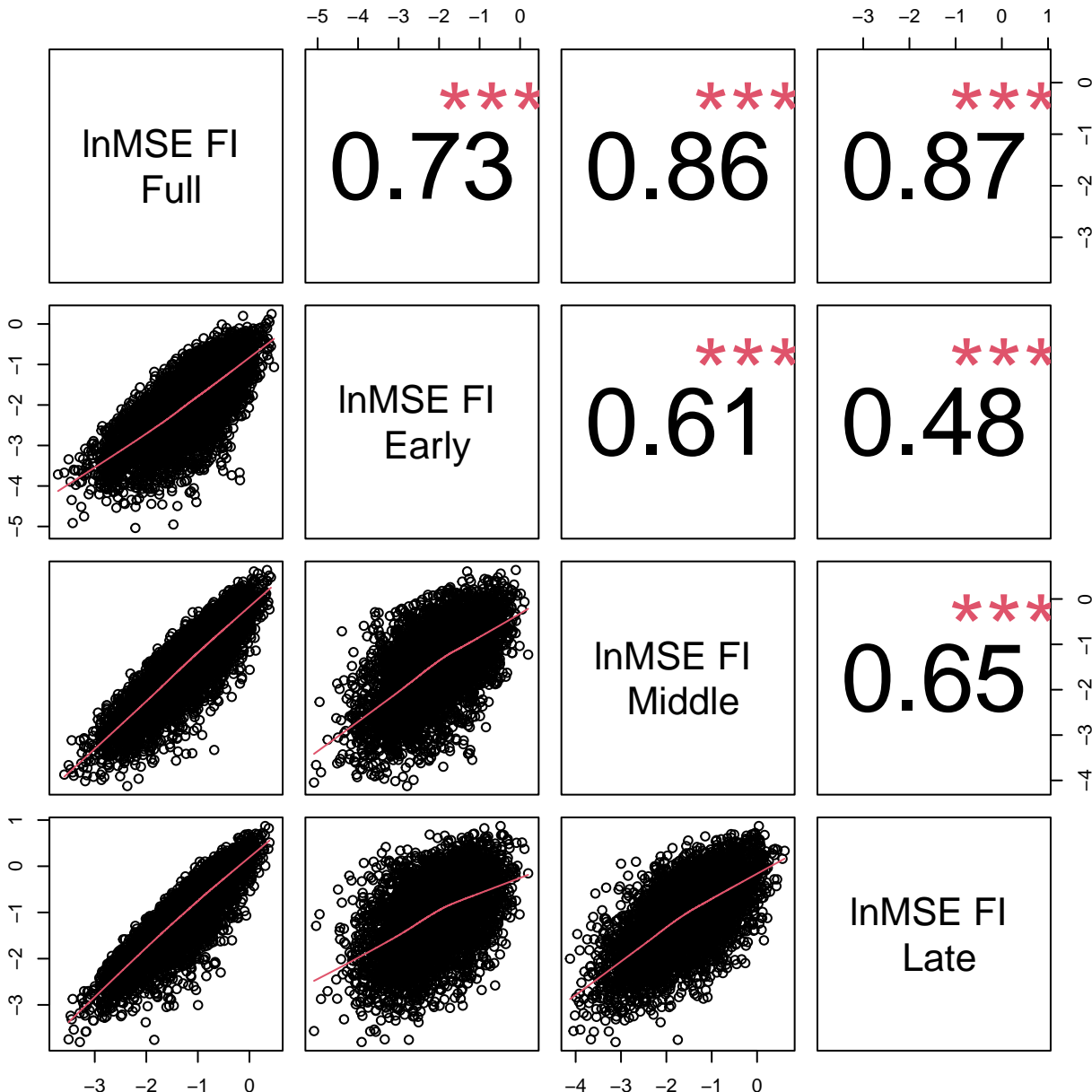

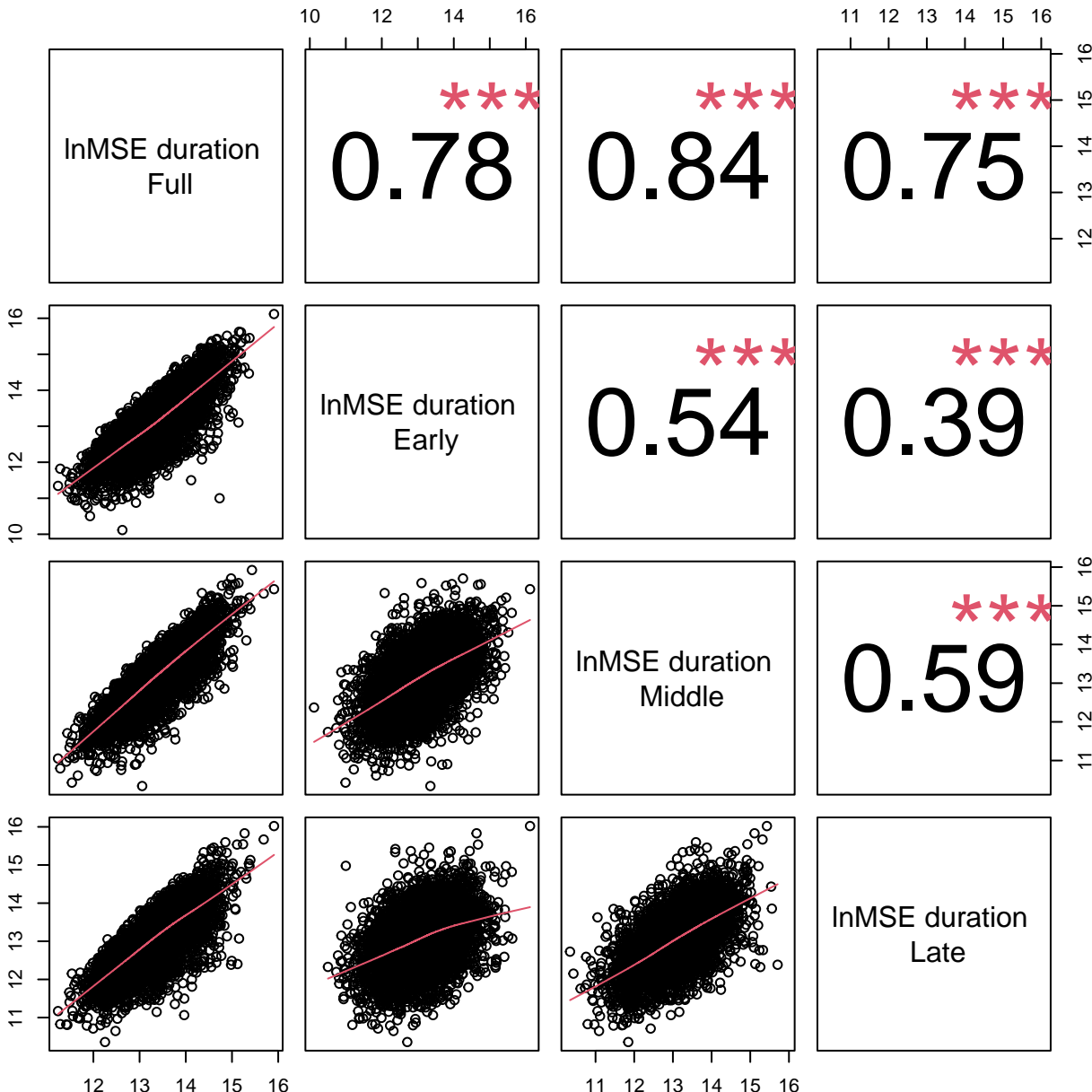

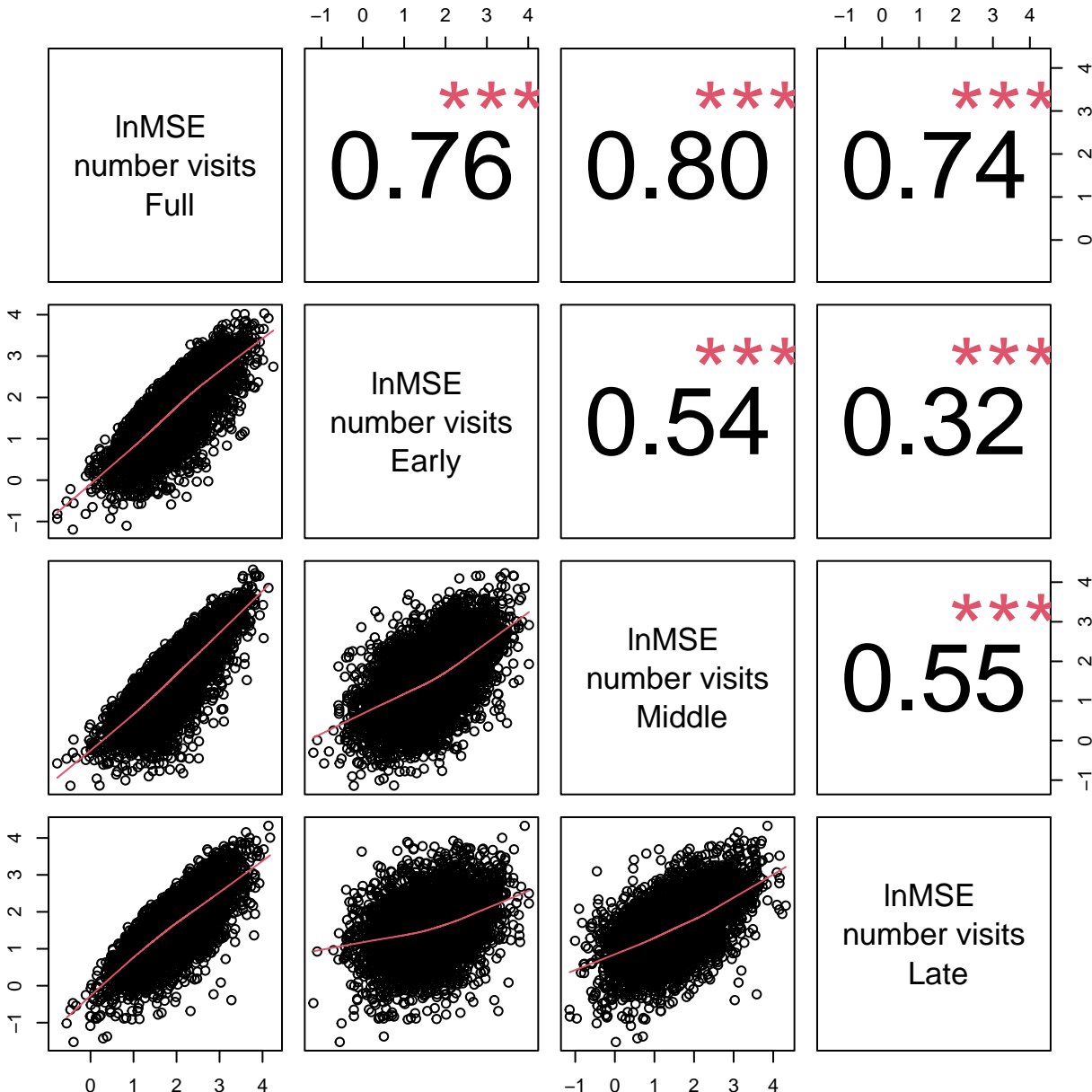



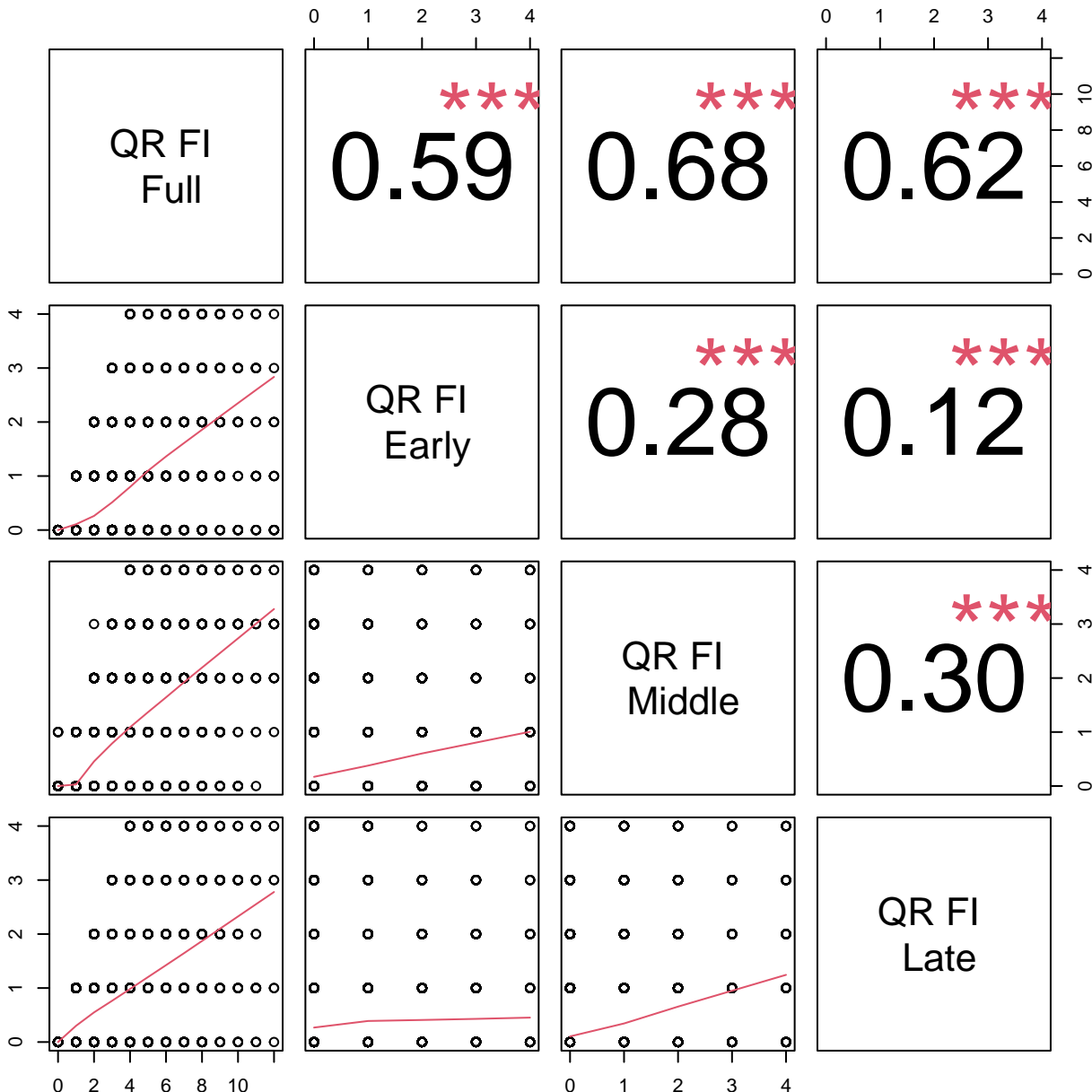

Supplement: Supplementary file 7 — Additional file 7: Fig. S4. Pairwise correlation plots for all evaluated traits with full datasets and reduced datasets. [file 40104_2023_901_MOESM7_ESM.pdf]
